# Author Correction: Lnc-mg is a long non-coding RNA that promotes myogenesis

**DOI:** 10.1038/s41467-025-63506-y

**Published:** 2025-09-10

**Authors:** Mu Zhu, Jiafan Liu, Jia Xiao, Li Yang, Mingxiang Cai, Hongyu Shen, Xiaojia Chen, Yi Ma, Sumin Hu, Zuolin Wang, An Hong, Yingxian Li, Yao Sun, Xiaogang Wang

**Affiliations:** 1 Guangdong Provincial Key Laboratory of Bioengineering Medicine & National Engineering Research Center of Genetic Medicine, Department of Cell Biology and Institute of Biomedicine, Jinan University, Huang-Pu Avenue West 601, Guangzhou, 510632 China; 2 State Key Laboratory of Space Medicine Fundamentals and Application, China Astronaut Research and Training Center, Beijing, 100094 China; 3 Preclinical Medical School, Beijing University of Chinese Medicine, Beijing, 100019 China; 4 State Key Discipline of Infectious Diseases, Shenzhen Third People’s Hospital, Shenzhen, 518116 China; 5 Shanghai Engineering Research Center of Tooth Restoration and Regeneration, School and Hospital of Stomatology, Tongji University, Shanghai, 200072 China

Correction to: *Nature Communications* 10.1038/ncomms14718, published online 10 March 2017

In the version of the article initially published, due to an error in figure preparation, the image shown for WT mice on the left side of Fig. 4c was mistakenly picked from denervated wild-type mice. The corrected Fig. 4c is shown below as Fig. 1. Due to the age of the article, the image cannot be replaced directly; this amendment serves to correct the article.

**Fig. 1:**
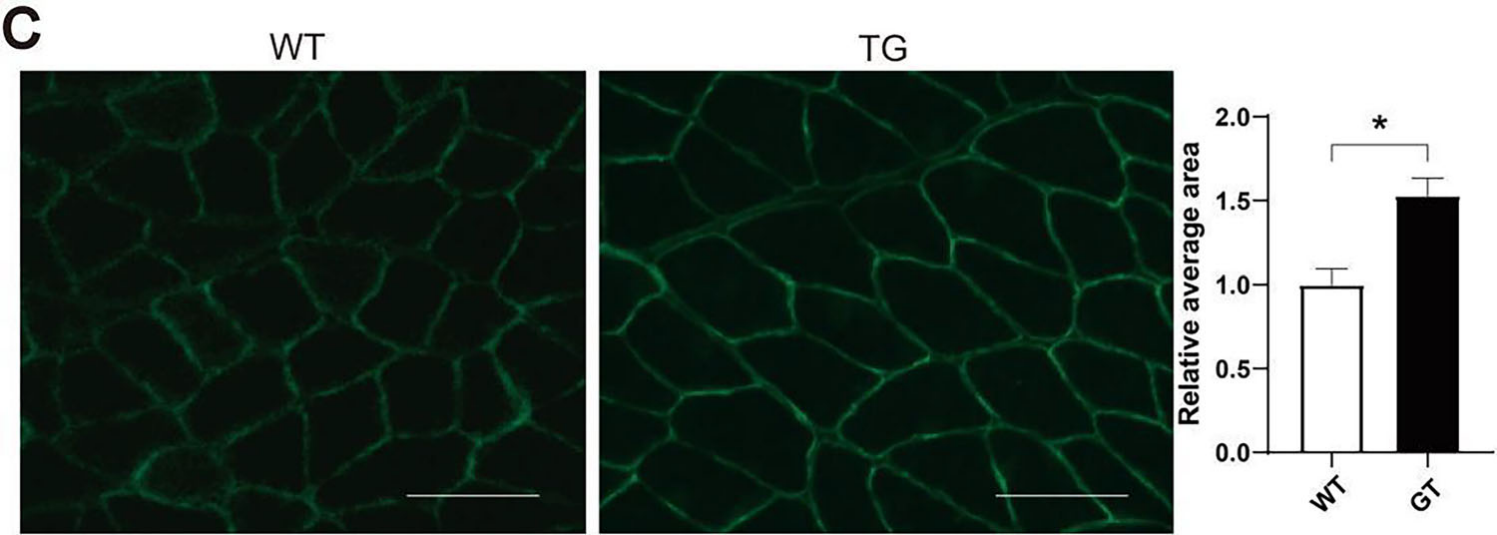
Corrected Fig. 4c.

