# Supplementary material for: Lnc-mg is a long non-coding RNA that promotes myogenesis
Source: Nat Commun. 2017 Mar 10;8:14718. doi: 10.1038/ncomms14718 (PMC5353601; doi:10.1038/ncomms14718)
Supplement: Supplementary Information — Supplementary Figures and Supplementary Tables. [file ncomms14718-s1.pdf]

a

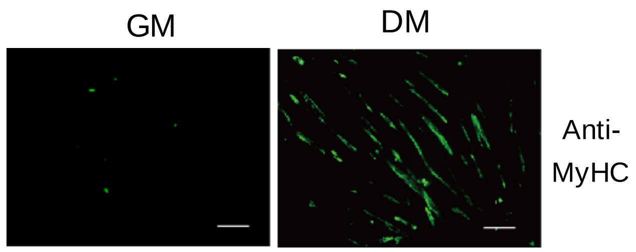

b

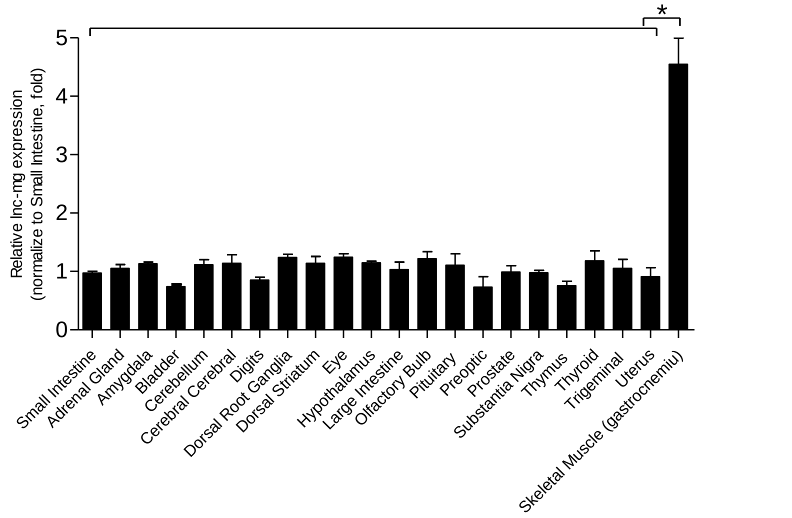

c

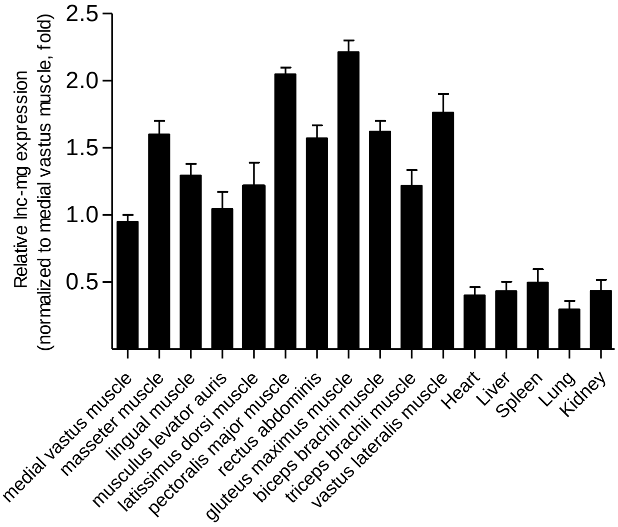

d

Summary of lnc-mg

Class	strand	length	organism	Cellular role	Molecular function
mRNA like lncRNA	_	884 nt	mouse	unknown	unknown

e

lnc-mg full-length (884 nt)

GGAA GGUCUA ACCAUGA GGAAG AAA GUGA AUUCUCAGAUUA GGCA GUGG AAG GGGUUG GUAGU AAG AGG AGA GGG AGUCAGGAC AGA GAUG AACUUGUCUGGCCCGUU UCUGGAGG GU
UUGUCAUAGACGUCUCCCUCGUGCACUGC AGGG GCAAC AGACUGCUGUGUGAU ACCCAUGA GUGUUUCUGCUGCAUCACG GAA GGA GAUAC AGAC AAG AUUUCCUAGG GUACAG GCAAA
GGCUCAAG GCUAGCG GUGACGCC AAUG AGG AUGG AUUGUUCAUAUCCCUUCAGG AAG GUGAUCA AGCAU GUCUCCAGGCUUCCCCGUCUUCCCUCAGGCAUGUGUGUG GUACUUACUG
GACUUGGCCUGGCUGCUCUCCAUGCUUA ACCUGGGUG ACUAA GGA GUUUGUCAUAG AUAUA AGCAC AAUG GUUCUUUUCUUUUUCCUCUCCCUGAUGUACAAAAU GAUC GGCCACCAUG
GAA AGG GGA AAG GA AGA GACUG ACGAGCUCUUGGC AGAU AA AUUUUUUAUCUCCCAAAGUUAUG AGUAC AA AACA GAGUG ACA AAG AUCUUCACAUUUUGCUCAUCUCUUUGGUCACUUU
CCUGCACUUGGAUCAAUC ACUCUUCGCUUAUGAUUUUGGUGUG AACCUCCCGGCUCUUCACCCGCAGCUUGUUGACCUG GGACUCCGCG AUGUCA GCCCGCUCCUCGGCUUCCUCCAG
CUCGUGCUGGAUCUUGCGG AAUUUGGCCA GGUUG ACAUUGG AUUGUUCCUCCUGUGAGUA AGA AUGCAUUUGUA AGG AA GAA AUGCUCACAUUUCUAG ACUUUAGG GU CAGUGCAUCUU
GGCUGUCUUUUUUGCUUAAAACCAAUCCACUCUAAUAAAUGUUCAAAGAA GUG

f

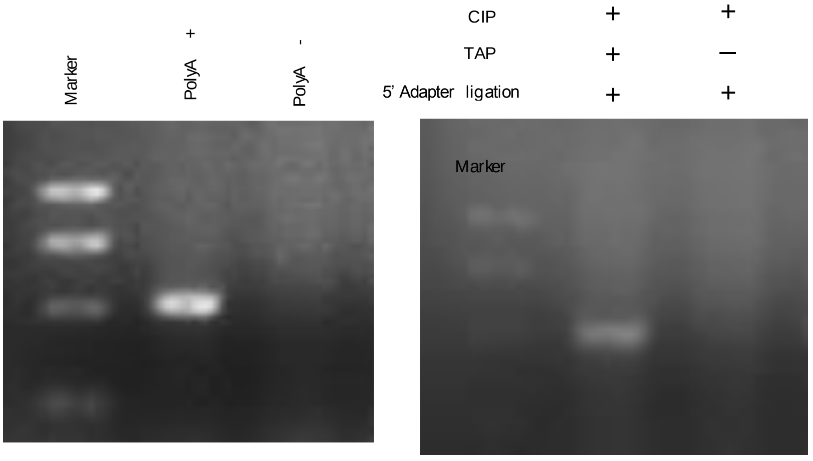

g

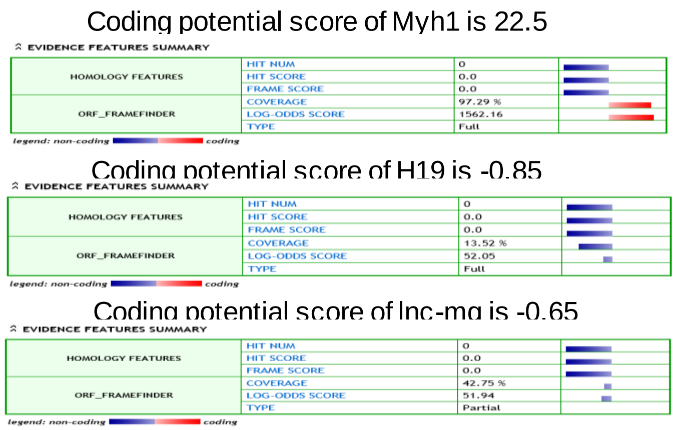

Supplementary Figure1. Characterization of lnc-mg. (**a**) MyHC immunostaining of undifferentiated muscle stem cells (GM) and five days of differentiated muscle stem cells (DM). Scale bar: 40 μm. (**b**) Real-time PCR analysis of lnc-mg expression in 22 types of mouse tissues. Mean values ± SEM, n=6, *P< 0.05. Mice were 8-week old, three for male, three for female. (**c**) Real-time PCR analysis of lnc-mg expression in 16 types of mouse skeletal muscles. Mean values ± SEM, n=6. Mice were 8-week old, three for male, three for female. (**d**) The molecular information of lnc-mg. (**e**) The transcript sequence of lnc-mg. (**f**) Left: real-time PCR analysis of lnc-mg expression in polyA+ and polyA- RNA fraction from muscle stem cells. Right: total RNA from muscle stem cells was treated with Calf Intestine Alkaline Phosphatase (CIP) to remove free 5'-P, then treated with Tobacco Acid Pyrophosphatase (TAP) to remove the cap structure and then a RNA adapter oligonucleotide was ligated to the RNA population using T4 RNA ligase (FistChoice RLM-RACE Kit, Ambion). (**g**) Bioinformatics analysis of the coding capability of *Myh1*, *H19* and lnc-mg. The data statistical significance is assessed by Student's *t*-test.

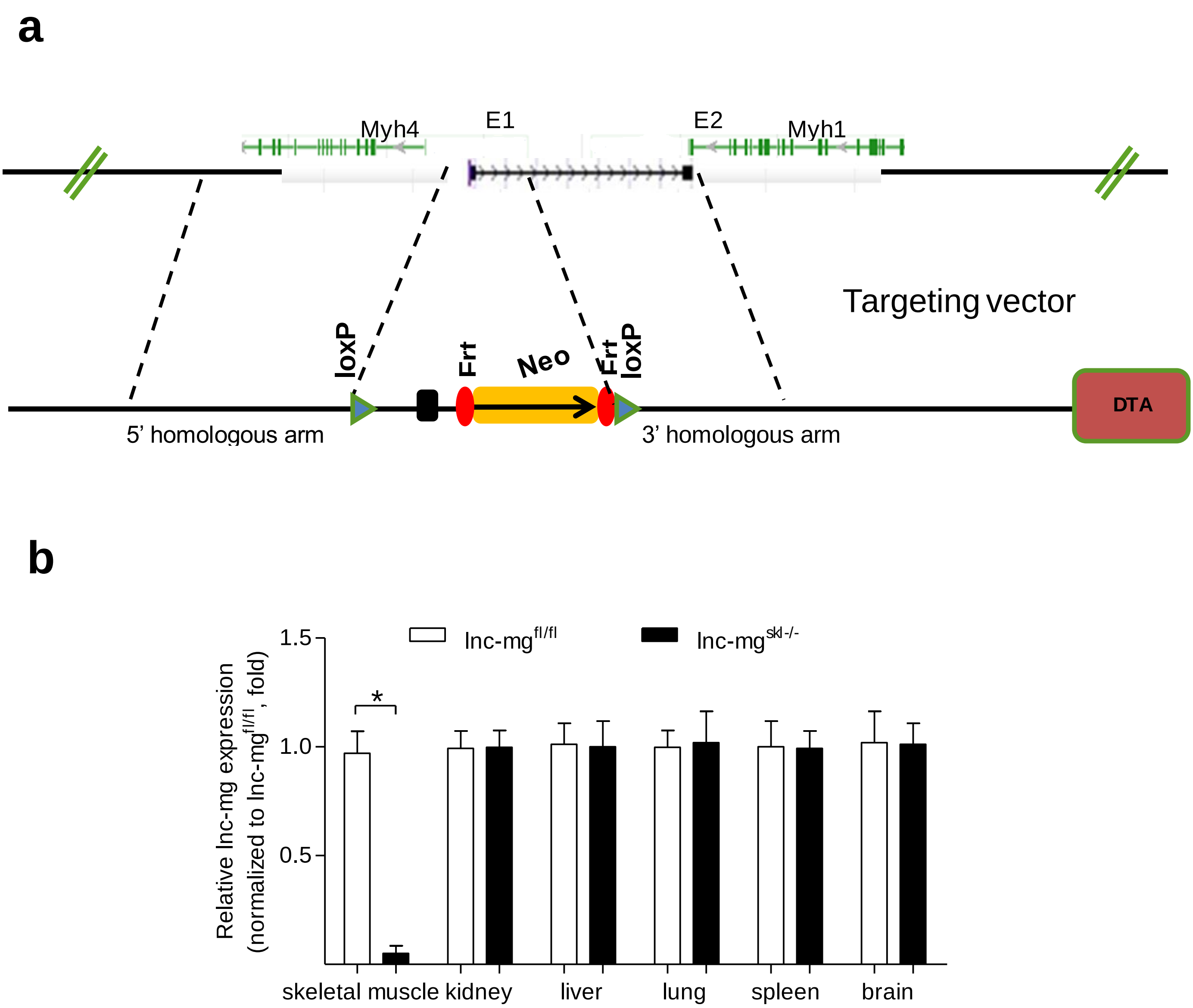

Supplementary Figure 2. Generation of *lnc-mg*$^{skl-/-}$ mice. (**a**) Targeting strategy for lnc-mg conditional knockout in mouse. (**b**) Real-time PCR analysis of lnc-mg expression in mouse tissues from *lnc-mg*$^{fl/fl}$ mice and *lnc-mg*$^{skl-/-}$ mice. Mean values ± SEM, n=4, **P*< 0.05. The data statistical significance is assessed by Student’s *t*-test.

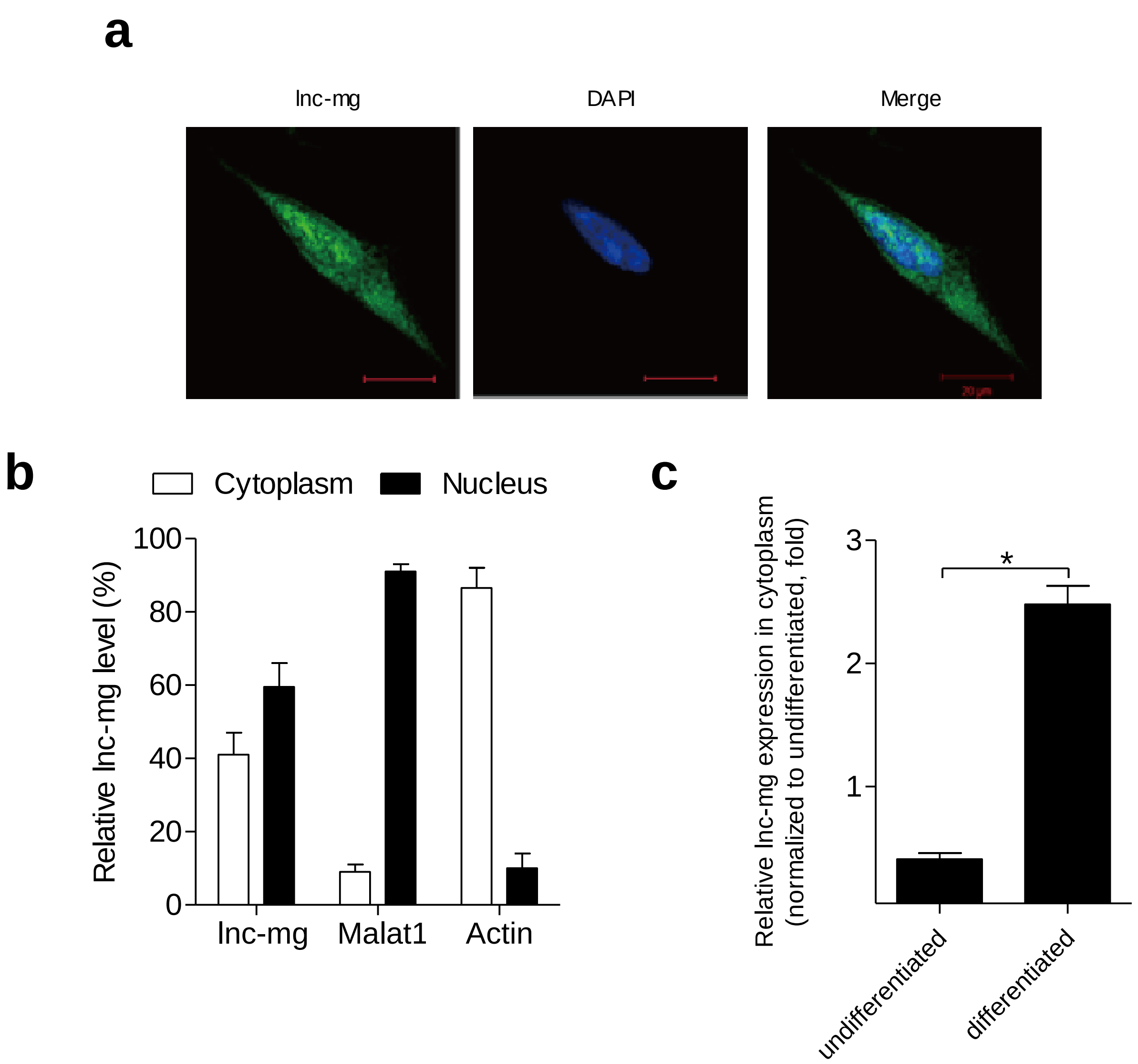

Supplementary Figure 3. The location lnc-mg in cytoplasm. (**a**) The subcellular localization of lnc-mg in C2C12 cells determined by fluorescent *in situ* hybridization (FISH). Scale bar: 20 μm. (**b**) Real-time PCR analysis of the subcellular localization of lnc-mg in C2C12 cells. Mean values ± SEM, n=4. (**c**) Real-time PCR analysis of lnc-mg level in cytoplasm of undifferentiated C2C12 cells and four days differentiated C2C12 cells. Mean values ± SEM, n=4, *$P < 0.05$. The data statistical significance is assessed by Student's *t*-test.

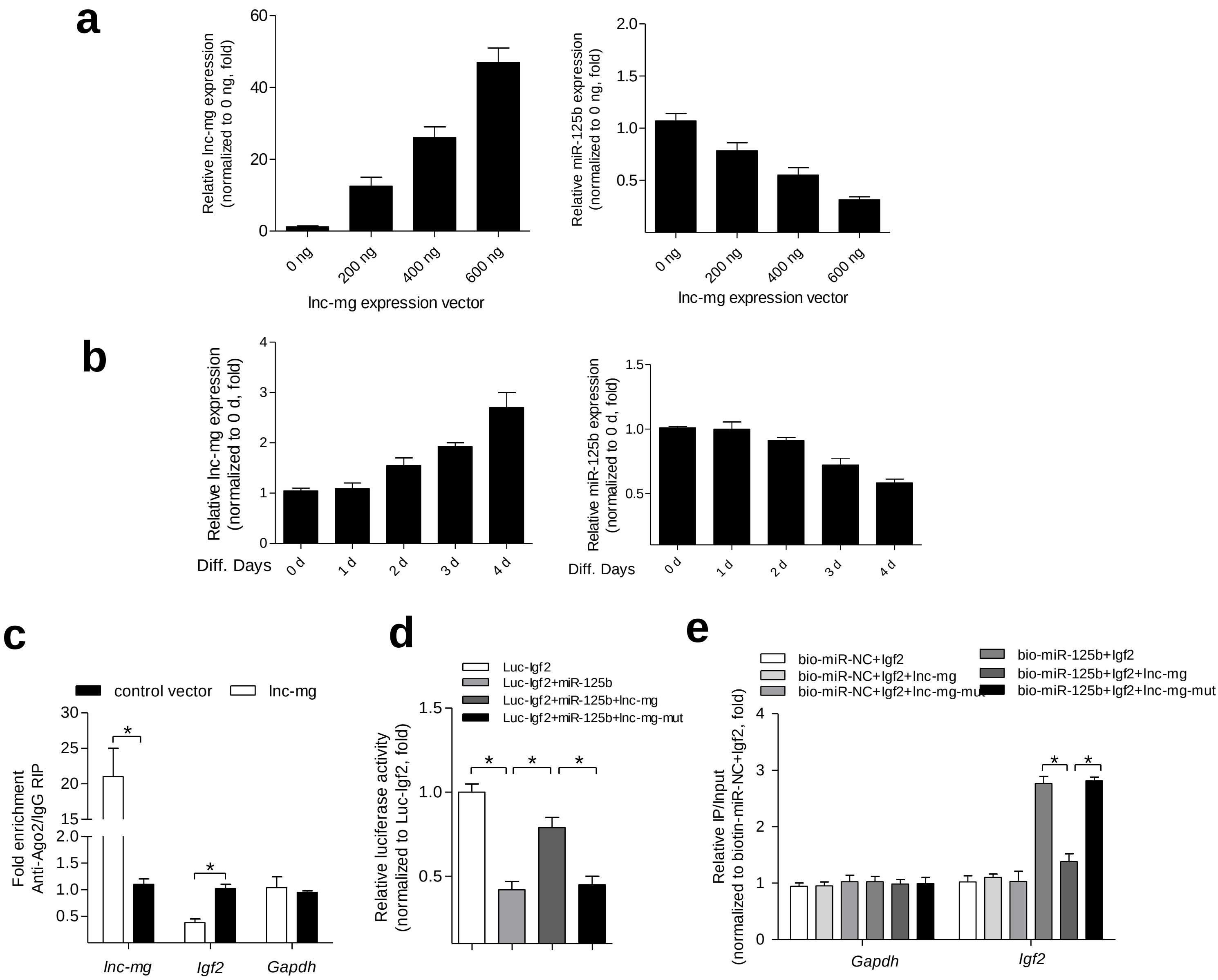

Supplementary Figure 4. lnc-mg regulates Igf2 expression by competing for miR-125b. (**a**) Real-time PCR analysis of the expression of lnc-mg and miR-125b in C2C12 cells after transfected with 200 ng, 400 ng or 600 ng lnc-mg expression vector for 48 h. Mean values ± SEM, n=4. (**b**) Real-time PCR analysis of the relative expression of lnc-mg and miR-125b during C2C12 cells myogenic differentiation. Mean values ± SEM, n=4. (**c**) RIP assay of the enrichment of Ago2 on lnc-mg, *Igf2* and *Gapdh* relative to IgG in C2C12 cells transfected with lnc-mg empty vector (control vector) or lnc-mg overexpression vector (lnc-mg). Mean values ± SEM, n=4, **P*< 0.05. (**d**) The relative luciferase activity of psiCHECK-2 containing *Igf2* 3'UTR co-transfected with miR-125b, miR-125b and lnc-mg, miR-125b and mutated lnc-mg respectively. Mean values ± SEM, n=4, **P*< 0.05. (**e**) Streptavidin capture was performed for C2C12 myoblasts co-transfected with biotin-miR-NC or biotin-miR-125b and *Igf2* 3'UTR, *Igf2* 3'UTR with lnc-mg or *Igf2* 3'UTR with lnc-mg-mut, followed by real-time PCR to detect *Igf2* mRNA and *Gapdh* mRNA levels.

Supplementary Figure 5 Unprocessed scans of Western blots

Figure 5k

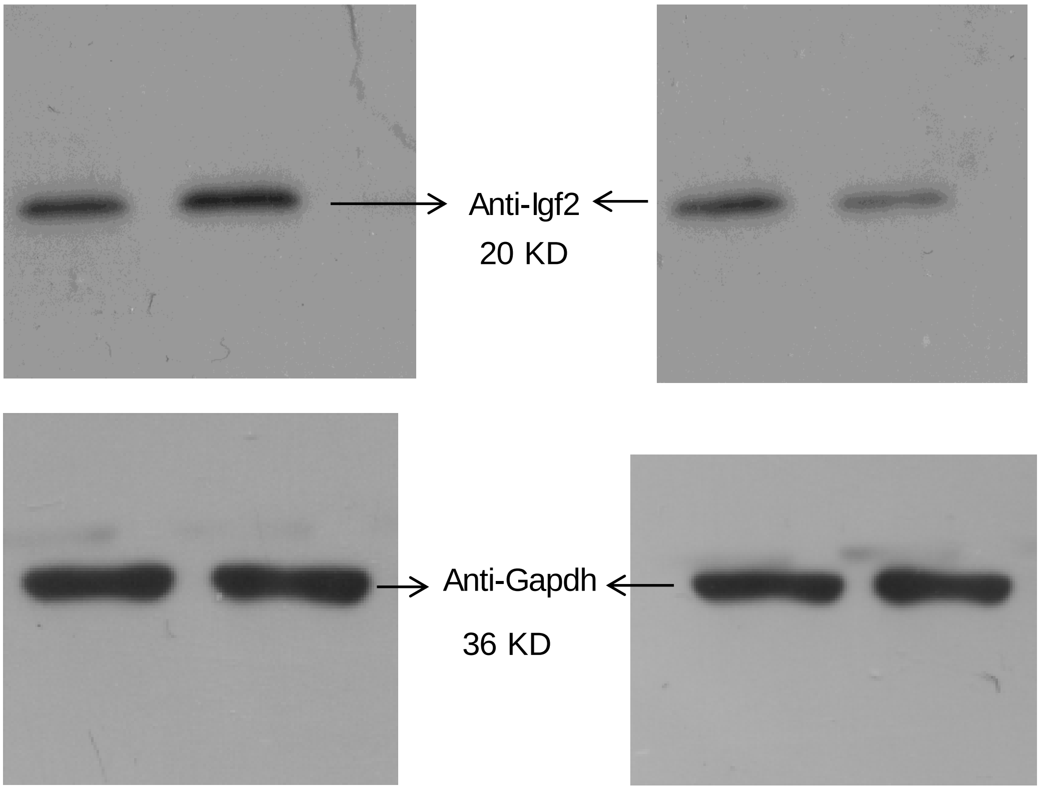

Figure 6b

Anti-Igf2

20 KD

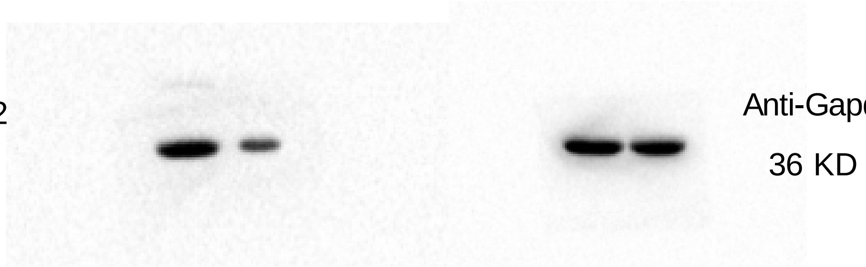

Anti-Gapdh

36 KD

Figure 6e

Anti-Igf2

20 KD

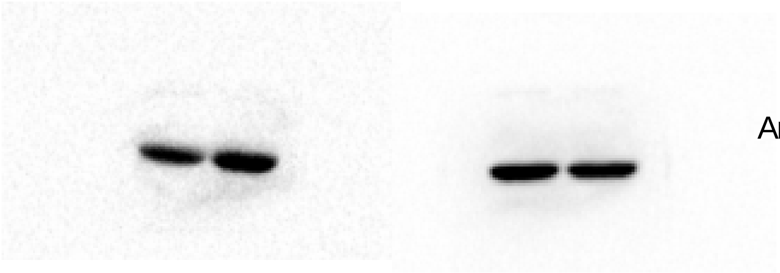

Anti-Gapdh

36 KD

Supplementary Table 1 Differently expressed lncRNA determined by microarray

	log2					
ID	GM	DM	DM/G M	Regulati on	transcription id	gene name
RB_p_00010001 8	10.511 09	14.954 44	4.4433 45	up	XM_915111.5	LOC636306
RB_p_00011281 7	8.7824 52	12.766 74	3.9842 84	up	XM_907182.4	LOC631966
RB_p_00010926 9	7.6073 3	11.439 31	3.8319 81	up	XM_003946264.1	LOC1000461 51
RB_p_00012314 7	7.7857 25	11.400 52	3.6147 99	up	NR_001592.1	H19
RB_p_00014612 9	10.666 22	14.230 18	3.5639 59	up	XM_001002242.3	Gm8430
RB_p_00014612 3	11.141 47	14.533 74	3.3922 67	up	XM_973722.2	Gm7429
RB_p_00013113 9	11.124 32	14.257 98	3.1336 59	up	NM_001177468.1	Gm7325
RB_p_00011097 2	8.6159 36	11.746 09	3.1301 58	up	XM_891806.3	Gm6570
RB_p_00016248 1	12.658 17	15.762 84	3.1046 66	up	XR_001564.2	Gm6252
RB_p_00012548 5	8.6546 36	11.725 79	3.0711 56	up	XM_003086679.3	Gm5908
RB_p_00011015 0	10.195 99	13.240 74	3.0447 54	up	XR_107848.2	Gm5879
RB_p_00011861 9	10.944 47	13.808 61	2.8641 44	up	NM_001033297.2	Gm561
RB_p_00015022 3	11.851 36	14.702 98	2.8516 26	up	XM_484272.5	Gm5451
RB_p_00015065 0	9.4945 22	12.244 76	2.7502 38	up	XM_356811.4	Gm5215
RB_p_00014038 1	12.556 51	15.257 42	2.7009 19	up	XM_003945622.1	Gm15453
RB_p_00011158 3	8.6438 56	11.330 92	2.6870 61	up	XM_001476804.2	Gm12816
RB_p_00010311 3	8.7670 81	11.448 12	2.6810 35	up	NM_001122660.1	Gm10639
RB_p_00011261 0	10.164 6	12.845 3	2.6807 09	up	NR_002885.3	Gm10052
RB_p_00016182 7	12.916 56	15.575 6	2.6590 35	up	XM_001480280.2	Gm10045
RB_p_00014402	12.060	14.681	2.6202	up	F830221I04	AK172626

5	92	22	99			
RB_p_00011974<br>3	7.8968<br>37	10.494<br>86	2.5980<br>19	up	F830207P20	AK172445
RB_p_00012908<br>9	11.485<br>33	14.073<br>58	2.5882<br>58	up	F830119M20	AK172311
RB_p_00011972<br>4	7.9088<br>93	10.496<br>52	2.5876<br>28	up	F830019N15	AK171928
RB_p_00013726<br>7	12.261<br>12	14.829<br>28	2.5681<br>61	up	G830036B20	AK166314
RB_p_00013779<br>9	12.018<br>08	14.508<br>43	2.4903<br>5	up	F530207G06	AK165502
RB_p_00012164<br>6	7.4757<br>33	9.9376<br>19	2.4618<br>86	up	D630009K18	AK164696
RB_p_00012751<br>7	8.7846<br>35	11.212<br>7	2.4280<br>64	up	8230402K13	AK162281
RB_p_00010100<br>2	9.1232<br>59	11.549<br>14	2.4258<br>84	up	F930025D07	AK158068
RB_p_00008889<br>7	9.4171<br>49	11.824<br>43	2.4072<br>79	up	I920040M19	AK146676
RB_p_00016372<br>1	9.4297<br>55	11.817<br>78	2.3880<br>28	up	I730048I08	AK146302
RB_p_00014635<br>8	10.841<br>83	13.229<br>02	2.3871<br>93	up	C530009H14	AK141413
RB_p_00011862<br>6	8.3677<br>79	10.715<br>68	2.3478<br>97	up	C130037G23	AK140828
RB_p_00009895<br>2	10.554<br>27	12.899<br>11	2.3448<br>36	up	A430085M09	AK138821
RB_p_00011510<br>7	8.8084<br>28	11.144<br>66	2.3362<br>3	up	9230023I17	AK136693
RB_p_00011973<br>5	7.9753<br>7	10.245<br>55	2.2701<br>82	up	7420409C10	AK135737
RB_p_00009319<br>4	8.7824<br>52	11.015<br>42	2.2329<br>63	up	5330435P14	AK133680
RB_p_00010012<br>8	10.533<br>33	12.763<br>49	2.2301<br>59	up	4932427A08	AK133261
RB_p_00012178<br>6	9.5124<br>11	11.725<br>22	2.2128<br>13	up	E430002O04	AK088055
RB_p_00016291<br>8	10.353<br>51	12.544<br>12	2.1906<br>05	up	C130078C04	AK081798
RB_p_00015914<br>9	10.576<br>48	12.719<br>17	2.1426<br>91	up	C130071K02	AK081720
RB_p_00016366<br>3	10.620<br>22	12.756<br>14	2.1359<br>19	up	A730075L20	AK080530

RB_p_000126255	9.957586	12.09204	2.134456	up	AK078704	AK078704
RB_p_000126254	9.982043	12.11342	2.131374	up	6230412L15	AK078021
RB_p_000109875	8.549464	10.65075	2.101289	up	E330032N24	AK054496
RB_p_000109697	9.475058	11.56621	2.091155	up	E230013J01	AK054033
RB_p_000121106	10.09188	12.13507	2.043192	up	C920030K03	AK050666
RB_p_000099785	7.79658	9.837628	2.041047	up	C730047G17	AK050424
RB_p_000140422	8.691162	10.71296	2.021795	up	C130049E09	AK048321
RB_p_000099993	9.62022	11.63972	2.019498	up	A630028C14	AK041654
RB_p_000122788	9.72565	11.7333	2.007648	up	A630012F06	AK041461
RB_p_000119778	7.674781	9.675369	2.000588	up	A430102C15	AK040479
RB_p_000134454	8.327927	10.32605	1.998128	up	A130053K16	AK037838
RB_p_000098335	7.99812	9.978234	1.980114	up	9930028G15	AK036940
RB_p_000131146	8.982043	10.95201	1.96997	up	5930427B08	AK031194
RB_p_000127270	7.646259	9.561606	1.915347	up	4732467D23	AK028893
RB_p_000087893	8.156504	10.06474	1.908238	up	4930452A19	AK019626
RB_p_000100936	8.357552	10.26053	1.902976	up	6330417L24	AK018194
RB_p_000104094	8.440177	10.32418	1.884003	up	5830469G19	AK018045
RB_p_000099976	8.707359	10.58871	1.881356	up	4933433P14	AK017049
RB_p_000119296	8.863154	10.73188	1.868731	up	3300002L04	AK014372
RB_p_000097732	8.787903	10.57648	1.788582	up	2900053I11	AK013679
RB_p_000097035	7.908893	9.663558	1.754665	up	2810047D15	AK012912
RB_p_000128491	7.922832	9.665928	1.743096	up	AK011683	AK011683

RB_p_000119053	7.774787	9.5157	1.740913	up	2410006H16	AK010427
RB_p_000105356	8.327927	10.05528	1.727356	up	2310058C22	AK009974 (lnc-mg)
RB_p_000135380	8.424866	10.13614	1.711271	up	2210408N17	AK008861
RB_p_000089525	7.8095	9.466246	1.656746	up	1700047O18	AK006720
RB_p_000097577	8.247928	9.89986	1.651933	up	1700003H21	AK005641
RB_p_000150942	7.820179	9.388017	1.567838	up	1110038B12	AK004150
RB_p_000091975	7.634206	9.172428	1.538221	up	1110007F12	AK003535
RB_p_000104279	9.182394	7.15313	-2.02926	down	1110005B01	AK003454
RB_p_000118989	10.1472	8.075033	-2.07217	down	1110002C15	AK003280
RB_p_000101564	10.29194	8.201307	-2.09063	down	1100001I11	AK003172
RB_p_000140319	11.09099	8.735838	-2.35516	down	0610038G21	AK002796
RB_p_000141894	10.56605	8.156504	-2.40955	down	NR_073462.1	5730488B01 Rik
RB_p_000148444	11.98276	9.5157	-2.46706	down	NM_183104.2	4931429L15 Rik
RB_p_000121695	12.18498	9.670656	-2.51432	down	NR_030738.1	2410006H16 Rik
RB_p_000096731	10.39732	7.857981	-2.53934	down	NR_038151.1	2410004N09 Rik
RB_p_000100408	10.93688	8.296151	-2.64073	down	NR_027820.1	1810032O08 Rik
RB_p_000094370	12.48545	9.768736	-2.71672	down	NM_028046.1	1600014K23 Rik
RB_p_000160293	12.17264	9.385143	-2.78749	down	NR_015536.1	1110038B12Rik
RB_p_000150252	11.47184	8.156504	-3.31534	down	NM_024179.5	0610009O20 Rik

Supplementary Table 2 Primers and shRNA sequences used in this study

Genes (mmu)		sequence	accession number	product lengths
lnc-mg	forward	5'-CTGCATCACGGAAGGAGATA-3'	N/A	89 bp
	reverse	5'-AACAATCCATCCTCATTGGC-3'		
MyoD	forward	5'-GGGCCGCTGTAATCCATCATG-3'	NM_010866.2	121 bp
	reverse	5'-CTGCCTTCTACGCACCTGGA-3'		
MyoG	forward	5'-AAGTGAATGAGGCCTTCGAG-3' (ref 49)	NM_031189.2	178 bp
	reverse	5'- AGATTGTGGGCGTCTGTAGG -3'		
miR-125b	RT primer	5'-GTCGTATCCAGTGCAGGGTCCGAGGTATTCGCACTGGATACGACTCACAA-3'	NR_029822.1	
miR-125b	forward	5'-CACGCATCCCTGAGACCC-3'	NR_029822.1	59 bp
	reverse	5'-CCAGTGCAGGGTCCGAGGTA-3'		
U6	forward	5'-CGCTTCGGCAGCACATATA-3'	NR_003027.2	87 bp
	reverse	5'-TTCACGAATTTGCGTGTCAT-3'		
Gapdh	forward	5'-TCACCACCATGGAGAAGGC-3'	NM_001289726.1	169 bp
	reverse	5'-GCTAAGCAGTTGGTGGTGCA-3'		
lnc-mg	shRNA	GAGGATGGATTGTTCATATCC	N/A	

Supplementary Table 3 Differently expressed miRNA determined by microarray

ID_REF	Name	shRNA	shRNA-NC	lnc-mg	control
RB_p_mrmir000000979	mmu-let-7a-1-3p:MIMAT0004620;<br>mmu-let-7c-2-3p:MIMAT0005439	3.227034	3.008375129	6.548451	2.38207
RB_p_mrmir0000001071	mmu-let-7a-2-3p:MIMAT0017015	7.720342	4.722111186	6.637939	5.274966
RB_p_mrmir0000001899	mmu-let-7a-5p:MIMAT0000521	4.892194	4.797776641	8.37922	3.675846
RB_p_mrmir000000975	mmu-let-7b-3p:MIMAT0004621	8.091048	6.977980915	6.654627	7.025056
RB_p_mrmir0000001901	mmu-let-7b-5p:MIMAT0000522	9.025856	8.588755276	9.850291	9.604672
RB_p_mrmir0000001070	mmu-let-7c-1-3p:MIMAT0004622	2.741512	6.818973262	5.82493	3.332947
RB_p_mrmir0000001900	mmu-let-7c-5p:MIMAT0000523	6.801274	6.350863977	6.469446	7.067119
RB_p_mrmir000000985	mmu-let-7d-3p:MIMAT0000384	7.197653	1.985534057	4.227622	8.644288
RB_p_mrmir000000319	mmu-let-7d-5p:MIMAT0000383	6.0825	4.351857125	5.365724	2.065209
RB_p_mrmir000000986	mmu-let-7e-3p:MIMAT0017016	6.30831	5.37789906	6.233349	5.691887
RB_p_mrmir0000001895	mmu-let-7e-5p:MIMAT0000524	6.940103	6.920868814	6.236967	7.349212
RB_p_mrmir000000981	mmu-let-7f-1-3p:MIMAT0004623	6.785132	2.248253848	5.874908	5.382959
RB_p_mrmir000000982	mmu-let-7f-2-3p:MIMAT0017017	5.030245	3.254532927	6.716674	0.357072
RB_p_mrmir0000001898	mmu-let-7f-5p:MIMAT0000525	4.466693	7.525638308	5.145362	4.665605
RB_p_mrmir000000278	mmu-let-7g-3p:MIMAT0004519	4.779796	2.825972308	5.658021	2.665997
RB_p_mrmir0000001903	mmu-let-7g-5p:MIMAT0000121	7.970647	6.268583197	4.046916	0.632826
RB_p_mrmir0000001039	mmu-let-7i-3p:MIMAT0004520	2.684402	3.733538549	6.849533	3.654278
RB_p_mrmir0000001904	mmu-let-7i-5p:MIMAT0000122	7.149851	4.425593031	5.483856	8.134591
RB_p_mrmir0000001905	mmu-let-7j:MIMAT0025123	5.510354	2.639840197	4.564045	5.426135
RB_p_mrmir0000001896	mmu-let-7k:MIMAT0025580	8.757121	6.155919798	9.413858	9.347909
RB_p_mrmir000000159	mmu-miR-100-3p:MIMAT0017051	4.898256	9.415501191	3.565961	2.254341
RB_p_mrmir00000054	mmu-miR-100-5p:MIMAT0000655	6.909491	6.714722272	5.015941	3.497214
RB_p_mrmir0000001458	mmu-miR-101a-3p:MIMAT0000133	2.801799	6.941124746	7.39525	5.038041
RB_p_mrmir0000001678	mmu-miR-101a-5p:MIMAT0004526	5.827993	9.078426841	8.371693	3.83203
RB_p_mrmir0000001338	mmu-miR-101b-3p:MIMAT0000616	-0.43048	5.050146647	7.664202	3.127091
RB_p_mrmir0000001751	mmu-miR-101b-5p:MIMAT0017046	5.369887	6.911711315	6.347867	6.518936
RB_p_mrmir000000182	mmu-miR-101c:MIMAT0019349	3.872241	3.96371146	6.698134	1.103326
RB_p_mrmir0000001283	mmu-miR-103-1-5p:MIMAT0017024	8.562867	4.487177503	6.423734	5.762944
RB_p_mrmir000000367	mmu-miR-103-2-5p:MIMAT0017025	8.461979	1.513414886	6.795665	4.213999
RB_p_mrmir000000337	mmu-miR-103-3p:MIMAT0000546	7.430122	5.996122047	4.678455	6.79903
RB_p_mrmir000000802	mmu-miR-105:MIMAT0004856	6.50126	8.332931779	6.576291	5.596896
RB_p_mrmir000000258	mmu-miR-106a-3p:MIMAT0017009	6.566806	7.373017363	6.39234	3.185568
RB_p_mrmir000000617	mmu-miR-106a-5p:MIMAT0000385	8.124651	7.114682704	6.780888	4.78446
RB_p_mrmir000000860	mmu-miR-106b-3p:MIMAT0004582	5.350584	6.904035042	7.254206	1.860246
RB_p_mrmir0000001411	mmu-miR-106b-5p:MIMAT0000386	4.967718	5.366413132	6.585205	7.733826
RB_p_mrmir000000336	mmu-miR-107-3p:MIMAT0000647	7.34466	6.206211092	6.915824	6.747674
RB_p_mrmir000000368	mmu-miR-107-5p:MIMAT0017048	5.380206	4.481058643	6.92361	5.528699
RB_p_mrmir000000624	mmu-miR-10a-3p:MIMAT0004659	7.381963	5.803009299	4.597849	2.763715

RB_p_mrmir0000001470	mmu-miR-10a-5p:MIMAT0000648	6.863355	6.379272164	7.453913	2.006066
RB_p_mrmir000000692	mmu-miR-10b-3p:MIMAT0004538	7.208232	2.685264052	6.22217	4.067317
RB_p_mrmir0000001469	mmu-miR-10b-5p:MIMAT0000208	7.393432	7.576972272	8.421685	9.206659
RB_p_mrmir0000001605	mmu-miR-1187:MIMAT0005837	10.61645	8.989251895	11.53945	11.65642
RB_p_mrmir0000001714	mmu-miR-1188-3p:MIMAT0017328	8.616691	7.152665283	8.402246	8.358255
RB_p_mrmir0000002070	mmu-miR-1188-5p:MIMAT0005843	9.227121	6.196101953	8.035423	8.130169
RB_p_mrmir0000001658	mmu-miR-1190:MIMAT0005847	7.06157	6.83073406	7.116918	1.961645
RB_p_mrmir000000738	mmu-miR-1191a:MIMAT0005849	4.560304	3.834560858	7.848509	2.487949
RB_p_mrmir000000301	mmu-miR-1191b-3p:MIMAT0029867	8.291289	4.730116517	6.241614	2.337603
RB_p_mrmir0000001660	mmu-miR-1191b-5p:MIMAT0029866	7.868838	8.56601217	8.523183	8.429945
RB_p_mrmir0000005	mmu-miR-1192:MIMAT0005850	8.447901	7.723180476	8.742815	8.196685
RB_p_mrmir0000001548	mmu-miR-1193-3p:MIMAT0005851	9.063569	4.433689276	1.196549	3.504075
RB_p_mrmir0000002061	mmu-miR-1193-5p:MIMAT0017329	4.208069	5.564962249	6.760495	6.673269
RB_p_mrmir0000001126	mmu-miR-1194:MIMAT0005852	4.735974	6.671501195	6.138656	6.313412
RB_p_mrmir0000001912	mmu-miR-1195:MIMAT0005856	7.967736	3.657882935	7.024634	9.225887
RB_p_mrmir0000001535	mmu-miR-1197-3p:MIMAT0005858	5.944253	5.491031765	7.745425	2.826108
RB_p_mrmir000000952	mmu-miR-1197-5p:MIMAT0017331	6.46403	8.345312803	7.958873	4.996539
RB_p_mrmir00000085	mmu-miR-1198-3p:MIMAT0017332	6.644532	6.649052456	4.201514	3.144787
RB_p_mrmir0000001606	mmu-miR-1198-5p:MIMAT0005859	7.816117	8.039423295	7.268121	6.687419
RB_p_mrmir0000001959	mmu-miR-1199-3p:MIMAT0017333	7.782109	6.708320617	4.68645	7.058177
RB_p_mrmir0000001792	mmu-miR-1199-5p:MIMAT0005860	7.601539	4.617721485	6.574736	4.771758
RB_p_mrmir00000014	mmu-miR-122-3p:MIMAT0017005	7.273315	6.918968859	6.65764	7.304316
RB_p_mrmir000000842	mmu-miR-1224-3p:MIMAT0017231	6.795901	4.406805753	4.357892	5.665935
RB_p_mrmir0000001366	mmu-miR-1224-5p:MIMAT0005460	14.38352	12.6894797	13.74825	13.93445
RB_p_mrmir0000002006	mmu-miR-122-5p:MIMAT0000246	3.873422	5.820134623	6.071866	2.535747
RB_p_mrmir0000001950	mmu-miR-1231-3p:MIMAT0022358	8.322354	6.8816523	5.132988	2.894832
RB_p_mrmir0000001813	mmu-miR-1231-5p:MIMAT0022357	12.94733	12.74927755	12.74838	12.81884
RB_p_mrmir0000001430	mmu-miR-124-3p:MIMAT0000134	8.11037	4.89140629	8.173719	7.568412
RB_p_mrmir000000964	mmu-miR-124-5p:MIMAT0004527	6.839123	5.537431727	3.14239	5.562345
RB_p_mrmir000000934	mmu-miR-1247-3p:MIMAT0014801	10.93679	11.32291381	10.71739	11.5921
RB_p_mrmir000000201	mmu-miR-1247-5p:MIMAT0014800	7.938442	8.981324738	7.28659	4.766995
RB_p_mrmir000000218	mmu-miR-1249-3p:MIMAT0010560	7.82379	1.999919225	7.454928	5.287591
RB_p_mrmir000000382	mmu-miR-1249-5p:MIMAT0014804	12.23351	10.77852069	11.39369	10.97514
RB_p_mrmir000000922	mmu-miR-1251-3p:MIMAT0014825	6.37476	5.285493371	5.99372	4.33813
RB_p_mrmir000000248	mmu-miR-1251-5p:MIMAT0014824	8.452703	2.25697939	4.450392	2.038583
RB_p_mrmir0000002178	mmu-miR-1258-3p:MIMAT0029905	4.373174	4.049924019	7.708278	4.956517
RB_p_mrmir0000001972	mmu-miR-1258-5p:MIMAT0029904	4.272957	4.130172208	7.748313	4.371276
RB_p_mrmir000000181	mmu-miR-125a-3p:MIMAT0004528	10.53923	10.17294371	9.692947	9.703314
RB_p_mrmir0000001705	mmu-miR-125a-5p:MIMAT0000135	7.002502	7.756488291	10.07464	6.747222
RB_p_mrmir000000227	mmu-miR-125b-1-3p:MIMAT0004669	7.366548	4.769568875	7.720801	5.946908
RB_p_mrmir000000161	mmu-miR-125b-2-3p:MIMAT0004529	5.109041	4.715105743	6.37862	1.175441

RB_p_mrmir0000001703	mmu-miR-125b-5p:MIMAT0000136	7.582814	6.080590538	4.961687	6.245685
RB_p_mrmir000000623	mmu-miR-1264-3p:MIMAT0014803	7.359617	4.990817943	4.267561	2.2318
RB_p_mrmir000000443	mmu-miR-1264-5p:MIMAT0014802	5.237322	3.802734756	6.498979	0.949589
RB_p_mrmir0000001752	mmu-miR-126a-3p:MIMAT0000138	3.687935	2.869587542	2.752332	4.254011
RB_p_mrmir000000792	mmu-miR-126a-5p:MIMAT0000137	7.235051	5.596065704	6.005294	3.988716
RB_p_mrmir000000920	mmu-miR-126b-3p:MIMAT0029895	7.168447	7.352793878	6.776737	6.997111
RB_p_mrmir000000592	mmu-miR-126b-5p:MIMAT0029894	5.61697	2.254232074	4.220138	2.885709
RB_p_mrmir0000001743	mmu-miR-127-3p:MIMAT0000139	6.043492	6.076374504	6.48421	4.077614
RB_p_mrmir0000001021	mmu-miR-127-5p:MIMAT0004530	7.89029	4.311982459	5.990874	5.938584
RB_p_mrmir000000940	mmu-miR-128-1-5p:MIMAT0016982	5.455761	5.56278113	4.247737	4.223541
RB_p_mrmir0000001302	mmu-miR-128-2-5p:MIMAT0017069	5.899111	7.216158877	7.878232	4.593068
RB_p_mrmir0000001631	mmu-miR-128-3p:MIMAT0000140	6.792986	2.801698839	3.860628	4.687011
RB_p_mrmir000000574	mmu-miR-1291:MIMAT0031397	4.115011	7.64992517	5.818968	2.980056
RB_p_mrmir00000082	mmu-miR-129-1-3p:MIMAT0016994	7.176138	8.565764486	7.599428	8.016
RB_p_mrmir00000081	mmu-miR-129-2-3p:MIMAT0000544	7.299675	7.756688872	9.076038	7.087182
RB_p_mrmir0000001107	mmu-miR-129-5p:MIMAT0000209	9.530385	7.699332475	7.508681	8.115312
RB_p_mrmir000000776	mmu-miR-1298-3p:MIMAT0014810	5.087838	5.559110727	6.223924	4.716248
RB_p_mrmir0000002201	mmu-miR-1298-5p:MIMAT0014809	4.686687	2.71920339	5.419349	4.713797
RB_p_mrmir000000639	mmu-miR-129b-3p:MIMAT0029863	8.175758	8.251325915	7.677902	7.73427
RB_p_mrmir0000001255	mmu-miR-129b-5p:MIMAT0029862	8.457368	6.465519424	5.665088	7.137375
RB_p_mrmir000000230	mmu-miR-1306-3p:MIMAT0009411	9.119168	8.648938	8.363616	8.65311
RB_p_mrmir000000669	mmu-miR-1306-5p:MIMAT0019136	8.810333	6.917161054	7.002233	6.043409
RB_p_mrmir000000744	mmu-miR-130a-3p:MIMAT0000141	6.717086	8.533684019	6.321463	7.918283
RB_p_mrmir0000001236	mmu-miR-130a-5p:MIMAT0016983	3.901644	3.093845119	6.447505	2.788113
RB_p_mrmir000000742	mmu-miR-130b-3p:MIMAT0000387	8.499381	6.78393807	8.687699	8.363292
RB_p_mrmir000000249	mmu-miR-130b-5p:MIMAT0004583	6.12498	8.94032034	5.210015	3.412506
RB_p_mrmir000000746	mmu-miR-130c:MIMAT0025132	3.24884	6.592421126	6.076837	7.348632
RB_p_mrmir0000001418	mmu-miR-132-3p:MIMAT0000144	6.36897	8.469688786	4.951861	5.04766
RB_p_mrmir00000057	mmu-miR-132-5p:MIMAT0016984	6.958604	2.208589769	5.025551	3.646621
RB_p_mrmir0000002312	mmu-miR-133a-3p:MIMAT0000145	7.328595	9.118581689	6.193326	3.943736
RB_p_mrmir0000001246	mmu-miR-133a-5p:MIMAT0003473	4.574265	6.029651528	6.140546	5.379248
RB_p_mrmir0000002311	mmu-miR-133b-3p:MIMAT0000769	7.708389	3.776833578	7.02734	4.228563
RB_p_mrmir0000001248	mmu-miR-133b-5p:MIMAT0017083	6.791368	6.827819019	6.510678	6.347924
RB_p_mrmir0000002313	mmu-miR-133c:MIMAT0025078	6.947958	5.271722723	5.393666	6.85185
RB_p_mrmir0000001080	mmu-miR-134-3p:MIMAT0016985	4.187262	3.573126645	4.544366	0.914669
RB_p_mrmir0000002118	mmu-miR-134-5p:MIMAT0000146	10.40504	9.796792335	10.81228	9.957518
RB_p_mrmir0000001573	mmu-miR-135a-1-3p:MIMAT0004531	11.20037	9.875247832	9.892183	8.914826
RB_p_mrmir0000002090	mmu-miR-135a-2-3p:MIMAT0017064	7.881802	5.685433917	7.897251	8.427032
RB_p_mrmir0000001589	mmu-miR-135a-5p:MIMAT0000147	5.994362	0.821428676	4.937074	3.180501
RB_p_mrmir000000583	mmu-miR-135b-3p:MIMAT0017044	6.218374	6.482214811	4.487498	2.067906
RB_p_mrmir0000001588	mmu-miR-135b-5p:MIMAT0000612	4.428303	4.81655056	3.537447	0.942471

RB_p_mrmir000000546	mmu-miR-136-3p:MIMAT0004532	7.869174	4.849095062	6.738392	2.125067
RB_p_mrmir000000244	mmu-miR-136-5p:MIMAT0000148	8.555456	1.029485658	7.384634	7.295021
RB_p_mrmir0000002185	mmu-miR-137-3p:MIMAT0000149	5.974159	4.876692638	7.189237	4.544868
RB_p_mrmir000000225	mmu-miR-137-5p:MIMAT0016986	8.187896	7.761747369	6.77948	4.599531
RB_p_mrmir000000932	mmu-miR-138-1-3p:MIMAT0004668	8.573603	7.506317133	7.596987	8.983723
RB_p_mrmir0000001227	mmu-miR-138-2-3p:MIMAT0016987	7.337769	7.850031856	2.170017	6.724128
RB_p_mrmir000000365	mmu-miR-138-5p:MIMAT0000150	7.303171	8.38407653	6.927954	3.663587
RB_p_mrmir0000001997	mmu-miR-139-3p:MIMAT0004662	7.254594	8.224110809	5.948848	4.127429
RB_p_mrmir0000001755	mmu-miR-139-5p:MIMAT0000656	4.758636	3.594702453	5.251833	3.544443
RB_p_mrmir0000001468	mmu-miR-140-3p:MIMAT0000152	9.180465	7.083523708	7.207072	7.574781
RB_p_mrmir000000752	mmu-miR-140-5p:MIMAT0000151	1.284942	3.925829821	6.858574	4.939626
RB_p_mrmir0000001415	mmu-miR-141-3p:MIMAT0000153	6.606555	8.892090911	6.449819	5.932466
RB_p_mrmir000000781	mmu-miR-141-5p:MIMAT0004533	2.211421	2.22894335	6.858667	4.753544
RB_p_mrmir0000002093	mmu-miR-142a-3p:MIMAT0000155	4.627614	5.091717199	7.162027	6.826538
RB_p_mrmir000000758	mmu-miR-142a-5p:MIMAT0000154	3.692783	6.304681292	6.625937	6.23086
RB_p_mrmir0000001694	mmu-miR-142b:MIMAT0031402	5.071284	7.046600394	3.408154	4.154304
RB_p_mrmir0000001877	mmu-miR-143-3p:MIMAT0000247	5.41615	6.520858499	7.174107	6.895784
RB_p_mrmir0000001322	mmu-miR-143-5p:MIMAT0017006	7.33248	4.587756072	7.463256	7.420013
RB_p_mrmir0000001460	mmu-miR-144-3p:MIMAT0000156	5.286046	6.625424389	7.716441	6.171061
RB_p_mrmir0000001267	mmu-miR-144-5p:MIMAT0016988	3.444373	4.93112612	5.637219	2.246259
RB_p_mrmir000000598	mmu-miR-145a-3p:MIMAT0004534	3.143815	7.036132611	5.621582	8.234166
RB_p_mrmir0000001351	mmu-miR-145a-5p:MIMAT0000157	7.780857	8.144379226	9.040987	6.618453
RB_p_mrmir0000001352	mmu-miR-145b:MIMAT0025105	5.213013	5.136100252	7.461013	5.589643
RB_p_mrmir000000891	mmu-miR-146a-3p:MIMAT0016989	6.321458	2.305505754	4.673532	6.38045
RB_p_mrmir0000001874	mmu-miR-146a-5p:MIMAT0000158	6.430424	2.772205091	4.813422	6.584325
RB_p_mrmir0000001205	mmu-miR-146b-3p:MIMAT0004826	7.54615	9.363138117	4.215679	4.135699
RB_p_mrmir0000001872	mmu-miR-146b-5p:MIMAT0003475	7.208475	4.980625213	6.541639	6.760199
RB_p_mrmir0000001397	mmu-miR-147-3p:MIMAT0004857	7.607152	5.856944086	6.873607	3.684517
RB_p_mrmir0000001979	mmu-miR-147-5p:MIMAT0017269	6.613117	4.837010307	4.951353	3.762702
RB_p_mrmir0000001672	mmu-miR-148a-3p:MIMAT0000516	2.331497	4.471370591	6.214688	4.927844
RB_p_mrmir00000032	mmu-miR-148a-5p:MIMAT0004617	4.024537	7.995515731	6.519261	7.067084
RB_p_mrmir0000001673	mmu-miR-148b-3p:MIMAT0000580	8.37922	5.498319244	6.239398	4.007267
RB_p_mrmir0000001123	mmu-miR-148b-5p:MIMAT0017036	4.097258	4.357332346	5.986146	5.196437
RB_p_mrmir0000001156	mmu-miR-149-3p:MIMAT0016990	15.73451	15.93856327	15.19855	15.66617
RB_p_mrmir0000001807	mmu-miR-149-5p:MIMAT0000159	6.473072	6.498327801	7.393866	4.986318
RB_p_mrmir0000001065	mmu-miR-150-3p:MIMAT0004535	10.37185	6.581968718	9.640145	8.910896
RB_p_mrmir0000001769	mmu-miR-150-5p:MIMAT0000160	6.769098	6.176771042	3.686409	3.822866
RB_p_mrmir000000972	mmu-miR-151-3p:MIMAT0000161	7.360581	3.080823649	7.321797	4.322384
RB_p_mrmir0000001739	mmu-miR-151-5p:MIMAT0004536	4.788087	3.876393084	7.324816	7.455382
RB_p_mrmir0000001674	mmu-miR-152-3p:MIMAT0000162	6.049465	5.571276546	7.430699	6.670634
RB_p_mrmir0000001551	mmu-miR-152-5p:MIMAT0016991	6.861994	3.57585827	5.57356	2.197599

RB_p_mrmir0000002238	mmu-miR-153-3p:MIMAT0000163	6.089989	5.910464093	6.280285	2.287505
RB_p_mrmir0000002317	mmu-miR-153-5p:MIMAT0016992	2.244753	6.303772549	6.816521	7.210296
RB_p_mrmir000000128	mmu-miR-154-3p:MIMAT0004537	6.311664	8.274875226	5.449384	1.283927
RB_p_mrmir0000001550	mmu-miR-154-5p:MIMAT0000164	5.954219	6.719196409	6.831523	6.876856
RB_p_mrmir0000001007	mmu-miR-155-3p:MIMAT0016993	5.300055	3.024655128	4.091404	5.101976
RB_p_mrmir0000002168	mmu-miR-155-5p:MIMAT0000165	7.246563	4.807332027	7.910634	9.179203
RB_p_mrmir000000718	mmu-miR-15a-3p:MIMAT0004624	6.158364	5.722387597	6.575797	5.850436
RB_p_mrmir0000001515	mmu-miR-15a-5p:MIMAT0000526	5.827956	3.772092583	4.594538	3.707735
RB_p_mrmir000000904	mmu-miR-15b-3p:MIMAT0004521	6.605094	6.488406554	3.269377	3.16487
RB_p_mrmir0000001516	mmu-miR-15b-5p:MIMAT0000124	5.540278	5.902779974	7.205294	4.615593
RB_p_mrmir000000819	mmu-miR-16-1-3p:MIMAT0004625	6.68851	5.221434983	7.595429	3.979483
RB_p_mrmir000000193	mmu-miR-16-2-3p:MIMAT0017018	4.063355	7.538085287	3.023044	5.335456
RB_p_mrmir0000001517	mmu-miR-16-5p:MIMAT0000527	6.654019	7.163273514	6.301119	4.645017
RB_p_mrmir000000616	mmu-miR-1668:MIMAT0031414	4.297546	4.420927612	7.951763	-0.27081
RB_p_mrmir000000257	mmu-miR-17-3p:MIMAT0000650	7.537294	7.076975054	6.879581	5.324695
RB_p_mrmir000000620	mmu-miR-17-5p:MIMAT0000649	7.887711	4.957842123	6.65069	7.793018
RB_p_mrmir000000200	mmu-miR-181a-1-3p:MIMAT0000660	6.223714	2.263196283	7.779511	4.115955
RB_p_mrmir000000203	mmu-miR-181a-2-3p:MIMAT0005443	7.694036	5.974569736	6.817908	1.557144
RB_p_mrmir00000049	mmu-miR-181a-5p:MIMAT0000210	6.566155	8.747157746	7.692757	7.11477
RB_p_mrmir000000995	mmu-miR-181b-1-3p:MIMAT0017067	6.311507	6.383776384	3.372072	1.91431
RB_p_mrmir000000997	mmu-miR-181b-2-3p:MIMAT0017084	5.519801	2.720627199	4.651188	6.535597
RB_p_mrmir00000050	mmu-miR-181b-5p:MIMAT0000673	10.25297	8.054480645	8.333102	8.712526
RB_p_mrmir000000199	mmu-miR-181c-3p:MIMAT0017068	9.432777	6.121513997	8.093424	8.08056
RB_p_mrmir00000048	mmu-miR-181c-5p:MIMAT0000674	7.254199	7.9631485	1.997911	4.792476
RB_p_mrmir000000831	mmu-miR-181d-3p:MIMAT0017264	7.991859	4.714821197	8.002835	8.891074
RB_p_mrmir00000051	mmu-miR-181d-5p:MIMAT0004324	9.828932	10.38531032	8.381976	7.825166
RB_p_mrmir0000001395	mmu-miR-182-3p:MIMAT0016995	5.855965	8.084465348	0.789252	2.179408
RB_p_mrmir0000002307	mmu-miR-182-5p:MIMAT0000211	7.530345	4.041761701	6.807102	7.145035
RB_p_mrmir0000001362	mmu-miR-183-3p:MIMAT0004539	5.490901	2.197669502	1.650555	3.089998
RB_p_mrmir0000001587	mmu-miR-183-5p:MIMAT0000212	6.03626	5.448650253	6.646461	5.076708
RB_p_mrmir000000300	mmu-miR-1839-3p:MIMAT0009457	6.861408	6.469309072	7.188155	8.810285
RB_p_mrmir000000106	mmu-miR-1839-5p:MIMAT0009456	6.813388	6.723889407	7.685739	3.948443
RB_p_mrmir0000001794	mmu-miR-1843a-3p:MIMAT0014806	6.30397	5.050655589	6.79608	5.970847
RB_p_mrmir0000001586	mmu-miR-1843a-5p:MIMAT0014805	4.634034	3.736891667	7.794464	5.710496
RB_p_mrmir000000858	mmu-miR-1843b-3p:MIMAT0019346	4.100445	6.965900078	8.394832	7.271727
RB_p_mrmir000000571	mmu-miR-1843b-5p:MIMAT0019345	7.161627	1.87821207	6.404851	1.006816
RB_p_mrmir0000001995	mmu-miR-184-3p:MIMAT0000213	7.810687	8.558220884	8.152197	8.452827
RB_p_mrmir000000896	mmu-miR-184-5p:MIMAT0022690	8.17126	6.764249946	7.570163	8.313195
RB_p_mrmir000000434	mmu-miR-185-3p:MIMAT0016996	6.470818	7.999433439	7.625605	6.673421
RB_p_mrmir0000001999	mmu-miR-185-5p:MIMAT0000214	6.045421	6.621380658	7.361665	5.571745
RB_p_mrmir0000001204	mmu-miR-186-3p:MIMAT0004540	5.343987	8.605837314	5.004064	5.855177

RB_p_mrmir000000614	mmu-miR-186-5p:MIMAT0000215	1.888367	6.664650317	5.728106	5.229526
RB_p_mrmir0000001753	mmu-miR-187-3p:MIMAT0000216	7.518819	5.912130169	5.257265	7.021351
RB_p_mrmir000000406	mmu-miR-187-5p:MIMAT0016997	9.047531	7.666991763	8.442817	8.56415
RB_p_mrmir0000001004	mmu-miR-188-3p:MIMAT0004541	3.834014	7.421048648	5.776831	2.885323
RB_p_mrmir000000772	mmu-miR-188-5p:MIMAT0000217	11.60495	11.30243581	12.07176	11.40081
RB_p_mrmir000000610	mmu-miR-1892:MIMAT0007871	13.75641	13.84249646	14.09602	13.69353
RB_p_mrmir0000001277	mmu-miR-1893:MIMAT0007879	8.906229	8.719738887	7.149875	7.896659
RB_p_mrmir0000001177	mmu-miR-1894-3p:MIMAT0007878	13.84503	12.2570356	14.50953	13.95895
RB_p_mrmir0000001017	mmu-miR-1894-5p:MIMAT0007877	8.578515	8.467611306	7.566378	8.047254
RB_p_mrmir000000844	mmu-miR-1895:MIMAT0007867	13.17998	12.70643169	13.5898	11.08896
RB_p_mrmir0000001018	mmu-miR-1896:MIMAT0007873	12.0236	9.332947723	12.08139	11.85029
RB_p_mrmir0000001626	mmu-miR-1897-3p:MIMAT0007865	6.187508	2.987565731	7.462926	4.195278
RB_p_mrmir0000001105	mmu-miR-1897-5p:MIMAT0007864	14.33367	13.46364901	14.97603	14.76263
RB_p_mrmir000000439	mmu-miR-1898:MIMAT0007875	6.915632	7.778499901	8.487857	5.829105
RB_p_mrmir000000349	mmu-miR-1899:MIMAT0007869	6.853448	7.72193058	6.308969	3.37129
RB_p_mrmir000000264	mmu-miR-18a-3p:MIMAT0004626	7.720486	5.88697082	5.147552	6.305413
RB_p_mrmir0000001434	mmu-miR-18a-5p:MIMAT0000528	4.496244	4.907437414	4.414303	7.136402
RB_p_mrmir0000001497	mmu-miR-18b-3p:MIMAT0017270	6.190264	8.32252555	6.4786	5.911469
RB_p_mrmir0000001435	mmu-miR-18b-5p:MIMAT0004858	6.492595	4.830098638	6.370105	6.302167
RB_p_mrmir0000001273	mmu-miR-1900:MIMAT0007870	6.817707	4.424187317	6.972732	6.861094
RB_p_mrmir000000863	mmu-miR-1901:MIMAT0007880	7.067209	7.570080164	7.749858	2.852127
RB_p_mrmir000000322	mmu-miR-1902:MIMAT0007863	4.938811	8.793494002	6.852494	4.733566
RB_p_mrmir000000900	mmu-miR-1903:MIMAT0007868	4.545201	2.240632574	7.612118	3.744364
RB_p_mrmir0000001403	mmu-miR-1904:MIMAT0007874	10.98995	8.194639994	11.09094	10.02501
RB_p_mrmir000000670	mmu-miR-1905:MIMAT0007866	5.618216	6.110891926	7.708242	7.391457
RB_p_mrmir0000001932	mmu-miR-1906:MIMAT0007872	11.07871	10.91886841	11.78726	11.24529
RB_p_mrmir0000001142	mmu-miR-1907:MIMAT0007876	10.83949	9.69431359	11.38627	10.49034
RB_p_mrmir000000234	mmu-miR-190a-3p:MIMAT0016998	6.612771	7.735655302	4.017815	6.595197
RB_p_mrmir0000001915	mmu-miR-190a-5p:MIMAT0000220	3.778786	6.210094662	4.259554	5.37776
RB_p_mrmir000000251	mmu-miR-190b-3p:MIMAT0017267	5.803836	5.667321966	6.660853	0.224506
RB_p_mrmir0000001917	mmu-miR-190b-5p:MIMAT0004852	3.378197	6.935733434	5.600112	2.318084
RB_p_mrmir000000658	mmu-miR-1912-3p:MIMAT0014958	7.187275	7.602475804	7.043645	7.60188
RB_p_mrmir0000001964	mmu-miR-1912-5p:MIMAT0014957	6.825218	5.135860133	4.891178	5.200248
RB_p_mrmir0000001238	mmu-miR-191-3p:MIMAT0004542	7.266546	6.929844719	6.306583	7.24017
RB_p_mrmir000000636	mmu-miR-191-5p:MIMAT0000221	7.209903	5.764154465	6.675891	5.21875
RB_p_mrmir0000001032	mmu-miR-192-3p:MIMAT0017012	6.37915	7.824426483	6.940251	5.647342
RB_p_mrmir0000001024	mmu-miR-192-5p:MIMAT0000517	5.962372	5.227198807	4.827234	5.339341
RB_p_mrmir0000001134	mmu-miR-1927:MIMAT0009390	5.212487	7.610950885	4.482936	3.839803
RB_p_mrmir000000356	mmu-miR-1928:MIMAT0009391	4.44432	3.229734766	4.573327	4.541975
RB_p_mrmir000000706	mmu-miR-1929-3p:MIMAT0022729	7.423656	8.274342012	8.359351	5.725857
RB_p_mrmir0000002218	mmu-miR-1929-5p:MIMAT0009392	9.952958	8.413072043	10.93373	10.38201

RB_p_mrmir0000001323	mmu-miR-1930-3p:MIMAT0017340	9.055055	7.888011018	8.584901	8.408253
RB_p_mrmir000000208	mmu-miR-1930-5p:MIMAT0009393	5.685089	3.902502531	7.289057	2.703636
RB_p_mrmir000000565	mmu-miR-1931:MIMAT0009394	12.19256	11.17418589	10.80928	11.4304
RB_p_mrmir0000001407	mmu-miR-1932:MIMAT0009395	8.344658	7.504276752	6.857202	3.447559
RB_p_mrmir000000817	mmu-miR-1933-3p:MIMAT0009397	8.647312	5.075914141	8.281644	7.716621
RB_p_mrmir000000473	mmu-miR-1933-5p:MIMAT0009396	7.086591	5.859036558	5.590036	5.937718
RB_p_mrmir000000386	mmu-miR-1934-3p:MIMAT0017341	13.09968	12.70925319	11.94535	12.01233
RB_p_mrmir0000001817	mmu-miR-1934-5p:MIMAT0009398	7.721686	7.923232824	7.215475	5.648773
RB_p_mrmir0000001426	mmu-miR-1936:MIMAT0009400	5.960927	6.229866188	5.94259	5.597972
RB_p_mrmir000000950	mmu-miR-1938:MIMAT0009402	8.097823	5.048506263	6.020447	4.065053
RB_p_mrmir00000070	mmu-miR-193a-3p:MIMAT0000223	4.22108	7.17795028	5.811233	3.349631
RB_p_mrmir0000002058	mmu-miR-193a-5p:MIMAT0004544	5.892759	4.809373275	6.807847	6.38235
RB_p_mrmir00000069	mmu-miR-193b-3p:MIMAT0004859	7.2959	6.687921683	7.927797	7.074164
RB_p_mrmir000000942	mmu-miR-193b-5p:MIMAT0017271	10.24368	8.712738322	9.818619	9.823797
RB_p_mrmir000000780	mmu-miR-1941-3p:MIMAT0009406	3.640817	8.476344731	4.728215	4.655906
RB_p_mrmir000000821	mmu-miR-194-1-3p:MIMAT0016999	7.400764	3.10730682	6.233623	4.193297
RB_p_mrmir000000418	mmu-miR-1941-5p:MIMAT0009405	9.505947	7.879557922	8.210656	8.939266
RB_p_mrmir0000001651	mmu-miR-1942:MIMAT0009407	3.911259	7.879335915	6.126051	3.800833
RB_p_mrmir000000823	mmu-miR-194-2-3p:MIMAT0017073	7.105655	6.242781908	6.536957	3.651864
RB_p_mrmir000000734	mmu-miR-1943-3p:MIMAT0017342	7.113841	7.438438073	6.68031	8.552744
RB_p_mrmir000000101	mmu-miR-1943-5p:MIMAT0009408	8.357462	6.657704666	8.296889	7.174041
RB_p_mrmir0000001826	mmu-miR-1945:MIMAT0009410	9.561382	9.444493888	7.517004	9.05918
RB_p_mrmir0000002084	mmu-miR-194-5p:MIMAT0000224	7.359125	8.645103853	7.242704	4.243185
RB_p_mrmir000000346	mmu-miR-1946a:MIMAT0009412	8.75968	5.09185675	7.844853	9.290507
RB_p_mrmir0000001207	mmu-miR-1946b:MIMAT0009443	9.362755	6.141945447	8.58335	7.963401
RB_p_mrmir0000001184	mmu-miR-1947-3p:MIMAT0017343	8.522398	8.198797205	7.182693	5.772828
RB_p_mrmir000000374	mmu-miR-1947-5p:MIMAT0009413	7.736021	5.529302018	5.59597	4.14212
RB_p_mrmir0000002282	mmu-miR-1948-3p:MIMAT0009415	6.463404	4.29923562	6.074633	2.3294
RB_p_mrmir000000536	mmu-miR-1948-5p:MIMAT0017344	5.481372	1.063121577	4.817151	7.908746
RB_p_mrmir000000983	mmu-miR-1949:MIMAT0009416	5.299879	6.246167488	2.408231	3.547465
RB_p_mrmir0000001798	mmu-miR-1950:MIMAT0009417	4.70175	8.242113785	6.847035	3.30524
RB_p_mrmir0000001345	mmu-miR-1951:MIMAT0009422	9.538572	8.638894299	8.169325	9.030111
RB_p_mrmir0000001766	mmu-miR-1952:MIMAT0009423	4.670908	3.985694803	6.094558	3.256171
RB_p_mrmir0000002033	mmu-miR-1953:MIMAT0009424	6.877496	5.4835491	7.342852	5.077614
RB_p_mrmir000000255	mmu-miR-1954:MIMAT0009425	4.952346	4.20902673	7.074334	7.564276
RB_p_mrmir0000001144	mmu-miR-1955-3p:MIMAT0017348	6.007924	8.226861291	3.083935	2.230048
RB_p_mrmir000000475	mmu-miR-1955-5p:MIMAT0009426	5.418407	3.697833022	6.1436	2.36059
RB_p_mrmir000000474	mmu-miR-1956:MIMAT0009428	7.959951	8.167137008	8.468632	4.321324
RB_p_mrmir000000751	mmu-miR-1957a:MIMAT0009430	5.83027	7.743308917	5.602453	4.52643
RB_p_mrmir0000001990	mmu-miR-1957b:MIMAT0025145	6.679637	3.924342321	6.793741	1.722433
RB_p_mrmir0000001533	mmu-miR-1958:MIMAT0009431	6.107807	7.994991581	9.233147	8.634274

RB_p_mrmir000000803	mmu-miR-195a-3p:MIMAT0017000	6.484065	6.700866581	7.522328	5.832584
RB_p_mrmir0000001513	mmu-miR-195a-5p:MIMAT0000225	5.931038	8.23555524	6.050421	4.305163
RB_p_mrmir0000001512	mmu-miR-195b:MIMAT0025076	7.930672	7.437563129	7.435145	6.838796
RB_p_mrmir000000820	mmu-miR-1960:MIMAT0009433	10.64527	10.4241258	12.50227	11.74304
RB_p_mrmir0000001902	mmu-miR-1961:MIMAT0009434	4.168224	5.026111156	2.91158	4.756764
RB_p_mrmir000000311	mmu-miR-1962:MIMAT0009435	8.151839	5.009446895	7.970221	6.865789
RB_p_mrmir0000002037	mmu-miR-1963:MIMAT0009436	7.869578	2.541668594	4.473057	3.995038
RB_p_mrmir000000855	mmu-miR-1964-3p:MIMAT0009437	8.717016	5.031748741	7.38061	1.571426
RB_p_mrmir000000363	mmu-miR-1964-5p:MIMAT0017349	8.567901	8.260618987	8.400996	7.496727
RB_p_mrmir0000002295	mmu-miR-1966-3p:MIMAT0022952	6.664069	7.481116795	5.752545	2.200302
RB_p_mrmir000000100	mmu-miR-1966-5p:MIMAT0009439	12.49434	11.0380063	12.62689	12.35281
RB_p_mrmir0000001887	mmu-miR-1967:MIMAT0009440	11.35842	11.32801036	12.38628	11.77667
RB_p_mrmir000000195	mmu-miR-1968-3p:MIMAT0017350	7.322647	3.277655895	5.172706	-0.33099
RB_p_mrmir0000001935	mmu-miR-1968-5p:MIMAT0009441	6.707533	7.104809351	8.475058	9.015496
RB_p_mrmir00000079	mmu-miR-1969:MIMAT0009442	5.885232	5.107881851	7.526735	4.644094
RB_p_mrmir000000635	mmu-miR-196a-1-3p:MIMAT0017013	8.792981	8.628885468	6.678133	8.04414
RB_p_mrmir0000001744	mmu-miR-196a-2-3p:MIMAT0004618	7.430471	5.814808763	7.070824	6.380478
RB_p_mrmir0000001545	mmu-miR-196a-5p:MIMAT0000518	8.016256	4.831606291	8.461246	4.6666
RB_p_mrmir0000001737	mmu-miR-196b-3p:MIMAT0017170	8.129431	7.954035407	6.316749	6.992211
RB_p_mrmir0000001546	mmu-miR-196b-5p:MIMAT0001081	8.351062	5.410871432	7.691846	5.079366
RB_p_mrmir0000002141	mmu-miR-1970:MIMAT0009444	7.700047	7.003403603	7.39772	6.732158
RB_p_mrmir0000001332	mmu-miR-1971:MIMAT0009446	9.256955	8.430569516	8.317724	4.811253
RB_p_mrmir000000774	mmu-miR-1981-3p:MIMAT0017351	7.433208	4.268778216	7.188994	1.104257
RB_p_mrmir0000001333	mmu-miR-1981-5p:MIMAT0009458	8.097947	7.342230437	8.878458	7.808528
RB_p_mrmir0000001761	mmu-miR-1982-3p:MIMAT0009460	7.598451	7.335064094	7.189369	5.026938
RB_p_mrmir0000002258	mmu-miR-1982-5p:MIMAT0009459	10.12253	8.644029418	10.99618	9.66557
RB_p_mrmir000000994	mmu-miR-1983:MIMAT0009455	7.474459	4.339957729	5.139475	6.251861
RB_p_mrmir000000183	mmu-miR-199a-3p:MIMAT0000230;mmu-miR-199b-3p:MIMAT0004667	5.000817	5.087679749	7.418181	3.795944
RB_p_mrmir000000838	mmu-miR-199a-5p:MIMAT0000229	6.910465	7.599522131	7.610264	3.415729
RB_p_mrmir000000839	mmu-miR-199b-5p:MIMAT0000672	4.397265	4.913022488	6.300144	4.029487
RB_p_mrmir0000002123	mmu-miR-19a-3p:MIMAT0000651	7.502918	5.106620599	7.596883	4.012558
RB_p_mrmir0000001559	mmu-miR-19a-5p:MIMAT0004660	7.40602	4.905137275	7.880928	5.703755
RB_p_mrmir000000503	mmu-miR-19b-1-5p:MIMAT0017065	5.688074	5.963633519	6.963548	-0.49183
RB_p_mrmir000000501	mmu-miR-19b-2-5p:MIMAT0017010	5.657592	1.829300323	6.539289	3.649011
RB_p_mrmir0000002122	mmu-miR-19b-3p:MIMAT0000513	6.85956	7.132559824	7.019025	7.519645
RB_p_mrmir000000187	mmu-miR-1a-1-5p:MIMAT0016979	6.916853	5.180101727	5.188369	2.686975
RB_p_mrmir000000188	mmu-miR-1a-2-5p:MIMAT0017047	5.842782	6.45903154	6.92435	2.765856
RB_p_mrmir0000001987	mmu-miR-1a-3p:MIMAT0000123	5.524519	4.717325233	6.040674	6.523496
RB_p_mrmir0000002057	mmu-miR-1b-3p:MIMAT0017326	5.704803	7.073965674	5.919951	6.249826
RB_p_mrmir0000001464	mmu-miR-1b-5p:MIMAT0005835	4.73358	3.063903653	5.624433	2.086025
RB_p_mrmir0000001416	mmu-miR-200a-3p:MIMAT0000519	4.250622	8.553170122	7.249358	2.868478

RB_p_mrmir000000778	mmu-miR-200a-5p:MIMAT0004619	6.886199	4.294952498	4.699445	5.495776
RB_p_mrmir0000001445	mmu-miR-200b-3p:MIMAT0000233	4.316386	6.721537018	6.904172	5.890045
RB_p_mrmir000000779	mmu-miR-200b-5p:MIMAT0004545	6.373041	7.690572234	6.604416	2.183005
RB_p_mrmir0000001444	mmu-miR-200c-3p:MIMAT0000657	8.793796	5.466756311	4.996062	3.677788
RB_p_mrmir000000958	mmu-miR-200c-5p:MIMAT0004663	7.766253	8.722745986	8.09267	1.863876
RB_p_mrmir0000001839	mmu-miR-201-3p:MIMAT0017001	5.854159	5.681080275	7.086173	4.736058
RB_p_mrmir0000001486	mmu-miR-201-5p:MIMAT0000234	5.368097	5.342070539	6.726079	0.836358
RB_p_mrmir000000321	mmu-miR-202-3p:MIMAT0000235	8.4221	9.454164628	8.644524	8.279391
RB_p_mrmir0000002211	mmu-miR-202-5p:MIMAT0004546	3.128275	5.82792449	3.99953	-0.2827
RB_p_mrmir0000001360	mmu-miR-203-3p:MIMAT0000236	4.898503	5.734142165	8.333371	6.268677
RB_p_mrmir000000488	mmu-miR-203-5p:MIMAT0004547	6.329517	5.672746725	8.46914	0.097613
RB_p_mrmir0000001243	mmu-miR-204-3p:MIMAT0017002	13.90522	12.68365473	13.02669	13.36946
RB_p_mrmir0000002205	mmu-miR-204-5p:MIMAT0000237	3.428043	4.108117436	4.385445	5.395273
RB_p_mrmir0000001171	mmu-miR-205-3p:MIMAT0017003	7.30534	6.414349478	7.114531	7.335441
RB_p_mrmir0000001733	mmu-miR-205-5p:MIMAT0000238	5.476899	3.580400372	4.984594	4.3171
RB_p_mrmir0000001991	mmu-miR-206-3p:MIMAT0000239	10.43157	8.253931826	11.01404	10.49021
RB_p_mrmir000000191	mmu-miR-206-5p:MIMAT0017004	2.573854	2.827804328	7.267319	0.242527
RB_p_mrmir0000001252	mmu-miR-207:MIMAT0000240	4.80583	7.746064344	7.568059	4.699025
RB_p_mrmir000000510	mmu-miR-208a-3p:MIMAT0000520	7.601706	4.781151664	6.563958	5.124653
RB_p_mrmir0000001150	mmu-miR-208a-5p:MIMAT0017014	5.453851	4.327325929	6.824696	1.92225
RB_p_mrmir000000508	mmu-miR-208b-3p:MIMAT0004939	6.028273	7.254519245	7.716815	6.054293
RB_p_mrmir00000090	mmu-miR-208b-5p:MIMAT0017280	4.748103	3.257650439	4.331511	1.668964
RB_p_mrmir000000261	mmu-miR-20a-3p:MIMAT0004627	5.949114	5.662822174	4.408078	4.773308
RB_p_mrmir0000001413	mmu-miR-20a-5p:MIMAT0000529	7.880182	4.638754486	6.968812	8.704701
RB_p_mrmir000000259	mmu-miR-20b-3p:MIMAT0004788	5.593216	4.679463694	6.262722	5.111105
RB_p_mrmir000000618	mmu-miR-20b-5p:MIMAT0003187	7.438241	6.841557901	7.311741	5.454042
RB_p_mrmir0000001078	mmu-miR-210-3p:MIMAT0000658	8.020472	7.700626055	4.768513	6.299073
RB_p_mrmir000000342	mmu-miR-210-5p:MIMAT0017052	8.500956	7.760786172	6.735809	7.384054
RB_p_mrmir0000001175	mmu-miR-211-3p:MIMAT0017059	14.94474	15.11883148	14.22663	14.42588
RB_p_mrmir0000002206	mmu-miR-211-5p:MIMAT0000668	5.926668	5.400196701	6.200607	4.055153
RB_p_mrmir0000001419	mmu-miR-212-3p:MIMAT0000659	7.237458	8.118194333	7.185464	6.568927
RB_p_mrmir000000215	mmu-miR-212-5p:MIMAT0017053	6.113047	4.594174782	8.489284	3.311895
RB_p_mrmir0000001063	mmu-miR-2136:MIMAT0011212	7.277996	6.810055006	7.810605	9.161524
RB_p_mrmir0000001206	mmu-miR-2137:MIMAT0011213	12.85669	13.62387594	13.26559	13.34389
RB_p_mrmir000000362	mmu-miR-2139:MIMAT0011215	7.824205	5.939914302	8.155118	4.140959
RB_p_mrmir000000175	mmu-miR-214-3p:MIMAT0000661	11.5286	10.75229803	10.41373	10.12491
RB_p_mrmir0000001954	mmu-miR-214-5p:MIMAT0004664	5.227354	6.342977091	6.746513	1.321213
RB_p_mrmir0000001818	mmu-miR-215-3p:MIMAT0017169	5.163966	2.896438883	6.433518	3.346955
RB_p_mrmir000000562	mmu-miR-215-5p:MIMAT0000904	6.515033	6.737279795	7.3243	4.999182
RB_p_mrmir000000664	mmu-miR-216a-3p:MIMAT0017054	4.495087	4.746378094	6.841858	3.914214
RB_p_mrmir0000001449	mmu-miR-216a-5p:MIMAT0000662	5.752876	5.293708258	8.02603	4.331891

RB_p_mrmir000000171	mmu-miR-216b-3p:MIMAT0017233	7.062233	3.892243438	4.569177	0.707953
RB_p_mrmir00000036	mmu-miR-216b-5p:MIMAT0003729	3.97963	5.300403159	1.840804	2.922382
RB_p_mrmir0000002306	mmu-miR-216c-3p:MIMAT0029889	6.299579	7.401301585	7.634453	6.337655
RB_p_mrmir0000001119	mmu-miR-216c-5p:MIMAT0029888	4.687329	6.801824251	4.977477	3.570002
RB_p_mrmir000000769	mmu-miR-217-3p:MIMAT0017072	8.166795	6.325755216	6.93992	3.287438
RB_p_mrmir0000001496	mmu-miR-217-5p:MIMAT0000679	5.205068	8.974130125	7.276876	5.316012
RB_p_mrmir00000010	mmu-miR-218-1-3p:MIMAT0004665	7.704699	8.746095047	6.361832	4.427837
RB_p_mrmir000000790	mmu-miR-218-2-3p:MIMAT0005444	7.639323	5.239270931	6.089708	5.462757
RB_p_mrmir0000002229	mmu-miR-2183:MIMAT0011287	7.572705	6.099544146	7.685272	6.320748
RB_p_mrmir0000002277	mmu-miR-218-5p:MIMAT0000663	7.485369	5.573702063	5.213205	-0.34415
RB_p_mrmir000000327	mmu-miR-219a-1-3p:MIMAT0017055	6.655782	4.74210373	6.560647	1.733748
RB_p_mrmir000000295	mmu-miR-219a-2-3p:MIMAT0022841	6.412141	7.221398928	6.817173	4.668071
RB_p_mrmir0000001927	mmu-miR-219a-5p:MIMAT0000664	6.59862	4.754924391	4.972818	2.031929
RB_p_mrmir000000294	mmu-miR-219b-3p:MIMAT0029807	4.11134	9.43537219	5.891269	2.502224
RB_p_mrmir000000331	mmu-miR-219b-5p:MIMAT0029806	8.856506	7.781286047	7.422937	5.21608
RB_p_mrmir000000907	mmu-miR-219c-3p:MIMAT0029893	4.888156	7.338210346	7.558409	1.855565
RB_p_mrmir0000001256	mmu-miR-219c-5p:MIMAT0029892	8.158385	6.725048753	5.527192	3.041677
RB_p_mrmir000000628	mmu-miR-21a-3p:MIMAT0004628	7.394077	6.495675568	7.870864	7.449572
RB_p_mrmir0000001529	mmu-miR-21a-5p:MIMAT0000530	4.216797	6.086053064	5.558073	5.821447
RB_p_mrmir0000001558	mmu-miR-21b:MIMAT0025121	7.188277	4.716023473	4.749385	1.786042
RB_p_mrmir0000001530	mmu-miR-21c:MIMAT0025148	1.899704	6.421408126	7.318309	4.830348
RB_p_mrmir000000357	mmu-miR-221-3p:MIMAT0000669	7.140834	5.918526109	7.22526	5.293468
RB_p_mrmir000000212	mmu-miR-221-5p:MIMAT0017060	5.618039	7.80641338	7.089905	4.510807
RB_p_mrmir000000355	mmu-miR-222-3p:MIMAT0000670	7.179344	5.555652986	7.125033	9.364403
RB_p_mrmir0000001666	mmu-miR-222-5p:MIMAT0017061	6.730959	3.106805068	5.613426	5.085198
RB_p_mrmir0000002098	mmu-miR-223-3p:MIMAT0000665	8.059393	6.936439209	5.519619	7.538739
RB_p_mrmir000000963	mmu-miR-223-5p:MIMAT0017056	2.893608	7.600061565	5.633829	5.445879
RB_p_mrmir00000086	mmu-miR-22-3p:MIMAT0000531	5.368533	4.435485745	7.036821	2.746569
RB_p_mrmir00000037	mmu-miR-224-3p:MIMAT0017062	4.815841	1.547876937	6.692112	5.049507
RB_p_mrmir0000001436	mmu-miR-224-5p:MIMAT0000671	5.674804	6.295136621	6.359091	3.969973
RB_p_mrmir000000495	mmu-miR-22-5p:MIMAT0004629	3.616171	2.941504869	5.163171	5.769369
RB_p_mrmir000000542	mmu-miR-23a-3p:MIMAT0000532	5.836246	3.510072863	7.350047	6.289272
RB_p_mrmir0000001308	mmu-miR-23a-5p:MIMAT0017019	9.077341	5.341150759	8.901954	8.217877
RB_p_mrmir000000541	mmu-miR-23b-3p:MIMAT0000125	4.762053	8.386905085	5.097782	5.237106
RB_p_mrmir0000001311	mmu-miR-23b-5p:MIMAT0016980	2.432011	4.271305355	6.731573	1.830936
RB_p_mrmir0000001382	mmu-miR-24-1-5p:MIMAT0000218	3.792362	5.849971429	8.000497	1.846693
RB_p_mrmir0000001380	mmu-miR-24-2-5p:MIMAT0005440	4.115306	3.278691865	7.804453	4.938585
RB_p_mrmir0000002025	mmu-miR-24-3p:MIMAT0000219	7.525992	7.644798758	7.762626	8.401456
RB_p_mrmir000000794	mmu-miR-25-3p:MIMAT0000652	6.753582	5.381499665	7.178531	1.120652
RB_p_mrmir000000403	mmu-miR-25-5p:MIMAT0017049	7.91607	7.59721018	6.526312	5.231876
RB_p_mrmir000000873	mmu-miR-26a-1-3p:MIMAT0017020	4.29828	8.569055249	6.358552	2.97682

RB_p_mrmir000000895	mmu-miR-26a-2-3p:MIMAT0017058	3.683149	2.016009163	6.791028	4.850281
RB_p_mrmir0000002186	mmu-miR-26a-5p:MIMAT0000533	5.956227	3.615796058	6.500199	4.010829
RB_p_mrmir000000893	mmu-miR-26b-3p:MIMAT0004630	7.552452	5.467448681	6.046641	3.592082
RB_p_mrmir0000002187	mmu-miR-26b-5p:MIMAT0000534	5.351227	3.897582941	6.781743	7.951641
RB_p_mrmir0000002192	mmu-miR-27a-3p:MIMAT0000537	5.860259	7.892765424	6.980238	5.122191
RB_p_mrmir000000427	mmu-miR-27a-5p:MIMAT0004633	7.249869	6.692643239	6.631583	4.539825
RB_p_mrmir0000002193	mmu-miR-27b-3p:MIMAT0000126	6.595532	6.145271313	6.891856	5.081917
RB_p_mrmir000000307	mmu-miR-27b-5p:MIMAT0004522	5.140408	6.996997143	4.185699	5.644673
RB_p_mrmir0000001296	mmu-miR-2861:MIMAT0013803	11.15748	9.408397207	12.46153	12.78907
RB_p_mrmir000000678	mmu-miR-28a-3p:MIMAT0004661	6.439396	7.174379774	5.569535	3.664396
RB_p_mrmir00000094	mmu-miR-28a-5p:MIMAT0000653	6.029278	3.991468837	4.731964	4.203255
RB_p_mrmir000000377	mmu-miR-28b:MIMAT0019354	4.211194	2.784980332	5.405265	4.713975
RB_p_mrmir000000378	mmu-miR-28c:MIMAT0019339	6.807799	5.608935789	6.623107	-0.36063
RB_p_mrmir00000026	mmu-miR-290a-3p:MIMAT0004572	7.155217	4.580531904	7.140339	7.192683
RB_p_mrmir000000237	mmu-miR-290a-5p:MIMAT0000366	7.075868	8.870442542	7.394385	6.172562
RB_p_mrmir000000113	mmu-miR-290b-3p:MIMAT0029901	6.657903	3.554617492	6.857453	5.825247
RB_p_mrmir0000001250	mmu-miR-290b-5p:MIMAT0029900	8.036243	7.913637958	6.480983	2.319343
RB_p_mrmir00000028	mmu-miR-291a-3p:MIMAT0000368	4.602444	0.64247868	5.97227	4.522348
RB_p_mrmir000000768	mmu-miR-291a-5p:MIMAT0000367	5.566095	5.78094156	3.775213	5.6945
RB_p_mrmir00000023	mmu-miR-291b-3p:MIMAT0003190	5.3226	6.594192891	6.480958	5.462962
RB_p_mrmir0000001166	mmu-miR-291b-5p:MIMAT0003189	5.765909	5.919192046	7.755018	6.408927
RB_p_mrmir00000025	mmu-miR-292a-3p:MIMAT0000370	7.258006	4.067281266	8.53532	6.205608
RB_p_mrmir000000238	mmu-miR-292a-5p:MIMAT0000369	8.283216	6.729983685	7.01182	5.525839
RB_p_mrmir00000077	mmu-miR-292b-3p:MIMAT0029865	6.720528	8.206517163	6.613343	7.604671
RB_p_mrmir000000235	mmu-miR-292b-5p:MIMAT0029864	7.722537	2.139981476	3.925518	4.552342
RB_p_mrmir000000478	mmu-miR-293-3p:MIMAT0000371	7.36316	5.752575349	7.539859	7.114603
RB_p_mrmir000000240	mmu-miR-293-5p:MIMAT0004573	2.045431	3.05397207	7.039213	5.589615
RB_p_mrmir00000030	mmu-miR-294-3p:MIMAT0000372	8.650027	8.665443957	7.689844	4.883543
RB_p_mrmir000000236	mmu-miR-294-5p:MIMAT0004574	5.265682	5.171173096	4.969638	7.029253
RB_p_mrmir00000027	mmu-miR-295-3p:MIMAT0000373	6.881046	2.438011642	5.820132	6.407517
RB_p_mrmir000000241	mmu-miR-295-5p:MIMAT0004575	7.562924	6.779855295	5.652096	6.135107
RB_p_mrmir0000001158	mmu-miR-296-3p:MIMAT0004576	7.868091	7.295149284	6.956134	8.741423
RB_p_mrmir000000421	mmu-miR-296-5p:MIMAT0000374	7.364767	8.042085291	8.262155	5.869186
RB_p_mrmir000000589	mmu-miR-297a-5p:MIMAT0000375	9.158538	8.41082589	9.275888	9.701273
RB_p_mrmir0000001565	mmu-miR-297b-3p:MIMAT0004827;mmu-miR-297a-3p:MIMAT0004864;mmu-miR-297c-3p:MIMAT0004866	9.096385	7.500721498	7.644861	9.304795
RB_p_mrmir000000586	mmu-miR-297b-5p:MIMAT0003480	3.339164	4.790737342	6.733523	8.090257
RB_p_mrmir000000587	mmu-miR-297c-5p:MIMAT0004865	6.517516	6.474124309	4.955966	8.571394
RB_p_mrmir0000001152	mmu-miR-298-3p:MIMAT0017007	5.672576	3.03250812	5.26655	-0.39844
RB_p_mrmir0000001271	mmu-miR-298-5p:MIMAT0000376	9.075557	6.981177006	7.708314	9.525943
RB_p_mrmir0000001601	mmu-miR-299a-3p:MIMAT0004577	6.055465	4.215658909	1.59452	4.709047

RB_p_mrmir0000002076	mmu-miR-299a-5p:MIMAT0000377	7.09452	7.083311082	6.646652	7.595207
RB_p_mrmir0000001599	mmu-miR-299b-3p:MIMAT0022837	7.972457	7.763940353	8.054418	7.967839
RB_p_mrmir0000001330	mmu-miR-299b-5p:MIMAT0022836	7.75962	6.528008813	7.240781	7.879471
RB_p_mrmir0000001507	mmu-miR-29a-3p:MIMAT0000535	9.048155	7.656956356	7.769321	5.148737
RB_p_mrmir000000252	mmu-miR-29a-5p:MIMAT0004631	5.374116	3.747960855	3.80598	4.665447
RB_p_mrmir0000001249	mmu-miR-29b-1-5p:MIMAT0004523	2.702744	5.535367635	6.121023	5.759569
RB_p_mrmir0000001068	mmu-miR-29b-2-5p:MIMAT0017063	5.422179	2.429177204	7.134407	3.950335
RB_p_mrmir0000001508	mmu-miR-29b-3p:MIMAT0000127	7.893574	3.505178572	4.741307	4.054759
RB_p_mrmir0000001509	mmu-miR-29c-3p:MIMAT0000536	7.189335	7.970220963	5.468492	3.891398
RB_p_mrmir0000001857	mmu-miR-29c-5p:MIMAT0004632	7.185455	7.331250127	8.162721	7.282142
RB_p_mrmir0000001582	mmu-miR-300-3p:MIMAT0000378	7.10374	4.115279078	7.125405	3.217524
RB_p_mrmir0000002231	mmu-miR-300-5p:MIMAT0004578	4.39037	7.618305381	6.465205	3.44116
RB_p_mrmir000000740	mmu-miR-301a-3p:MIMAT0000379	4.443627	5.727799166	6.132348	3.739901
RB_p_mrmir0000001233	mmu-miR-301a-5p:MIMAT0017008	3.993216	2.82855354	6.474438	2.585668
RB_p_mrmir000000743	mmu-miR-301b-3p:MIMAT0004186	5.701572	3.411220262	7.077472	5.180472
RB_p_mrmir0000001231	mmu-miR-301b-5p:MIMAT0017232	7.125125	5.105084447	6.818361	0.627022
RB_p_mrmir0000001442	mmu-miR-302a-3p:MIMAT0000380	7.37852	2.967620282	3.880141	8.618332
RB_p_mrmir000000283	mmu-miR-302a-5p:MIMAT0004579	7.52458	8.334108617	7.907017	2.915514
RB_p_mrmir0000001441	mmu-miR-302b-3p:MIMAT0003374	6.194617	4.857458095	4.536719	3.633962
RB_p_mrmir000000290	mmu-miR-302b-5p:MIMAT0003373	6.09329	5.089535282	5.537688	2.350183
RB_p_mrmir000000116	mmu-miR-302c-3p:MIMAT0003376	5.436714	2.670797341	7.38111	7.625035
RB_p_mrmir0000001253	mmu-miR-302c-5p:MIMAT0003375	2.545488	2.498677517	4.131463	1.566736
RB_p_mrmir0000001440	mmu-miR-302d-3p:MIMAT0003377	9.211579	3.279358472	4.240588	6.367315
RB_p_mrmir000000289	mmu-miR-302d-5p:MIMAT0017225	8.153879	5.803870721	7.643039	2.641364
RB_p_mrmir0000001696	mmu-miR-3057-3p:MIMAT0014823	7.563694	7.226647483	7.243791	8.950759
RB_p_mrmir000000602	mmu-miR-3057-5p:MIMAT0014822	4.777028	6.119471001	8.312413	7.140227
RB_p_mrmir0000002214	mmu-miR-3058-3p:MIMAT0014814	8.260648	6.461502985	6.795579	6.466426
RB_p_mrmir0000001654	mmu-miR-3058-5p:MIMAT0014813	4.343024	6.706982509	7.702124	5.60758
RB_p_mrmir000000877	mmu-miR-3059-3p:MIMAT0014812	10.95448	9.742575156	11.80045	10.86418
RB_p_mrmir0000002290	mmu-miR-3059-5p:MIMAT0014811	6.361308	7.646578483	7.18302	8.459233
RB_p_mrmir000000826	mmu-miR-3060-3p:MIMAT0014827	8.592009	7.667313424	7.076337	8.396785
RB_p_mrmir0000001290	mmu-miR-3060-5p:MIMAT0014826	4.624884	4.69241723	4.552277	3.370887
RB_p_mrmir000000971	mmu-miR-3061-3p:MIMAT0014829	7.367314	6.785499212	5.919011	4.854644
RB_p_mrmir000000749	mmu-miR-3061-5p:MIMAT0014828	9.347653	8.5981995	8.581429	8.551697
RB_p_mrmir000000936	mmu-miR-3062-3p:MIMAT0014831	7.475171	8.798702197	6.097799	8.468987
RB_p_mrmir0000001259	mmu-miR-3062-5p:MIMAT0014830	4.854148	6.851144773	5.77147	5.938407
RB_p_mrmir0000001886	mmu-miR-3063-3p:MIMAT0014833	6.192997	4.815749837	7.135485	4.877404
RB_p_mrmir0000002002	mmu-miR-3063-5p:MIMAT0014832	4.715232	5.783051966	4.036336	4.736082
RB_p_mrmir0000001942	mmu-miR-3064-3p:MIMAT0014835	7.662219	1.706507223	8.0938	8.404784
RB_p_mrmir0000001809	mmu-miR-3064-5p:MIMAT0014834	8.520054	7.437539034	8.001626	8.212418
RB_p_mrmir0000001652	mmu-miR-3065-3p:MIMAT0014837	7.350062	3.445366258	6.446321	5.327189

RB_p_mrmir0000001625	mmu-miR-3065-5p:MIMAT0014836	6.639145	6.373126852	6.613629	5.562565
RB_p_mrmir000000686	mmu-miR-3066-3p:MIMAT0014839	7.154799	6.806435892	6.16033	7.829429
RB_p_mrmir0000002269	mmu-miR-3066-5p:MIMAT0014838	0.820973	9.013568011	7.372997	3.360768
RB_p_mrmir000000799	mmu-miR-3067-3p:MIMAT0014841	11.01274	9.748519948	11.49191	10.60545
RB_p_mrmir000000494	mmu-miR-3067-5p:MIMAT0014840	7.409409	5.332332673	6.705611	6.367924
RB_p_mrmir0000001320	mmu-miR-3068-3p:MIMAT0014843	6.793856	6.324501618	7.088926	2.381827
RB_p_mrmir0000002250	mmu-miR-3068-5p:MIMAT0014842	5.867671	2.963102451	6.391352	1.34888
RB_p_mrmir0000002246	mmu-miR-3069-3p:MIMAT0014845	5.936663	3.510640657	8.245994	5.758203
RB_p_mrmir0000002253	mmu-miR-3069-5p:MIMAT0014844	4.537764	6.62152407	6.434525	3.547064
RB_p_mrmir0000002068	mmu-miR-3070-2-3p:MIMAT0014849	9.752908	6.866412589	7.314749	8.950989
RB_p_mrmir0000002067	mmu-miR-3070-3p:MIMAT0014847	6.318686	7.702843644	7.968393	5.364671
RB_p_mrmir000000344	mmu-miR-3070-5p:MIMAT0014846	7.658197	7.471614323	7.695583	4.561313
RB_p_mrmir000000545	mmu-miR-3071-3p:MIMAT0014851	6.716054	9.906318595	7.717725	4.965002
RB_p_mrmir000000243	mmu-miR-3071-5p:MIMAT0014850	6.614202	6.837356378	8.133031	1.480931
RB_p_mrmir0000001946	mmu-miR-3072-3p:MIMAT0014853	5.418457	5.617134287	9.113449	4.397345
RB_p_mrmir000000411	mmu-miR-3072-5p:MIMAT0014852	13.16832	13.73668273	13.40487	14.16927
RB_p_mrmir0000002236	mmu-miR-3073a-3p:MIMAT0014855	2.654996	1.31052062	4.676883	1.468934
RB_p_mrmir0000001394	mmu-miR-3073a-5p:MIMAT0014854	4.355127	8.080503399	6.700747	5.661424
RB_p_mrmir0000001051	mmu-miR-3073b-3p:MIMAT0022376	6.115229	3.533861891	7.287489	3.991428
RB_p_mrmir000000579	mmu-miR-3073b-5p:MIMAT0022375	6.290049	6.389086291	7.40482	6.638383
RB_p_mrmir0000001165	mmu-miR-3074-1-3p:MIMAT0014857	6.521017	4.956141921	7.239594	5.995556
RB_p_mrmir0000002160	mmu-miR-3074-2-3p:MIMAT0014946	5.100923	2.487800207	5.668921	6.831148
RB_p_mrmir0000001402	mmu-miR-3074-5p:MIMAT0014856	7.336266	3.650665101	8.432905	1.690676
RB_p_mrmir000000551	mmu-miR-3075-3p:MIMAT0014859	4.978723	5.532962857	4.939718	0.369698
RB_p_mrmir0000002111	mmu-miR-3075-5p:MIMAT0014858	10.10161	8.171916088	10.2799	9.719597
RB_p_mrmir000000913	mmu-miR-3076-3p:MIMAT0014861	7.550242	4.369773762	7.044327	4.12411
RB_p_mrmir000000662	mmu-miR-3076-5p:MIMAT0014860	9.13115	8.626663289	8.744352	8.322979
RB_p_mrmir0000001026	mmu-miR-3077-3p:MIMAT0014863	6.776888	6.89597614	6.584063	4.847386
RB_p_mrmir0000001040	mmu-miR-3077-5p:MIMAT0014862	13.89429	14.7000979	14.71684	14.77085
RB_p_mrmir0000002244	mmu-miR-3078-3p:MIMAT0014865	5.858559	8.284430083	6.549079	5.414667
RB_p_mrmir000000615	mmu-miR-3078-5p:MIMAT0014864	6.185797	5.237633407	6.145228	5.978265
RB_p_mrmir000000721	mmu-miR-3079-3p:MIMAT0014867	5.366563	7.497571376	6.374649	8.698919
RB_p_mrmir0000002301	mmu-miR-3079-5p:MIMAT0014866	5.07478	5.035628674	4.917928	4.314095
RB_p_mrmir0000001724	mmu-miR-3080-3p:MIMAT0014869	6.37539	6.422290673	6.497803	4.792908
RB_p_mrmir0000001845	mmu-miR-3080-5p:MIMAT0014868	7.228558	5.720503429	2.837344	0.136088
RB_p_mrmir0000002242	mmu-miR-3081-3p:MIMAT0014871	4.823766	4.289192923	5.751506	1.611378
RB_p_mrmir0000001139	mmu-miR-3081-5p:MIMAT0014870	11.89397	12.39443102	11.94454	11.74632
RB_p_mrmir000000665	mmu-miR-3082-3p:MIMAT0014873	5.072885	7.326079985	7.632475	4.698512
RB_p_mrmir0000001133	mmu-miR-3082-5p:MIMAT0014872	10.68282	9.517283398	11.29069	11.41463
RB_p_mrmir0000001712	mmu-miR-3083-3p:MIMAT0014875	6.851793	3.121072842	5.095379	3.600473
RB_p_mrmir000000410	mmu-miR-3083-5p:MIMAT0014874	5.644143	4.53738859	4.170153	6.320168

RB_p_mrmir0000002221	mmu-miR-3084-3p:MIMAT0014877	7.289596	4.998717732	4.607159	4.863428
RB_p_mrmir0000001406	mmu-miR-3084-5p:MIMAT0014876	8.216342	7.3195114	6.594136	4.349348
RB_p_mrmir0000001808	mmu-miR-3085-3p:MIMAT0014879	8.582857	6.009594666	5.773618	5.384418
RB_p_mrmir000000446	mmu-miR-3085-5p:MIMAT0014878	8.628941	7.36797901	8.813721	8.384073
RB_p_mrmir000000830	mmu-miR-3086-3p:MIMAT0014881	5.448195	9.944238823	2.35467	6.024934
RB_p_mrmir0000001504	mmu-miR-3086-5p:MIMAT0014880	4.957021	3.66438662	6.157888	5.326878
RB_p_mrmir0000001423	mmu-miR-3087-3p:MIMAT0014896	4.733018	3.619017307	6.715986	2.057751
RB_p_mrmir000000728	mmu-miR-3087-5p:MIMAT0014895	9.75057	8.743367837	10.95499	9.985374
RB_p_mrmir0000002200	mmu-miR-3088-3p:MIMAT0014898	6.532414	8.283050864	8.248958	8.375986
RB_p_mrmir0000001225	mmu-miR-3088-5p:MIMAT0014897	4.8667	5.990895788	4.980335	1.208486
RB_p_mrmir000000338	mmu-miR-3089-3p:MIMAT0014900	7.183011	7.052921907	7.495541	4.279282
RB_p_mrmir0000001911	mmu-miR-3089-5p:MIMAT0014899	8.250362	6.222089959	6.683394	6.334472
RB_p_mrmir0000001697	mmu-miR-3090-3p:MIMAT0014902	6.206357	7.462982318	6.548383	7.298313
RB_p_mrmir0000001358	mmu-miR-3090-5p:MIMAT0014901	8.205264	9.204077315	8.383249	8.276103
RB_p_mrmir000000935	mmu-miR-3091-3p:MIMAT0014904	6.80218	7.483607376	5.13225	4.497498
RB_p_mrmir000000788	mmu-miR-3091-5p:MIMAT0014903	7.871868	6.263426063	6.670532	4.802461
RB_p_mrmir0000001127	mmu-miR-3092-3p:MIMAT0014906	6.357985	3.113429086	6.244887	4.229151
RB_p_mrmir000000428	mmu-miR-3092-5p:MIMAT0014905	4.698209	6.730064741	4.023969	4.035947
RB_p_mrmir0000002129	mmu-miR-3093-3p:MIMAT0014908	10.16271	8.372366225	9.045667	8.983295
RB_p_mrmir000000912	mmu-miR-3093-5p:MIMAT0014907	7.769516	8.126207165	6.059701	5.986838
RB_p_mrmir000000902	mmu-miR-3094-3p:MIMAT0014910	6.957959	8.108634354	5.953612	6.420402
RB_p_mrmir0000002158	mmu-miR-3094-5p:MIMAT0014909	6.155731	6.099119398	4.648969	5.177419
RB_p_mrmir0000001994	mmu-miR-3095-3p:MIMAT0014912	10.85554	7.366696738	10.55753	10.10632
RB_p_mrmir00000088	mmu-miR-3095-5p:MIMAT0014911	8.093234	7.088411412	6.637041	1.272199
RB_p_mrmir0000001000	mmu-miR-3097-3p:MIMAT0014916	3.239858	5.100272095	4.356983	2.785622
RB_p_mrmir000000663	mmu-miR-3097-5p:MIMAT0014915	10.07823	10.25661487	9.841622	9.811542
RB_p_mrmir0000002223	mmu-miR-3098-3p:MIMAT0014918	7.081031	1.094001049	6.621943	5.499705
RB_p_mrmir0000001723	mmu-miR-3098-5p:MIMAT0014917	12.41293	12.49324255	13.54808	13.52012
RB_p_mrmir0000001540	mmu-miR-3099-3p:MIMAT0014816	10.82499	6.750813601	11.09666	9.774855
RB_p_mrmir000000815	mmu-miR-3099-5p:MIMAT0014815	4.969147	5.757242762	8.544329	7.812985
RB_p_mrmir0000001104	mmu-miR-30a-3p:MIMAT0000129	6.994427	5.099812809	9.072073	7.764959
RB_p_mrmir0000002081	mmu-miR-30a-5p:MIMAT0000128	7.282975	7.692820017	3.540591	1.639101
RB_p_mrmir0000001059	mmu-miR-30b-3p:MIMAT0004524	7.121368	7.633747934	6.618087	7.697264
RB_p_mrmir0000002079	mmu-miR-30b-5p:MIMAT0000130	7.474628	7.262840467	6.025695	7.392188
RB_p_mrmir0000001057	mmu-miR-30c-1-3p:MIMAT0004616	7.86966	7.671440872	7.587778	7.40042
RB_p_mrmir0000001056	mmu-miR-30c-2-3p:MIMAT0005438	8.394917	7.962067191	6.940495	5.032261
RB_p_mrmir0000002080	mmu-miR-30c-5p:MIMAT0000514	7.120406	6.125383405	6.110842	5.036925
RB_p_mrmir0000001102	mmu-miR-30d-3p:MIMAT0017011	7.794429	6.572974199	6.669072	6.148257
RB_p_mrmir0000002078	mmu-miR-30d-5p:MIMAT0000515	7.735657	8.737038808	8.322319	5.397192
RB_p_mrmir0000001103	mmu-miR-30e-3p:MIMAT0000249	5.878828	8.229756489	6.234658	8.678688
RB_p_mrmir0000002082	mmu-miR-30e-5p:MIMAT0000248	7.374431	5.788736316	5.904455	4.322303

RB_p_mrmir0000001331	mmu-miR-30f:MIMAT0025179	6.627771	6.513940244	6.750196	7.071085
RB_p_mrmir0000001076	mmu-miR-3100-3p:MIMAT0014920	7.203814	8.301684777	7.181834	9.166224
RB_p_mrmir0000002257	mmu-miR-3100-5p:MIMAT0014919	9.516019	6.798508673	8.31047	7.874154
RB_p_mrmir0000001532	mmu-miR-3101-3p:MIMAT0014922	7.205249	3.995733142	6.258158	8.485643
RB_p_mrmir0000001313	mmu-miR-3101-5p:MIMAT0014921	5.444353	9.825135696	5.973628	5.1832
RB_p_mrmir0000001014	mmu-miR-3102-3p.2-3p:MIMAT0014935	7.559089	1.635311279	6.231088	7.405002
RB_p_mrmir0000001141	mmu-miR-3102-3p:MIMAT0014936	8.270315	7.15153974	7.819345	5.619968
RB_p_mrmir0000001328	mmu-miR-3102-5p.2-5p:MIMAT0014934	12.50802	12.72637343	13.6019	14.25122
RB_p_mrmir0000001375	mmu-miR-3102-5p:MIMAT0014933	13.15161	13.35998267	14.57691	14.20724
RB_p_mrmir0000001421	mmu-miR-3103-3p:MIMAT0014938	6.145052	8.324480002	8.025623	6.153221
RB_p_mrmir0000001263	mmu-miR-3103-5p:MIMAT0014937	7.410808	7.836382001	6.949271	7.647691
RB_p_mrmir000000220	mmu-miR-3104-3p:MIMAT0014940	7.66082	8.324976122	7.151237	7.141964
RB_p_mrmir0000001543	mmu-miR-3104-5p:MIMAT0014939	10.06053	7.914352266	9.125271	8.468242
RB_p_mrmir000000269	mmu-miR-3105-3p:MIMAT0014942	5.168559	9.567685901	4.703064	5.016675
RB_p_mrmir000000305	mmu-miR-3105-5p:MIMAT0014941	7.347397	5.58233299	6.602276	7.467926
RB_p_mrmir0000001239	mmu-miR-3106-3p:MIMAT0014818	6.551422	5.487404895	7.815794	4.752712
RB_p_mrmir0000002027	mmu-miR-3106-5p:MIMAT0014817	3.951406	3.049861921	6.244927	5.181367
RB_p_mrmir000000955	mmu-miR-3108-3p:MIMAT0014948	6.948919	-0.27225331	5.609524	5.710462
RB_p_mrmir0000001357	mmu-miR-3108-5p:MIMAT0014947	6.954491	1.933100634	8.090593	-0.2202
RB_p_mrmir0000001542	mmu-miR-3109-3p:MIMAT0014950	5.202392	4.385329059	4.40983	3.991438
RB_p_mrmir000000142	mmu-miR-3109-5p:MIMAT0014949	7.827369	6.409422261	9.120324	5.420775
RB_p_mrmir0000001182	mmu-miR-3110-3p:MIMAT0014952	9.049261	8.072695599	8.86943	8.91264
RB_p_mrmir0000002222	mmu-miR-3110-5p:MIMAT0014951	7.095466	8.144092056	7.12969	5.378776
RB_p_mrmir000000757	mmu-miR-3112-3p:MIMAT0014956	4.56881	3.168479865	6.815598	5.278151
RB_p_mrmir000000189	mmu-miR-3112-5p:MIMAT0014955	6.219093	7.721589872	7.917865	5.009623
RB_p_mrmir0000001727	mmu-miR-3113-3p:MIMAT0014960	3.341776	3.804717823	5.575382	7.033969
RB_p_mrmir0000001353	mmu-miR-3113-5p:MIMAT0014959	6.917008	1.183825857	4.665255	6.374748
RB_p_mrmir0000001962	mmu-miR-31-3p:MIMAT0004634	6.240883	3.750209486	5.627688	2.617468
RB_p_mrmir000000689	mmu-miR-3154:MIMAT0035714	14.32213	14.7049385	14.9434	14.99442
RB_p_mrmir000000387	mmu-miR-31-5p:MIMAT0000538	6.389218	3.478507049	8.371853	7.46447
RB_p_mrmir0000003	mmu-miR-320-3p:MIMAT0000666	10.83767	12.17076504	10.51392	9.732816
RB_p_mrmir0000001211	mmu-miR-320-5p:MIMAT0017057	7.248489	3.074520181	8.412759	4.867559
RB_p_mrmir0000008	mmu-miR-322-3p:MIMAT0000549	6.920463	7.686017822	7.197426	7.762035
RB_p_mrmir000000698	mmu-miR-322-5p:MIMAT0000548	4.360883	6.35693033	7.0246	5.879537
RB_p_mrmir000000667	mmu-miR-323-3p:MIMAT0000551	5.035027	4.629312904	7.788294	3.752132
RB_p_mrmir000000449	mmu-miR-323-5p:MIMAT0004638	7.812822	5.212564773	6.43177	4.899136
RB_p_mrmir000000653	mmu-miR-32-3p:MIMAT0017050	5.759266	7.040747517	9.977758	9.776525
RB_p_mrmir000000811	mmu-miR-324-3p:MIMAT0000556	7.409167	7.454124532	6.717928	4.57393
RB_p_mrmir000000915	mmu-miR-324-5p:MIMAT0000555	7.733854	5.738783181	8.22427	6.442116
RB_p_mrmir0000002285	mmu-miR-325-3p:MIMAT0004640	5.799213	6.381135666	6.168523	5.805545

RB_p_mrmir000000871	mmu-miR-325-5p:MIMAT0000558	5.699592	7.295740335	4.751326	6.661253
RB_p_mrmir0000001612	mmu-miR-32-5p:MIMAT0000654	0.286916	0.774356224	5.638667	6.245254
RB_p_mrmir000000878	mmu-miR-326-3p:MIMAT0000559	4.372217	5.603392749	4.96498	5.059661
RB_p_mrmir0000001300	mmu-miR-326-5p:MIMAT0017027	8.657713	7.22284424	8.629777	8.943237
RB_p_mrmir000000285	mmu-miR-327:MIMAT0004867	10.89483	8.208881041	9.946077	10.90958
RB_p_mrmir0000001049	mmu-miR-328-3p:MIMAT0000565	8.266869	9.009648896	6.253042	5.90555
RB_p_mrmir0000001305	mmu-miR-328-5p:MIMAT0017030	14.45015	14.14911916	14.184	14.1271
RB_p_mrmir00000040	mmu-miR-329-3p:MIMAT0000567	7.713981	7.865755649	7.091756	6.659916
RB_p_mrmir000000324	mmu-miR-329-5p:MIMAT0017032	8.054344	6.449084497	7.470477	6.61677
RB_p_mrmir0000001173	mmu-miR-330-3p:MIMAT0000569	6.868258	8.053111602	7.14769	8.613714
RB_p_mrmir0000001782	mmu-miR-330-5p:MIMAT0004642	7.456273	3.08597757	8.660457	6.038558
RB_p_mrmir0000001202	mmu-miR-331-3p:MIMAT0000571	6.988792	4.396688001	7.313224	3.762775
RB_p_mrmir000000974	mmu-miR-331-5p:MIMAT0004643	8.563009	2.466033279	6.548811	2.300168
RB_p_mrmir000000651	mmu-miR-33-3p:MIMAT0004666	7.770823	6.569770896	4.590388	6.78524
RB_p_mrmir0000002324	mmu-miR-335-3p:MIMAT0004704	6.194781	7.204018665	7.194336	4.972871
RB_p_mrmir0000001627	mmu-miR-335-5p:MIMAT0000766	5.791947	5.246051162	2.602684	6.467334
RB_p_mrmir0000001379	mmu-miR-33-5p:MIMAT0000667	1.921719	7.591218668	3.506151	6.491159
RB_p_mrmir0000001657	mmu-miR-337-3p:MIMAT0000578	5.924972	1.456757169	5.621365	2.786747
RB_p_mrmir000000931	mmu-miR-337-5p:MIMAT0004644	7.420876	6.749227059	4.352282	4.264618
RB_p_mrmir0000001691	mmu-miR-338-3p:MIMAT0000582	4.532121	5.930888951	6.605747	5.962835
RB_p_mrmir00000039	mmu-miR-338-5p:MIMAT0004647	7.038476	5.131097533	6.929111	6.581883
RB_p_mrmir0000001884	mmu-miR-339-3p:MIMAT0004649	6.157999	5.465531446	6.941934	6.583435
RB_p_mrmir0000001711	mmu-miR-339-5p:MIMAT0000584	7.216414	3.838180182	6.752324	6.959165
RB_p_mrmir0000001720	mmu-miR-340-3p:MIMAT0000586	7.932728	0.874486435	3.196656	2.434752
RB_p_mrmir0000002179	mmu-miR-340-5p:MIMAT0004651	7.431812	4.834052825	5.771	0.757018
RB_p_mrmir0000001749	mmu-miR-341-3p:MIMAT0000588	8.50317	8.205374367	7.147348	7.805793
RB_p_mrmir000000944	mmu-miR-341-5p:MIMAT0017037	5.838379	2.495773869	5.249736	3.024716
RB_p_mrmir0000001760	mmu-miR-342-3p:MIMAT0000590	9.210357	7.980519814	7.015532	8.673511
RB_p_mrmir000000436	mmu-miR-342-5p:MIMAT0004653	4.669403	3.95412976	4.680132	4.771172
RB_p_mrmir0000001773	mmu-miR-343:MIMAT0004868	5.468952	6.460819505	1.935431	6.82621
RB_p_mrmir0000001920	mmu-miR-344-3p:MIMAT0000593	6.615321	3.786850626	6.411086	6.620744
RB_p_mrmir000000467	mmu-miR-344-5p:MIMAT0017038	8.34686	8.093123378	6.374394	3.752308
RB_p_mrmir000000795	mmu-miR-344b-3p:MIMAT0014926	5.167891	7.393304859	6.877265	3.487376
RB_p_mrmir000000466	mmu-miR-344b-5p:MIMAT0014925	5.444454	5.123088011	7.65638	5.232439
RB_p_mrmir0000001921	mmu-miR-344c-3p:MIMAT0014928	5.920806	6.349466781	4.637952	5.292511
RB_p_mrmir000000468	mmu-miR-344c-5p:MIMAT0014927	7.873636	5.566062257	6.969522	3.398148
RB_p_mrmir000000469	mmu-miR-344d-1-5p:MIMAT0014819	6.223464	5.487372746	6.954716	2.77993
RB_p_mrmir000000476	mmu-miR-344d-2-5p:MIMAT0014961	5.441753	2.332218345	6.942191	6.65979
RB_p_mrmir000000463	mmu-miR-344d-3-5p:MIMAT0014807	6.403174	5.883545898	6.165695	3.719036
RB_p_mrmir0000001164	mmu-miR-344d-3p:MIMAT0014808	6.264133	5.146853411	6.657426	3.957752
RB_p_mrmir0000001163	mmu-miR-344e-3p:MIMAT0014924	7.282084	7.709086625	2.717739	3.424885

RB_p_mrmir000000724	mmu-miR-344e-5p:MIMAT0014923;mmu-miR-344h-5p:MIMAT0022383	7.078881	5.037929099	8.377318	4.154471
RB_p_mrmir0000001015	mmu-miR-344f-3p:MIMAT0014932	6.90562	7.290499328	7.550889	1.42834
RB_p_mrmir000000471	mmu-miR-344f-5p:MIMAT0014931	7.873256	1.382423553	5.858215	7.304741
RB_p_mrmir000000722	mmu-miR-344g-3p:MIMAT0014930	7.678721	2.205861833	5.870915	3.530598
RB_p_mrmir000000464	mmu-miR-344g-5p:MIMAT0014929	6.866738	6.530893222	7.947142	6.423257
RB_p_mrmir0000001316	mmu-miR-344h-3p:MIMAT0022384	7.218286	7.034667302	6.546232	6.686513
RB_p_mrmir000000110	mmu-miR-344i:MIMAT0022503	5.527616	7.012686662	3.338659	5.49567
RB_p_mrmir000000881	mmu-miR-345-3p:MIMAT0004656	9.237467	7.594632557	7.751594	8.25571
RB_p_mrmir0000001237	mmu-miR-345-5p:MIMAT0000595	6.83627	6.034264241	6.587123	3.243998
RB_p_mrmir000000391	mmu-miR-346-3p:MIMAT0017039	11.02325	10.38028183	9.552833	10.04399
RB_p_mrmir0000002109	mmu-miR-346-5p:MIMAT0000597	6.749786	7.195566574	8.801298	7.368545
RB_p_mrmir0000001647	mmu-miR-3470a:MIMAT0015640	7.151342	4.40823932	5.626556	4.727517
RB_p_mrmir0000001644	mmu-miR-3470b:MIMAT0015641	5.413315	0.692324247	7.551597	5.826019
RB_p_mrmir0000001876	mmu-miR-3471:MIMAT0015642	3.837676	4.626830697	6.589164	5.875582
RB_p_mrmir0000001448	mmu-miR-3472:MIMAT0015643	12.58418	12.61235795	13.34639	13.05795
RB_p_mrmir0000002000	mmu-miR-3473a:MIMAT0015645	8.719942	7.714052948	8.540863	8.30712
RB_p_mrmir0000001293	mmu-miR-3473b:MIMAT0020367	10.10278	8.524580735	10.24346	9.112596
RB_p_mrmir0000001779	mmu-miR-3473c:MIMAT0020614	7.735204	7.954461055	7.503389	6.881022
RB_p_mrmir000000810	mmu-miR-3473d:MIMAT0020632	5.623046	5.913426026	8.708771	8.502975
RB_p_mrmir0000001294	mmu-miR-3473e:MIMAT0025587	10.20366	10.18850984	10.57141	10.28812
RB_p_mrmir000000621	mmu-miR-3473f:MIMAT0031390	9.37668	8.958599864	11.13225	10.20255
RB_p_mrmir000000612	mmu-miR-3473g:MIMAT0031427	13.38572	13.44278386	12.85899	12.93693
RB_p_mrmir000000853	mmu-miR-3474:MIMAT0015646	9.352879	7.213328599	9.30962	8.812709
RB_p_mrmir0000001805	mmu-miR-3475-3p:MIMAT0015219	3.704249	6.964593634	6.158838	7.25251
RB_p_mrmir00000035	mmu-miR-3475-5p:MIMAT0026642	7.519332	7.200930067	7.07557	8.004848
RB_p_mrmir000000126	mmu-miR-34a-3p:MIMAT0017022	5.768581	5.893479824	4.26287	3.77349
RB_p_mrmir0000002015	mmu-miR-34a-5p:MIMAT0000542	6.099038	6.938682231	6.948858	4.040332
RB_p_mrmir000000125	mmu-miR-34b-3p:MIMAT0004581	8.303267	6.019328812	7.212331	8.076732
RB_p_mrmir000000395	mmu-miR-34b-5p:MIMAT0000382	5.234502	5.730253027	6.995957	3.844713
RB_p_mrmir000000124	mmu-miR-34c-3p:MIMAT0004580	8.793804	9.27363281	8.784555	10.46053
RB_p_mrmir000000396	mmu-miR-34c-5p:MIMAT0000381	5.585932	7.101593731	8.207092	4.5006
RB_p_mrmir0000002189	mmu-miR-350-3p:MIMAT0000605	7.489393	6.09213261	6.978924	4.832417
RB_p_mrmir00000024	mmu-miR-350-5p:MIMAT0017040	4.566737	3.747655805	6.693253	5.424878
RB_p_mrmir0000001317	mmu-miR-351-3p:MIMAT0017042	8.923346	6.903314987	7.388415	8.845235
RB_p_mrmir0000001706	mmu-miR-351-5p:MIMAT0000609	7.231156	5.261851154	5.931976	4.926813
RB_p_mrmir0000002007	mmu-miR-3535:MIMAT0031410	7.113821	8.831115439	5.87337	7.355325
RB_p_mrmir000000246	mmu-miR-3544-3p:MIMAT0022354	7.97678	4.18588138	6.921081	6.447164
RB_p_mrmir000000291	mmu-miR-3544-5p:MIMAT0022353	5.974012	3.693990883	8.383578	5.081366
RB_p_mrmir0000001879	mmu-miR-3547-3p:MIMAT0027833	7.113493	6.705739195	8.313688	1.974429
RB_p_mrmir0000001387	mmu-miR-3547-5p:MIMAT0027832	13.79433	13.1887123	13.3371	13.28223
RB_p_mrmir000000408	mmu-miR-3552:MIMAT0035715	7.42146	7.958732081	6.416561	3.768838

RB_p_mrmir0000001670	mmu-miR-3569-3p:MIMAT0029855	6.546613	4.389557856	6.076992	6.354069
RB_p_mrmir0000001742	mmu-miR-3569-5p:MIMAT0029854	12.70831	11.75736514	11.79818	11.6833
RB_p_mrmir0000001456	mmu-miR-3572-3p:MIMAT0020636	6.58667	6.715471383	8.261197	4.387996
RB_p_mrmir0000002046	mmu-miR-3572-5p:MIMAT0022986	13.48568	13.12228728	12.95986	13.16871
RB_p_mrmir0000001700	mmu-miR-361-3p:MIMAT0017075	7.866676	7.03735042	7.298995	7.543079
RB_p_mrmir0000002181	mmu-miR-361-5p:MIMAT0000704	7.337351	5.248601973	7.063504	5.836423
RB_p_mrmir000000969	mmu-miR-3618-3p:MIMAT0035717	4.361699	3.566482148	7.096536	5.715446
RB_p_mrmir0000002121	mmu-miR-3618-5p:MIMAT0035716	6.048678	5.250601812	6.953782	2.236686
RB_p_mrmir0000001041	mmu-miR-3620-3p:MIMAT0029879	9.015966	9.130083707	9.152679	7.647133
RB_p_mrmir0000001082	mmu-miR-3620-5p:MIMAT0029878	12.92129	12.56806518	11.81977	10.96807
RB_p_mrmir00000041	mmu-miR-362-3p:MIMAT0004684	6.413198	6.201652617	4.703971	6.178902
RB_p_mrmir000000133	mmu-miR-362-5p:MIMAT0000706	2.253887	5.960024888	2.759714	4.9552
RB_p_mrmir000000150	mmu-miR-363-3p:MIMAT0000708	6.233518	3.041301076	2.654492	1.427908
RB_p_mrmir000000736	mmu-miR-363-5p:MIMAT0017076	7.673056	8.237288886	6.880566	5.912844
RB_p_mrmir000000416	mmu-miR-365-1-5p:MIMAT0017077	5.744682	8.124348027	8.552715	8.047755
RB_p_mrmir000000415	mmu-miR-365-2-5p:MIMAT0017179	7.888546	7.04765438	6.815821	5.246782
RB_p_mrmir0000001450	mmu-miR-365-3p:MIMAT0000711	6.733158	6.060380381	6.202682	7.837205
RB_p_mrmir000000151	mmu-miR-367-3p:MIMAT0003181	7.515847	6.180681787	7.522775	3.420245
RB_p_mrmir000000282	mmu-miR-367-5p:MIMAT0017214	6.398557	2.812701149	4.35679	1.977809
RB_p_mrmir000000121	mmu-miR-369-3p:MIMAT0003186	1.472183	2.610869858	0.759812	5.487734
RB_p_mrmir000000330	mmu-miR-369-5p:MIMAT0003185	6.860618	2.145804035	8.446158	5.360409
RB_p_mrmir0000001210	mmu-miR-370-3p:MIMAT0001095	9.048356	6.73712405	7.551571	8.297465
RB_p_mrmir000000731	mmu-miR-370-5p:MIMAT0017174	7.081069	6.677311871	7.231363	3.217
RB_p_mrmir0000001329	mmu-miR-374b-3p:MIMAT0003728	7.423051	6.390045633	4.89502	6.733174
RB_p_mrmir000000526	mmu-miR-374b-5p:MIMAT0003727	3.643916	4.003188113	6.100745	3.531802
RB_p_mrmir000000284	mmu-miR-374c-3p:MIMAT0014954	4.525398	2.74537404	3.473606	5.876025
RB_p_mrmir000000514	mmu-miR-374c-5p:MIMAT0014953	6.576405	4.224316587	4.950753	1.745878
RB_p_mrmir0000002318	mmu-miR-375-3p:MIMAT0000739	6.657811	7.549006487	5.02656	7.541272
RB_p_mrmir0000001215	mmu-miR-375-5p:MIMAT0017078	8.059109	7.26378557	7.396609	5.480411
RB_p_mrmir000000554	mmu-miR-376a-3p:MIMAT0000740	6.477863	5.334419666	5.181862	6.916179
RB_p_mrmir0000001315	mmu-miR-376a-5p:MIMAT0003387	7.171582	8.267461787	6.215269	3.035948
RB_p_mrmir000000544	mmu-miR-376b-3p:MIMAT0001092	4.48754	4.639443693	5.944677	6.100364
RB_p_mrmir0000001383	mmu-miR-376b-5p:MIMAT0003388	6.766985	5.29914916	6.209458	0.950194
RB_p_mrmir00000044	mmu-miR-376c-3p:MIMAT0003183	6.45527	2.581022977	4.236857	3.008096
RB_p_mrmir0000001385	mmu-miR-376c-5p:MIMAT0005295	5.954725	4.520281478	5.552935	1.121351
RB_p_mrmir000000540	mmu-miR-377-3p:MIMAT0000741	6.372578	7.351856142	5.692579	0.934189
RB_p_mrmir000000323	mmu-miR-377-5p:MIMAT0017079	6.898066	3.366209008	5.607462	0.655044
RB_p_mrmir000000272	mmu-miR-378a-3p:MIMAT0003151	10.08558	9.573358946	10.14074	9.969774
RB_p_mrmir0000001008	mmu-miR-378a-5p:MIMAT0000742	3.353804	2.297699309	6.921025	4.139493
RB_p_mrmir0000001044	mmu-miR-378b:MIMAT0019348	8.359051	7.112655196	8.290401	8.797155
RB_p_mrmir000000271	mmu-miR-378c:MIMAT0025138	8.067803	5.524322581	8.425405	8.091845

RB_p_mrmir000000275	mmu-miR-378d:MIMAT0025167	9.209748	7.768543279	8.996951	9.204037
RB_p_mrmir0000001593	mmu-miR-379-3p:MIMAT0017080	4.609161	3.527251998	3.611997	3.187575
RB_p_mrmir0000002062	mmu-miR-379-5p:MIMAT0000743	7.834861	5.041813922	6.922803	3.585751
RB_p_mrmir0000001595	mmu-miR-380-3p:MIMAT0000745	6.300885	4.583165346	6.665556	3.240734
RB_p_mrmir000000582	mmu-miR-380-5p:MIMAT0000744	6.369689	5.793302526	6.378364	4.785195
RB_p_mrmir0000001563	mmu-miR-381-3p:MIMAT0000746	5.281258	6.388668423	6.139479	6.608526
RB_p_mrmir000000348	mmu-miR-381-5p:MIMAT0017081	5.723599	5.932923613	5.974209	5.760223
RB_p_mrmir0000001686	mmu-miR-382-3p:MIMAT0004691	7.577929	4.574251372	6.763405	2.553843
RB_p_mrmir0000001125	mmu-miR-382-5p:MIMAT0000747	5.723195	2.972180179	6.285293	6.119774
RB_p_mrmir000000806	mmu-miR-383-3p:MIMAT0017082	5.069692	3.372738139	5.995216	3.294086
RB_p_mrmir000000329	mmu-miR-383-5p:MIMAT0000748	7.795438	7.916441508	7.760591	8.333305
RB_p_mrmir000000596	mmu-miR-384-3p:MIMAT0001076	0.701152	3.678360442	4.182606	1.45272
RB_p_mrmir0000002077	mmu-miR-384-5p:MIMAT0004745	5.049973	2.080258336	3.61998	3.108574
RB_p_mrmir0000001278	mmu-miR-3960:MIMAT0019336	15.389	15.6211173	14.35219	15.50882
RB_p_mrmir0000001948	mmu-miR-3961:MIMAT0019338	6.321167	4.127448365	5.172736	3.820051
RB_p_mrmir000000438	mmu-miR-3962:MIMAT0019340	2.341348	1.720286324	5.609715	1.30143
RB_p_mrmir0000002094	mmu-miR-3963:MIMAT0019341	6.914614	9.272610244	5.534002	6.325006
RB_p_mrmir000000511	mmu-miR-3964:MIMAT0019344	4.848264	4.225318797	7.835874	7.888923
RB_p_mrmir0000001977	mmu-miR-3965:MIMAT0019347	6.439244	0.88680135	7.487067	0.205053
RB_p_mrmir000000361	mmu-miR-3966:MIMAT0019350	4.780331	3.675236849	7.656029	8.381422
RB_p_mrmir000000370	mmu-miR-3967:MIMAT0019351	6.453517	2.879034868	6.603003	2.591803
RB_p_mrmir000000905	mmu-miR-3968:MIMAT0019352	8.174537	8.262934917	7.818781	7.291646
RB_p_mrmir000000851	mmu-miR-3969:MIMAT0019353	6.552393	6.290615888	6.000274	3.602071
RB_p_mrmir0000001159	mmu-miR-3970:MIMAT0019355	5.773064	3.718653905	6.149588	1.938594
RB_p_mrmir0000001005	mmu-miR-3971:MIMAT0019356	3.995549	5.841008569	5.665044	7.269072
RB_p_mrmir0000001128	mmu-miR-409-3p:MIMAT0001090	6.559306	5.449640897	5.964706	5.47522
RB_p_mrmir000000451	mmu-miR-409-5p:MIMAT0004746	6.836034	7.315063624	7.769789	5.783771
RB_p_mrmir000000123	mmu-miR-410-3p:MIMAT0001091	4.317485	5.459390176	7.267649	-0.22935
RB_p_mrmir000000459	mmu-miR-410-5p:MIMAT0017172	6.105016	3.321687875	6.555461	6.559239
RB_p_mrmir0000001592	mmu-miR-411-3p:MIMAT0001093	6.813729	6.761732953	6.815297	8.723255
RB_p_mrmir0000001553	mmu-miR-411-5p:MIMAT0004747	6.111452	4.566623777	7.222494	3.812768
RB_p_mrmir0000002195	mmu-miR-412-3p:MIMAT0001094	7.586513	7.761553178	7.317052	4.822267
RB_p_mrmir0000002064	mmu-miR-412-5p:MIMAT0017173	4.466716	6.579976297	7.68618	1.347834
RB_p_mrmir000000539	mmu-miR-421-3p:MIMAT0004869	7.818216	4.299904292	7.879594	4.763026
RB_p_mrmir0000001002	mmu-miR-421-5p:MIMAT0017273	4.897137	4.837875709	8.301706	6.344072
RB_p_mrmir000000360	mmu-miR-423-3p:MIMAT0003454	7.049302	4.107717391	7.091841	4.228148
RB_p_mrmir0000001891	mmu-miR-423-5p:MIMAT0004825	11.44557	10.86421885	10.62552	10.72033
RB_p_mrmir000000553	mmu-miR-425-3p:MIMAT0001342	7.419176	8.024700912	7.59189	6.881013
RB_p_mrmir000000138	mmu-miR-425-5p:MIMAT0004750	7.053327	7.685816876	8.879758	8.648249
RB_p_mrmir0000001447	mmu-miR-429-3p:MIMAT0001537	4.87278	5.857846651	5.98496	2.91666
RB_p_mrmir0000001359	mmu-miR-429-5p:MIMAT0017178	4.146029	4.337250722	5.667633	4.864533

RB_p_mrmir000000733	mmu-miR-431-3p:MIMAT0004753	8.091399	8.091946989	1.380041	5.814329
RB_p_mrmir0000002114	mmu-miR-431-5p:MIMAT0001418	5.85599	3.135738339	4.49229	3.168927
RB_p_mrmir0000001827	mmu-miR-432:MIMAT0012771	11.2059	8.874973861	10.12552	10.1434
RB_p_mrmir000000547	mmu-miR-433-3p:MIMAT0001420	5.746794	6.637804457	6.896385	7.085259
RB_p_mrmir0000001477	mmu-miR-433-5p:MIMAT0001419	7.078538	7.350108768	6.384023	4.20288
RB_p_mrmir0000002298	mmu-miR-434-3p:MIMAT0001422	7.470994	5.75598786	7.786634	8.442584
RB_p_mrmir0000001229	mmu-miR-434-5p:MIMAT0001421	3.854881	4.408542867	4.311471	4.053517
RB_p_mrmir0000002240	mmu-miR-448-3p:MIMAT0001533	5.195741	7.905927998	5.891737	2.498413
RB_p_mrmir0000001117	mmu-miR-448-5p:MIMAT0017176	6.583969	3.866352498	6.556262	4.917544
RB_p_mrmir000000705	mmu-miR-449a-3p:MIMAT0017180	6.399793	4.887166309	7.0859	5.361521
RB_p_mrmir0000002014	mmu-miR-449a-5p:MIMAT0001542	5.527628	6.167446798	5.169932	3.984571
RB_p_mrmir000000397	mmu-miR-449b:MIMAT0005447	4.15877	3.931438489	6.728731	4.846514
RB_p_mrmir000000755	mmu-miR-449c-3p:MIMAT0022715	5.336611	4.990576931	4.229723	6.30116
RB_p_mrmir000000394	mmu-miR-449c-5p:MIMAT0003460	4.922689	9.991488963	6.48338	5.842407
RB_p_mrmir000000603	mmu-miR-450a-1-3p:MIMAT0017182	6.936218	2.00694935	6.336523	6.20989
RB_p_mrmir000000607	mmu-miR-450a-2-3p:MIMAT0004789	5.511999	8.710631188	5.061474	0.785454
RB_p_mrmir0000002322	mmu-miR-450a-5p:MIMAT0001546	4.547533	6.061640274	7.882446	8.340812
RB_p_mrmir000000604	mmu-miR-450b-3p:MIMAT0003512	4.823227	1.054877897	4.294209	1.247752
RB_p_mrmir0000002320	mmu-miR-450b-5p:MIMAT0003511	7.531974	5.003145203	6.722482	3.499484
RB_p_mrmir00000013	mmu-miR-451a:MIMAT0001632	5.303133	7.495174585	6.791366	0.710986
RB_p_mrmir0000002039	mmu-miR-451b:MIMAT0025178	5.90845	8.437752086	9.541812	7.738829
RB_p_mrmir0000001669	mmu-miR-452-3p:MIMAT0017194	6.798375	5.726556737	7.84029	5.995119
RB_p_mrmir0000002162	mmu-miR-452-5p:MIMAT0001637	8.582397	9.073830839	9.416722	9.451954
RB_p_mrmir000000455	mmu-miR-453:MIMAT0004870	4.669175	6.920069772	6.961234	4.948733
RB_p_mrmir0000001192	mmu-miR-455-3p:MIMAT0003742	6.663814	5.901651909	6.887682	7.200534
RB_p_mrmir0000001597	mmu-miR-455-5p:MIMAT0003485	2.556323	5.285458649	5.431175	4.049924
RB_p_mrmir0000001913	mmu-miR-463-3p:MIMAT0004758	0.670106	3.189671338	8.07985	2.971579
RB_p_mrmir0000001473	mmu-miR-463-5p:MIMAT0002104	7.374922	5.262530472	6.488223	0.809268
RB_p_mrmir0000001167	mmu-miR-465a-3p:MIMAT0004217;mmu-miR-465b-3p:MIMAT0004872;mmu-miR-465c-3p:MIMAT0004874	7.445941	5.74785614	7.708105	8.484198
RB_p_mrmir0000001619	mmu-miR-465a-5p:MIMAT0002106	7.575612	5.343713029	7.84876	4.411417
RB_p_mrmir0000001622	mmu-miR-465b-5p:MIMAT0004871	5.702473	4.981829737	7.006077	3.814947
RB_p_mrmir0000001620	mmu-miR-465c-5p:MIMAT0004873	3.028644	2.360574885	4.643587	1.401617
RB_p_mrmir0000001918	mmu-miR-465d-3p:MIMAT0029881	8.251599	9.103128986	5.901481	3.606818
RB_p_mrmir0000001621	mmu-miR-465d-5p:MIMAT0029880	6.576581	9.264934865	6.769972	6.779574
RB_p_mrmir0000001567	mmu-miR-466a-3p:MIMAT0002107;mmu-miR-466e-3p:MIMAT0004880	8.778146	8.094462148	7.690875	9.665307
RB_p_mrmir0000001603	mmu-miR-466a-5p:MIMAT0004759	4.662224	6.065588355	4.93603	7.959583
RB_p_mrmir000000520	mmu-miR-466b-3p:MIMAT0004876;mmu-miR-466c-3p:MIMAT0004878;mmu-miR-466p-3p:MIMAT0014892	7.977708	7.473338187	6.876966	9.51968

RB_p_mrmir0000001922	mmu-miR-466b-5p:MIMAT0004875;mmu-miR-466o-5p:MIMAT0014885	4.31443	5.411445678	5.24857	5.925285
RB_p_mrmir0000001923	mmu-miR-466c-5p:MIMAT0004877	8.433063	7.562414042	9.616615	9.931928
RB_p_mrmir0000001568	mmu-miR-466d-3p:MIMAT0004931	8.614397	7.527635755	8.897564	10.49853
RB_p_mrmir0000002147	mmu-miR-466d-5p:MIMAT0004930	6.388643	4.047495056	7.055367	4.031403
RB_p_mrmir0000001170	mmu-miR-466e-5p:MIMAT0004879	5.842141	3.464126682	5.255953	4.38295
RB_p_mrmir000000229	mmu-miR-466f:MIMAT0005844	9.814489	7.344753808	10.07705	10.65444
RB_p_mrmir000000762	mmu-miR-466f-3p:MIMAT0004882	12.11359	11.53401811	12.88772	13.63051
RB_p_mrmir0000001482	mmu-miR-466f-5p:MIMAT0004881	10.13287	8.607620376	10.59571	10.64743
RB_p_mrmir000000516	mmu-miR-466g:MIMAT0004883	9.917001	9.234413799	8.213737	9.705755
RB_p_mrmir0000001475	mmu-miR-466h-3p:MIMAT0017274	12.10969	11.08976668	12.8749	12.76407
RB_p_mrmir0000002145	mmu-miR-466h-5p:MIMAT0004884	9.152288	8.046665566	9.119724	9.946243
RB_p_mrmir000000515	mmu-miR-466i-3p:MIMAT0005834	10.85468	8.058231717	10.70468	10.13417
RB_p_mrmir0000002149	mmu-miR-466i-5p:MIMAT0017325	11.64733	9.95572701	12.38018	12.24295
RB_p_mrmir0000002143	mmu-miR-466j:MIMAT0005848	9.701597	8.280119583	10.22386	10.4336
RB_p_mrmir0000002148	mmu-miR-466k:MIMAT0005845	5.713555	5.604756264	6.535239	1.452477
RB_p_mrmir0000001560	mmu-miR-466l-3p:MIMAT0005830	7.453507	2.988733206	6.660859	5.80756
RB_p_mrmir0000002280	mmu-miR-466l-5p:MIMAT0017322	2.320911	3.340158437	-0.30665	3.231144
RB_p_mrmir0000001462	mmu-miR-466m-3p:MIMAT0014883	10.54872	10.67837357	11.10971	12.38279
RB_p_mrmir0000001569	mmu-miR-466n-3p:MIMAT0014894	5.799351	7.136912512	7.016902	5.914549
RB_p_mrmir0000001399	mmu-miR-466n-5p:MIMAT0014893	6.941251	5.100726489	7.442923	6.957841
RB_p_mrmir0000001463	mmu-miR-466o-3p:MIMAT0014886	7.91246	6.866929303	7.631032	8.685381
RB_p_mrmir0000001602	mmu-miR-466p-5p:MIMAT0014891	6.479944	1.96283445	4.806557	4.735829
RB_p_mrmir0000001378	mmu-miR-466q:MIMAT0020631	10.82588	9.45412154	11.49853	11.88804
RB_p_mrmir000000767	mmu-miR-467a-3p:MIMAT0002108	9.394424	8.878259611	10.09057	9.79771
RB_p_mrmir0000001437	mmu-miR-467a-5p:MIMAT0003409	5.447465	5.703998435	6.812759	1.571045
RB_p_mrmir000000528	mmu-miR-467b-3p:MIMAT0003478	10.41168	10.74463147	11.02549	12.44963
RB_p_mrmir0000001335	mmu-miR-467b-5p:MIMAT0005448	6.517444	5.477414025	5.414199	4.047771
RB_p_mrmir000000530	mmu-miR-467c-3p:MIMAT0017275	9.760659	8.427052305	10.42231	10.44148
RB_p_mrmir0000001439	mmu-miR-467c-5p:MIMAT0004885	6.665521	5.996120618	6.338221	6.835661
RB_p_mrmir000000529	mmu-miR-467d-3p:MIMAT0004887	11.49033	10.49260823	11.67407	12.98655
RB_p_mrmir0000001438	mmu-miR-467d-5p:MIMAT0004886	4.308944	4.648000206	4.244954	4.195051
RB_p_mrmir000000531	mmu-miR-467e-3p:MIMAT0005294	8.769392	7.783307331	6.699192	8.311844
RB_p_mrmir000000512	mmu-miR-467e-5p:MIMAT0005293	6.072965	4.562369237	3.940116	3.764212
RB_p_mrmir000000527	mmu-miR-467f:MIMAT0005846	11.69161	11.32253544	11.76724	12.69352
RB_p_mrmir0000001564	mmu-miR-467g:MIMAT0005854	8.793864	7.189999202	9.930299	10.68095
RB_p_mrmir000000513	mmu-miR-467h:MIMAT0005855	7.951749	6.117828498	7.49779	5.995754
RB_p_mrmir0000001581	mmu-miR-468-3p:MIMAT0002109	9.757916	8.294839218	10.43535	10.43865
RB_p_mrmir0000001138	mmu-miR-468-5p:MIMAT0022699	5.380052	6.832357033	6.066418	3.512538
RB_p_mrmir00000052	mmu-miR-470-3p:MIMAT0004760	7.920469	3.832142407	8.123481	8.755274
RB_p_mrmir0000002226	mmu-miR-470-5p:MIMAT0002111	9.010374	6.737762035	7.227179	8.775041
RB_p_mrmir0000001836	mmu-miR-471-3p:MIMAT0017195	7.272188	5.77952293	8.658772	1.107685

RB_p_mrmir0000001480	mmu-miR-471-5p:MIMAT0002112	6.022514	3.83877188	5.411949	7.428357
RB_p_mrmir0000001643	mmu-miR-483-3p:MIMAT0003120	6.509323	5.677655283	6.288256	3.395374
RB_p_mrmir00000076	mmu-miR-483-5p:MIMAT0004782	14.96095	15.03196878	15.36918	14.57561
RB_p_mrmir0000001661	mmu-miR-484:MIMAT0003127	7.196468	8.175615578	6.699967	7.523042
RB_p_mrmir000000472	mmu-miR-485-3p:MIMAT0003129	7.734478	5.523291733	6.54488	6.81063
RB_p_mrmir000000312	mmu-miR-485-5p:MIMAT0003128	7.231367	6.703647795	6.068948	5.197691
RB_p_mrmir000000939	mmu-miR-486a-3p:MIMAT0017206	7.286534	4.569130456	5.533856	6.686756
RB_p_mrmir0000001731	mmu-miR-486a-5p:MIMAT0003130;mmu-miR-486b-5p:MIMAT0014943	7.97608	5.305176588	5.639075	6.925236
RB_p_mrmir000000938	mmu-miR-486b-3p:MIMAT0014944	7.677857	10.76151237	5.926463	8.111964
RB_p_mrmir000000136	mmu-miR-487b-3p:MIMAT0003184	7.380639	3.683460014	7.768072	4.591996
RB_p_mrmir0000002071	mmu-miR-487b-5p:MIMAT0017216	7.678288	6.574886903	6.62962	6.454587
RB_p_mrmir0000002228	mmu-miR-488-3p:MIMAT0003450	6.627511	4.900164949	5.686924	6.486079
RB_p_mrmir000000833	mmu-miR-488-5p:MIMAT0003449	5.874674	7.801710181	6.411974	2.853547
RB_p_mrmir000000137	mmu-miR-489-3p:MIMAT0003112	6.511783	3.97736703	5.76476	4.030241
RB_p_mrmir0000002099	mmu-miR-489-5p:MIMAT0022704	3.836608	2.064819745	7.230869	6.024737
RB_p_mrmir000000633	mmu-miR-490-3p:MIMAT0003780	7.866904	7.167442148	5.451906	6.958832
RB_p_mrmir000000827	mmu-miR-490-5p:MIMAT0017261	8.009997	7.601232983	6.857311	6.944849
RB_p_mrmir0000001087	mmu-miR-491-3p:MIMAT0017255	6.710292	4.150903921	8.53018	4.762724
RB_p_mrmir000000483	mmu-miR-491-5p:MIMAT0003486	6.213751	4.095371625	7.974781	8.340343
RB_p_mrmir0000001846	mmu-miR-493-3p:MIMAT0004888	7.385854	8.442178813	7.273699	8.297899
RB_p_mrmir0000002271	mmu-miR-493-5p:MIMAT0017276	3.661	7.809213718	5.686745	5.490754
RB_p_mrmir0000001833	mmu-miR-494-3p:MIMAT0003182	13.32369	14.25753201	12.90556	14.23391
RB_p_mrmir000000458	mmu-miR-494-5p:MIMAT0017215	6.310052	8.867949878	7.605531	8.651222
RB_p_mrmir0000006	mmu-miR-495-3p:MIMAT0003456	6.419299	8.698384851	5.067554	2.405196
RB_p_mrmir0000001124	mmu-miR-495-5p:MIMAT0017249	5.258642	6.090287472	3.917639	6.031401
RB_p_mrmir0000001908	mmu-miR-496a-3p:MIMAT0003738	6.733339	8.638976411	7.812779	6.692554
RB_p_mrmir000000454	mmu-miR-496a-5p:MIMAT0017244	3.801332	4.150453469	6.912324	5.727342
RB_p_mrmir000000630	mmu-miR-496b:MIMAT0025158	5.81236	5.080144337	3.492071	3.838504
RB_p_mrmir000000613	mmu-miR-497a-3p:MIMAT0017247	9.174751	-0.17079151	0.679995	3.98533
RB_p_mrmir000000699	mmu-miR-497a-5p:MIMAT0003453	5.822429	2.979781117	8.778147	4.742407
RB_p_mrmir000000668	mmu-miR-497b:MIMAT0031404	6.060696	5.842374635	7.460571	3.569385
RB_p_mrmir0000001116	mmu-miR-499-3p:MIMAT0017254	5.252963	2.048128567	6.10703	4.627699
RB_p_mrmir0000002165	mmu-miR-499-5p:MIMAT0003482	0.588487	7.190631105	4.210355	5.59702
RB_p_mrmir000000141	mmu-miR-500-3p:MIMAT0003507	8.079463	5.990763628	6.229363	5.6932
RB_p_mrmir000000132	mmu-miR-500-5p:MIMAT0017258	5.719861	2.415716048	7.32477	4.98981
RB_p_mrmir000000139	mmu-miR-501-3p:MIMAT0003509	6.088292	2.451042913	6.173407	0.565426
RB_p_mrmir000000135	mmu-miR-501-5p:MIMAT0003508	6.998461	7.222478741	6.493677	2.075402
RB_p_mrmir0000001160	mmu-miR-503-3p:MIMAT0004790	6.475857	2.708785428	7.431138	4.427433
RB_p_mrmir0000001519	mmu-miR-503-5p:MIMAT0003188	7.10189	5.779566924	7.453425	5.880697
RB_p_mrmir000000417	mmu-miR-504-3p:MIMAT0017277	11.64321	11.42684234	11.71437	12.72565
RB_p_mrmir000000297	mmu-miR-504-5p:MIMAT0004889	7.38999	6.373302387	4.930747	2.788781

RB_p_mrmir000000358	mmu-miR-5046:MIMAT0020540	7.682704	6.570463241	7.240372	5.131199
RB_p_mrmir000000954	mmu-miR-505-3p:MIMAT0003513	7.293777	8.746413674	5.468107	6.111834
RB_p_mrmir0000001288	mmu-miR-505-5p:MIMAT0017259	7.170894	6.398773371	5.523612	6.506699
RB_p_mrmir0000001925	mmu-miR-509-3p:MIMAT0004891	3.007869	7.177014538	2.999102	5.521981
RB_p_mrmir0000001488	mmu-miR-509-5p:MIMAT0004890	6.502192	7.709943633	6.554729	3.216414
RB_p_mrmir0000001401	mmu-miR-5098:MIMAT0020605	2.388762	5.750795548	7.613228	6.794986
RB_p_mrmir0000002175	mmu-miR-5099:MIMAT0020606	8.652442	10.34164358	10.19648	10.85676
RB_p_mrmir0000001736	mmu-miR-5100:MIMAT0020607	7.055606	6.797667512	7.555873	7.816504
RB_p_mrmir0000002319	mmu-miR-5101:MIMAT0020608	6.01597	3.430003986	6.473207	-0.79966
RB_p_mrmir0000001681	mmu-miR-5103:MIMAT0020610	4.970524	4.951599646	5.781476	1.768908
RB_p_mrmir0000001079	mmu-miR-5104:MIMAT0020611	7.700086	7.917174691	7.075874	7.248089
RB_p_mrmir000000445	mmu-miR-5106:MIMAT0020613	7.733143	6.179925285	6.487734	5.292163
RB_p_mrmir000000634	mmu-miR-5107-3p:MIMAT0022985	6.210474	5.873419752	7.152128	7.462579
RB_p_mrmir0000002041	mmu-miR-5107-5p:MIMAT0020615	13.61875	14.14368377	14.4402	13.56239
RB_p_mrmir0000001339	mmu-miR-5108:MIMAT0020616	7.323283	6.491711135	6.951795	6.464855
RB_p_mrmir0000001261	mmu-miR-5110:MIMAT0020618	11.16418	10.35874569	12.75759	12.21406
RB_p_mrmir0000001525	mmu-miR-5112:MIMAT0020620	11.8921	10.66589932	10.97469	9.683917
RB_p_mrmir000000172	mmu-miR-5113:MIMAT0020621	11.02392	9.87615537	11.95012	11.70546
RB_p_mrmir000000147	mmu-miR-511-3p:MIMAT0017281	8.159313	5.108999607	9.82746	9.908431
RB_p_mrmir000000273	mmu-miR-5114:MIMAT0020622	8.818676	7.545424857	9.070241	8.9995
RB_p_mrmir000000568	mmu-miR-511-5p:MIMAT0004940	8.436185	5.022632911	8.013366	2.292659
RB_p_mrmir0000002300	mmu-miR-5116:MIMAT0020624	7.261014	8.908872472	8.041818	3.835558
RB_p_mrmir000000109	mmu-miR-5118:MIMAT0020626	4.405033	7.462817255	6.779792	2.985315
RB_p_mrmir000000775	mmu-miR-5119:MIMAT0020627	11.39619	11.92784374	11.21053	11.38542
RB_p_mrmir0000002310	mmu-miR-5120:MIMAT0020628	8.86874	2.603046347	5.384566	5.728044
RB_p_mrmir000000371	mmu-miR-5121:MIMAT0020629	8.164738	4.769929419	3.939957	5.023119
RB_p_mrmir000000861	mmu-miR-5122:MIMAT0020630	9.617065	8.019847469	7.99395	7.638956
RB_p_mrmir0000002089	mmu-miR-5123:MIMAT0020633	7.512194	6.346504041	6.558984	4.126203
RB_p_mrmir0000001318	mmu-miR-5124a:MIMAT0020634	7.994034	8.042068458	2.378654	5.841355
RB_p_mrmir0000002024	mmu-miR-5124b:MIMAT0025136	7.691963	5.981981126	5.527197	7.818582
RB_p_mrmir0000001799	mmu-miR-5125:MIMAT0020635	5.050295	6.413724334	7.143993	5.377829
RB_p_mrmir0000001219	mmu-miR-5126:MIMAT0020637	15.50838	15.93356229	15.94925	15.68334
RB_p_mrmir0000001770	mmu-miR-5127:MIMAT0020638	4.757314	3.441578126	6.053608	3.828718
RB_p_mrmir000000652	mmu-miR-5128:MIMAT0020639	11.68818	10.55047603	11.91008	11.0531
RB_p_mrmir000000145	mmu-miR-5129-3p:MIMAT0022987	6.642161	1.777831733	9.093964	6.969437
RB_p_mrmir000000590	mmu-miR-5129-5p:MIMAT0020640	8.034093	7.637218127	7.266177	4.420367
RB_p_mrmir0000001045	mmu-miR-5130:MIMAT0020641	14.44676	13.76229572	13.15122	13.23702
RB_p_mrmir000000928	mmu-miR-5131:MIMAT0020642	7.316046	7.079354845	8.660468	7.652492
RB_p_mrmir0000001027	mmu-miR-5132-3p:MIMAT0022988	6.544233	5.601640176	7.267974	7.933051
RB_p_mrmir0000001223	mmu-miR-5132-5p:MIMAT0020643	10.89338	9.424021922	9.86083	10.07854
RB_p_mrmir0000001241	mmu-miR-5133:MIMAT0020644	8.926316	8.007646514	9.067846	7.914282

RB_p_mrmir000000226	mmu-miR-5134-3p:MIMAT0022989	8.070375	3.108890719	6.816811	1.142218
RB_p_mrmir0000002252	mmu-miR-5134-5p:MIMAT0020645	9.418264	9.873794793	9.693665	10.09151
RB_p_mrmir000000444	mmu-miR-5135:MIMAT0020646	10.41258	7.851090139	8.540237	8.971541
RB_p_mrmir000000537	mmu-miR-5136:MIMAT0020647	7.403289	8.232539238	4.504929	6.223483
RB_p_mrmir000000876	mmu-miR-532-3p:MIMAT0004781	7.614052	3.992492119	6.654105	6.707184
RB_p_mrmir000000784	mmu-miR-532-5p:MIMAT0002889	5.188856	4.375562523	8.724466	6.692472
RB_p_mrmir000000760	mmu-miR-539-3p:MIMAT0017208	5.644734	4.101804554	5.890465	7.778143
RB_p_mrmir0000001258	mmu-miR-539-5p:MIMAT0003169	7.144648	6.234228695	7.826562	7.099379
RB_p_mrmir000000441	mmu-miR-540-3p:MIMAT0003167	5.133741	5.578022799	5.347011	3.819638
RB_p_mrmir000000644	mmu-miR-540-5p:MIMAT0004786	5.947531	7.996791096	6.838582	6.150416
RB_p_mrmir0000002018	mmu-miR-541-3p:MIMAT0017209	7.978782	6.467958462	6.384983	7.772133
RB_p_mrmir000000102	mmu-miR-541-5p:MIMAT0003170	3.857183	6.892987761	6.407321	5.853991
RB_p_mrmir0000002117	mmu-miR-542-3p:MIMAT0003172	4.458651	8.340432074	7.641304	3.348456
RB_p_mrmir0000001011	mmu-miR-542-5p:MIMAT0003171	6.43009	4.932013029	6.108639	3.757602
RB_p_mrmir00000012	mmu-miR-543-3p:MIMAT0003168	6.498382	0.698707431	5.43115	5.592527
RB_p_mrmir000000120	mmu-miR-543-5p:MIMAT0017207	6.583629	3.994128549	5.897809	7.629131
RB_p_mrmir000000600	mmu-miR-544-3p:MIMAT0004941	4.960516	7.475749301	5.497343	5.718409
RB_p_mrmir0000001829	mmu-miR-544-5p:MIMAT0017282	8.115761	2.84258502	5.68156	4.834442
RB_p_mrmir000000580	mmu-miR-546:MIMAT0003166	4.892257	8.093579454	6.759649	6.043893
RB_p_mrmir0000001098	mmu-miR-547-3p:MIMAT0003173	6.50492	2.209218388	4.151551	7.235576
RB_p_mrmir000000687	mmu-miR-547-5p:MIMAT0017210	6.37387	5.507941669	2.268096	5.874751
RB_p_mrmir0000001214	mmu-miR-551b-3p:MIMAT0003890	5.968068	5.905816415	4.843461	1.93382
RB_p_mrmir0000001115	mmu-miR-551b-5p:MIMAT0017236	8.034132	7.13513078	7.716542	5.30983
RB_p_mrmir0000002107	mmu-miR-5615-3p:MIMAT0022356	6.868905	6.400973334	7.610346	7.447969
RB_p_mrmir0000001100	mmu-miR-5615-5p:MIMAT0022355	5.991313	5.022672786	8.076803	8.143753
RB_p_mrmir00000074	mmu-miR-5616-3p:MIMAT0022360	10.78048	9.90954879	10.91546	10.79773
RB_p_mrmir0000002289	mmu-miR-5616-5p:MIMAT0022359	5.526633	8.120800096	5.807663	1.091554
RB_p_mrmir000000719	mmu-miR-5617-3p:MIMAT0022362	6.302269	7.820785114	7.006187	7.204789
RB_p_mrmir0000001334	mmu-miR-5617-5p:MIMAT0022361	8.283557	6.385222871	7.928563	6.243593
RB_p_mrmir000000984	mmu-miR-5618-3p:MIMAT0022364	3.248057	4.909848462	5.169315	4.291018
RB_p_mrmir0000001471	mmu-miR-5618-5p:MIMAT0022363	2.363318	3.573940977	6.036435	4.829094
RB_p_mrmir000000903	mmu-miR-5619-3p:MIMAT0022366	4.25917	7.0484635	6.618543	6.625297
RB_p_mrmir000000462	mmu-miR-5619-5p:MIMAT0022365	5.029102	6.219756815	6.13725	-0.7782
RB_p_mrmir000000184	mmu-miR-5620-3p:MIMAT0022368	8.354684	4.422625504	5.624764	7.231815
RB_p_mrmir000000217	mmu-miR-5620-5p:MIMAT0022367	6.282658	8.7028994	8.529821	7.394113
RB_p_mrmir0000002042	mmu-miR-5621-3p:MIMAT0022370	7.336671	6.992786545	6.58762	5.33027
RB_p_mrmir000000383	mmu-miR-5621-5p:MIMAT0022369	4.696408	8.966590741	7.374992	2.844872
RB_p_mrmir0000001086	mmu-miR-5622-3p:MIMAT0022372	11.94232	11.30183824	11.51215	10.11912
RB_p_mrmir0000002194	mmu-miR-5622-5p:MIMAT0022371	7.580238	7.801892272	6.555989	8.261038
RB_p_mrmir0000001404	mmu-miR-5623-3p:MIMAT0022374	5.331368	3.336858973	4.429024	4.078842
RB_p_mrmir0000002013	mmu-miR-5623-5p:MIMAT0022373	6.284941	5.859894774	6.923181	6.513496

RB_p_mrmir0000002167	mmu-miR-5624-3p:MIMAT0022378	3.861332	4.713788226	4.260491	4.399707
RB_p_mrmir0000001616	mmu-miR-5624-5p:MIMAT0022377	6.993596	8.916035515	7.058092	4.300169
RB_p_mrmir0000001028	mmu-miR-5625-3p:MIMAT0022380	7.34515	7.752986179	7.226358	8.59452
RB_p_mrmir000000849	mmu-miR-5625-5p:MIMAT0022379	3.707796	4.410227934	6.746584	3.112832
RB_p_mrmir000000700	mmu-miR-5626-3p:MIMAT0022382	6.78302	7.562860487	8.338079	7.19539
RB_p_mrmir0000001200	mmu-miR-5626-5p:MIMAT0022381	6.513532	4.281676822	6.679711	3.203153
RB_p_mrmir000000180	mmu-miR-5627-3p:MIMAT0022386	6.487675	4.439978467	7.538129	3.778625
RB_p_mrmir000000317	mmu-miR-5627-5p:MIMAT0022385	8.146738	4.628572449	6.666045	6.464986
RB_p_mrmir000000585	mmu-miR-568:MIMAT0004892	9.223575	4.451927096	8.731492	10.46807
RB_p_mrmir00000021	mmu-miR-5709-3p:MIMAT0027104	4.646477	6.472329831	8.155153	6.799852
RB_p_mrmir000000521	mmu-miR-5709-5p:MIMAT0022504	7.008568	6.646214104	1.975217	5.383147
RB_p_mrmir0000001828	mmu-miR-5710:MIMAT0022505	5.080087	6.295879722	6.35552	6.679316
RB_p_mrmir000000675	mmu-miR-574-3p:MIMAT0004894	11.17051	10.7506813	11.75214	12.78399
RB_p_mrmir0000001910	mmu-miR-574-5p:MIMAT0004893	11.44184	10.17293566	12.10314	12.49597
RB_p_mrmir0000001422	mmu-miR-582-3p:MIMAT0005292	3.957071	3.639642527	5.785674	1.09441
RB_p_mrmir000000518	mmu-miR-582-5p:MIMAT0005291	6.817871	4.970621605	6.484788	4.376357
RB_p_mrmir0000001452	mmu-miR-590-3p:MIMAT0004896	7.594586	6.426734268	2.925219	2.841778
RB_p_mrmir0000001148	mmu-miR-590-5p:MIMAT0004895	7.203238	5.252769302	1.727634	2.30095
RB_p_mrmir0000001680	mmu-miR-592-3p:MIMAT0017234	8.566134	9.545106397	7.762222	6.77035
RB_p_mrmir000000609	mmu-miR-592-5p:MIMAT0003730	7.685462	5.138039471	5.918352	4.57609
RB_p_mrmir0000001481	mmu-miR-598-3p:MIMAT0004942	6.612115	8.309007378	6.781583	5.501623
RB_p_mrmir0000001222	mmu-miR-598-5p:MIMAT0017283	11.48733	10.92651557	8.691157	8.72531
RB_p_mrmir0000002278	mmu-miR-599:MIMAT0012772	8.669998	2.421965312	2.697775	6.14899
RB_p_mrmir0000001713	mmu-miR-615-3p:MIMAT0003783	6.902553	3.897607389	6.832286	3.162725
RB_p_mrmir0000001306	mmu-miR-615-5p:MIMAT0004837	8.115358	5.993190376	7.740258	5.915304
RB_p_mrmir0000001208	mmu-miR-6236:MIMAT0024857	7.517104	8.049099169	7.14043	7.55877
RB_p_mrmir0000002166	mmu-miR-6237:MIMAT0024858	5.446333	5.712047847	5.858129	6.508242
RB_p_mrmir0000002184	mmu-miR-6238:MIMAT0024859	11.20476	12.70089902	12.06339	12.47122
RB_p_mrmir0000001524	mmu-miR-6239:MIMAT0024860	9.80013	11.79061742	9.242096	11.08812
RB_p_mrmir000000798	mmu-miR-6240:MIMAT0024861	11.07492	12.75174897	11.80229	13.0015
RB_p_mrmir000000676	mmu-miR-6241:MIMAT0024862	6.518961	7.720943298	2.405925	2.900261
RB_p_mrmir0000001303	mmu-miR-6244:MIMAT0024864	7.720629	6.375112294	7.197614	3.989438
RB_p_mrmir0000001031	mmu-miR-6335:MIMAT0025077	6.632808	5.129043684	6.108943	5.067645
RB_p_mrmir0000001776	mmu-miR-6336:MIMAT0025079	4.372743	5.348825747	4.721058	5.487257
RB_p_mrmir0000001890	mmu-miR-6337:MIMAT0025080	8.428513	4.618638369	7.85615	3.910493
RB_p_mrmir0000001069	mmu-miR-6338:MIMAT0025081	5.092208	6.296112126	4.67342	6.671982
RB_p_mrmir0000001169	mmu-miR-6339:MIMAT0025082	4.990135	2.440995482	6.145036	5.337674
RB_p_mrmir0000001349	mmu-miR-6340:MIMAT0025083	6.3979	4.546258464	4.809421	1.969589
RB_p_mrmir000000741	mmu-miR-6341:MIMAT0025084	4.617177	4.206545112	4.975271	3.738376
RB_p_mrmir000000697	mmu-miR-6342:MIMAT0025085	6.406211	7.192000285	6.407295	5.652778
RB_p_mrmir0000002083	mmu-miR-6343:MIMAT0025086	8.109626	4.815188975	7.664042	4.547101

RB_p_mrmir0000001409	mmu-miR-6344:MIMAT0025087	5.163807	6.299646033	5.549686	7.696169
RB_p_mrmir0000001511	mmu-miR-6345:MIMAT0025088	7.812439	7.427539578	7.218881	7.211766
RB_p_mrmir0000001521	mmu-miR-6346:MIMAT0025089	5.875171	2.09474366	7.06423	6.1741
RB_p_mrmir000000748	mmu-miR-6347:MIMAT0025090	9.440913	8.75214783	8.873566	6.546615
RB_p_mrmir0000001656	mmu-miR-6348:MIMAT0025091	9.630193	8.149761493	10.91002	10.56272
RB_p_mrmir0000001992	mmu-miR-6349:MIMAT0025092	11.81137	10.85932695	11.55555	11.23389
RB_p_mrmir0000001520	mmu-miR-6350:MIMAT0025093	3.668767	2.883728821	7.193534	5.894197
RB_p_mrmir0000001523	mmu-miR-6351:MIMAT0025094	7.128267	6.360463864	7.185837	5.782745
RB_p_mrmir0000002309	mmu-miR-6352:MIMAT0025095	7.894866	8.552192139	5.119936	6.362255
RB_p_mrmir0000001518	mmu-miR-6353:MIMAT0025096	3.592522	1.88179175	4.884925	6.973766
RB_p_mrmir0000001949	mmu-miR-6354:MIMAT0025097	7.440427	6.573303423	7.100468	4.582187
RB_p_mrmir000000654	mmu-miR-6355:MIMAT0025098	0.298055	4.499258772	4.63435	5.02894
RB_p_mrmir0000001699	mmu-miR-6356:MIMAT0025099	7.941249	8.733246714	5.924033	6.186173
RB_p_mrmir0000001510	mmu-miR-6357:MIMAT0025100	6.624069	6.261520843	6.891881	7.972115
RB_p_mrmir000000753	mmu-miR-6358:MIMAT0025101	7.432293	6.50671453	6.656799	3.929252
RB_p_mrmir000000910	mmu-miR-6359:MIMAT0025102	7.220188	6.029785361	7.445059	6.72609
RB_p_mrmir0000001556	mmu-miR-6360:MIMAT0025103	10.56788	9.087518266	10.24367	10.177
RB_p_mrmir0000002022	mmu-miR-6361:MIMAT0025104	6.502007	5.882359705	7.55188	2.824734
RB_p_mrmir0000001391	mmu-miR-6362:MIMAT0025106	6.461404	4.929137755	4.35503	3.780307
RB_p_mrmir0000002088	mmu-miR-6363:MIMAT0025107	7.848341	4.589058937	2.066859	6.159689
RB_p_mrmir0000001740	mmu-miR-6364:MIMAT0025108	8.089321	7.660163601	4.665233	7.082038
RB_p_mrmir0000002177	mmu-miR-6365:MIMAT0025109	4.563582	2.030884895	6.576524	1.496133
RB_p_mrmir000000354	mmu-miR-6366:MIMAT0025110	10.80839	8.807628809	10.29375	8.236581
RB_p_mrmir0000001704	mmu-miR-6367:MIMAT0025111	7.216512	3.230380759	7.031226	3.461147
RB_p_mrmir0000001055	mmu-miR-6368:MIMAT0025112	13.23034	13.31528005	14.50887	11.75171
RB_p_mrmir0000002026	mmu-miR-6369:MIMAT0025113	7.676664	8.117480091	7.804468	4.161605
RB_p_mrmir0000001190	mmu-miR-6370:MIMAT0025114	12.58051	10.47287127	12.73147	12.80381
RB_p_mrmir0000002188	mmu-miR-6371:MIMAT0025115	3.246733	7.887389257	4.227293	7.642957
RB_p_mrmir000000293	mmu-miR-6372:MIMAT0025116	7.028486	4.79000079	7.566613	5.900066
RB_p_mrmir0000001590	mmu-miR-6373:MIMAT0025117	7.815316	7.868960999	7.544946	8.2227
RB_p_mrmir000000119	mmu-miR-6374:MIMAT0025118	6.61491	5.342523062	5.931961	1.745892
RB_p_mrmir0000001131	mmu-miR-6375:MIMAT0025119	6.180707	8.073910907	7.465559	4.692697
RB_p_mrmir000000754	mmu-miR-6376:MIMAT0025120	4.591124	6.77273432	5.450582	2.538136
RB_p_mrmir0000001688	mmu-miR-6377:MIMAT0025122	8.006058	7.675543982	7.366402	6.706102
RB_p_mrmir0000002072	mmu-miR-6378:MIMAT0025124	11.11598	10.23149141	10.01614	11.83912
RB_p_mrmir0000001709	mmu-miR-6379:MIMAT0025125	6.270891	7.126599847	8.011474	3.825668
RB_p_mrmir0000002086	mmu-miR-6380:MIMAT0025126	5.585976	4.372401374	6.36067	3.548776
RB_p_mrmir000000716	mmu-miR-6381:MIMAT0025127	7.710038	3.948034779	7.546115	8.405037
RB_p_mrmir0000001989	mmu-miR-6382:MIMAT0025128	11.08691	9.293386264	9.158623	9.946583
RB_p_mrmir0000001412	mmu-miR-6383:MIMAT0025129	3.231177	7.787027624	6.11481	4.659485
RB_p_mrmir0000001254	mmu-miR-6384:MIMAT0025130	6.755172	6.842889385	6.45347	6.627146

RB_p_mrmir0000001176	mmu-miR-6385:MIMAT0025131	13.88021	12.90294071	14.18863	14.22219
RB_p_mrmir000000783	mmu-miR-6386:MIMAT0025133	9.360593	8.234544321	8.536514	8.771273
RB_p_mrmir0000001986	mmu-miR-6387:MIMAT0025134	5.503459	1.706866127	5.32496	3.23533
RB_p_mrmir0000001931	mmu-miR-6388:MIMAT0025135	9.696421	9.233185804	10.49991	9.881647
RB_p_mrmir000000745	mmu-miR-6389:MIMAT0025137	6.631462	5.530149965	5.952425	-0.06931
RB_p_mrmir0000001130	mmu-miR-6390:MIMAT0025139	9.015947	9.232789477	1.814433	4.428558
RB_p_mrmir0000001888	mmu-miR-6391:MIMAT0025140	10.57743	11.08894354	9.988382	9.867833
RB_p_mrmir0000001136	mmu-miR-6392-3p:MIMAT0025142	6.427306	3.543990672	6.690681	6.773516
RB_p_mrmir0000001806	mmu-miR-6392-5p:MIMAT0025141	7.464562	6.843323725	7.568589	5.429967
RB_p_mrmir0000001035	mmu-miR-6393:MIMAT0025143	8.772308	6.800394834	7.157956	6.156204
RB_p_mrmir0000001708	mmu-miR-6394:MIMAT0025144	9.424267	4.964118778	5.877301	5.678451
RB_p_mrmir0000001048	mmu-miR-6395:MIMAT0025146	7.506989	2.338459939	7.291788	2.934838
RB_p_mrmir0000001297	mmu-miR-6396:MIMAT0025147	7.373458	7.767321898	6.850556	5.37791
RB_p_mrmir0000001998	mmu-miR-6397:MIMAT0025149	6.807077	4.02753021	7.189802	4.378377
RB_p_mrmir0000001347	mmu-miR-6398:MIMAT0025150	7.778099	9.010002719	6.11386	5.899785
RB_p_mrmir0000002237	mmu-miR-6399:MIMAT0025151	5.679742	7.873519487	6.331935	5.403348
RB_p_mrmir0000002225	mmu-miR-6400:MIMAT0025152	7.66647	7.046640129	5.662263	3.043559
RB_p_mrmir0000002170	mmu-miR-6401:MIMAT0025153	8.231217	6.146959126	7.039228	6.410458
RB_p_mrmir0000001143	mmu-miR-6402:MIMAT0025154	6.536133	8.653654715	6.074688	6.298371
RB_p_mrmir0000001174	mmu-miR-6403:MIMAT0025155	4.727162	6.726740659	4.659629	4.159015
RB_p_mrmir0000001389	mmu-miR-6404:MIMAT0025156	10.59621	9.22952796	11.52494	10.51305
RB_p_mrmir0000001388	mmu-miR-6405:MIMAT0025157	10.5731	10.99648846	11.35611	10.65187
RB_p_mrmir000000918	mmu-miR-6406:MIMAT0025159	8.084694	5.158064601	6.52374	3.799238
RB_p_mrmir0000002030	mmu-miR-6407:MIMAT0025160	3.398602	5.608737689	7.237858	4.943772
RB_p_mrmir000000666	mmu-miR-6408:MIMAT0025161	9.420858	7.797411596	8.755744	7.716346
RB_p_mrmir0000001961	mmu-miR-6409:MIMAT0025162	8.559735	4.951391319	8.690999	6.270877
RB_p_mrmir0000002023	mmu-miR-6410:MIMAT0025163	4.905833	7.449564704	8.135001	5.262434
RB_p_mrmir0000001522	mmu-miR-6411:MIMAT0025164	3.392579	3.355052002	8.423596	-0.52096
RB_p_mrmir0000001735	mmu-miR-6412:MIMAT0025165	7.073909	7.106836904	8.396588	8.023722
RB_p_mrmir0000002021	mmu-miR-6413:MIMAT0025166	9.922754	8.811698634	10.06717	8.455896
RB_p_mrmir000000968	mmu-miR-6414:MIMAT0025168	5.097718	3.557585508	6.955334	2.160207
RB_p_mrmir0000001149	mmu-miR-6415:MIMAT0025169	3.917182	5.660063173	6.176294	4.976522
RB_p_mrmir0000001172	mmu-miR-6416-3p:MIMAT0025171	11.88862	11.21990162	12.23399	11.80522
RB_p_mrmir0000001006	mmu-miR-6416-5p:MIMAT0025170	8.04102	6.711145027	5.577317	1.539445
RB_p_mrmir0000001505	mmu-miR-6417:MIMAT0025172	7.652683	7.325397834	5.837123	7.472732
RB_p_mrmir000000253	mmu-miR-6418-3p:MIMAT0025174	7.832003	6.607595406	6.059145	4.82575
RB_p_mrmir0000001663	mmu-miR-6418-5p:MIMAT0025173	12.22423	12.70585181	13.43778	12.47776
RB_p_mrmir000000696	mmu-miR-6419:MIMAT0025175	6.178731	8.077431481	6.472062	6.426426
RB_p_mrmir000000232	mmu-miR-6420:MIMAT0025176	5.580198	6.022402834	2.948237	5.473826
RB_p_mrmir000000683	mmu-miR-6481:MIMAT0027339	3.621916	1.60406037	4.436641	4.955158
RB_p_mrmir0000001685	mmu-miR-6516-3p:MIMAT0027344	4.662939	4.958808053	6.846091	2.908457

RB_p_mrmir0000002303	mmu-miR-6516-5p:MIMAT0027343	7.755471	8.335180545	8.872421	6.196204
RB_p_mrmir000000143	mmu-miR-652-3p:MIMAT0003711	6.803873	4.524701829	7.776133	0.272788
RB_p_mrmir000000632	mmu-miR-652-5p:MIMAT0017260	9.546554	8.038300491	8.309516	7.084993
RB_p_mrmir0000002196	mmu-miR-653-3p:MIMAT0017284	5.504025	6.277451737	4.840857	4.094535
RB_p_mrmir0000001400	mmu-miR-653-5p:MIMAT0004943	8.9296	8.039324351	5.462655	4.866477
RB_p_mrmir0000002182	mmu-miR-6537-3p:MIMAT0025582	7.607313	7.430411737	5.924497	8.187745
RB_p_mrmir0000001377	mmu-miR-6537-5p:MIMAT0025581	5.078708	6.834792025	5.875033	7.300309
RB_p_mrmir000000919	mmu-miR-6538:MIMAT0025583	11.65317	12.00336756	12.34288	12.14722
RB_p_mrmir0000001180	mmu-miR-6539:MIMAT0025584	6.03633	6.847719765	7.898747	8.694712
RB_p_mrmir0000001787	mmu-miR-6540-3p:MIMAT0025586	7.042493	9.034396968	7.857666	5.893045
RB_p_mrmir000000967	mmu-miR-6540-5p:MIMAT0025585	7.658815	6.274680337	8.792976	7.555151
RB_p_mrmir000000661	mmu-miR-6541:MIMAT0025588	5.546969	5.893450824	4.892814	4.953279
RB_p_mrmir0000001596	mmu-miR-654-3p:MIMAT0004898	6.354581	7.159097934	6.601145	5.611988
RB_p_mrmir0000002060	mmu-miR-654-5p:MIMAT0004897	3.767503	7.943305339	5.697628	7.55173
RB_p_mrmir0000001146	mmu-miR-6546-3p:MIMAT0029793	7.617833	7.599379633	8.39921	7.922387
RB_p_mrmir000000401	mmu-miR-6546-5p:MIMAT0029792	5.149159	5.507396091	5.889467	3.656788
RB_p_mrmir0000001611	mmu-miR-664-3p:MIMAT0012774	6.772817	3.865183106	6.679011	3.705822
RB_p_mrmir0000001053	mmu-miR-664-5p:MIMAT0017353	9.01248	6.08121165	7.516587	8.587573
RB_p_mrmir000000197	mmu-miR-665-3p:MIMAT0003733	8.700696	4.090424669	7.629902	6.879051
RB_p_mrmir000000433	mmu-miR-665-5p:MIMAT0017238	8.215232	7.481546596	8.312087	5.857046
RB_p_mrmir0000001280	mmu-miR-666-3p:MIMAT0004823	4.632533	7.642987896	6.261555	4.432553
RB_p_mrmir000000350	mmu-miR-666-5p:MIMAT0003737	8.716846	9.249169613	8.614643	8.957444
RB_p_mrmir0000001854	mmu-miR-667-3p:MIMAT0003734	7.765455	7.967170968	8.368138	9.134393
RB_p_mrmir000000948	mmu-miR-667-5p:MIMAT0017239	9.198602	8.80615149	10.30324	8.334127
RB_p_mrmir0000002096	mmu-miR-668-3p:MIMAT0003732	7.873875	6.676797516	7.446463	5.239292
RB_p_mrmir0000001336	mmu-miR-668-5p:MIMAT0017237	8.381839	6.500017404	7.726225	4.968146
RB_p_mrmir000000186	mmu-miR-669a-3-3p:MIMAT0017251	8.446582	7.748645576	9.513515	10.60985
RB_p_mrmir000000185	mmu-miR-669a-3p:MIMAT0017243;mmu-miR-669o-3p:MIMAT0017347	7.749762	8.382984544	10.92739	11.91156
RB_p_mrmir000000498	mmu-miR-669a-5p:MIMAT0003477;mmu-miR-669p-5p:MIMAT0014889	7.355682	6.92997987	8.251237	8.9265
RB_p_mrmir000000765	mmu-miR-669b-3p:MIMAT0017250	9.199856	7.934423838	8.425259	10.76295
RB_p_mrmir000000504	mmu-miR-669b-5p:MIMAT0003476	7.642279	7.139324588	8.813038	8.783549
RB_p_mrmir0000001453	mmu-miR-669c-3p:MIMAT0017253	9.535295	9.304553448	9.337156	11.67532
RB_p_mrmir000000525	mmu-miR-669c-5p:MIMAT0003479	10.42987	9.682460607	11.94155	11.25439
RB_p_mrmir000000533	mmu-miR-669d-2-3p:MIMAT0014884	7.862536	5.99682824	6.591946	9.554044
RB_p_mrmir0000001566	mmu-miR-669d-3p:MIMAT0017324	9.498774	7.024936375	7.863073	10.18731
RB_p_mrmir000000288	mmu-miR-669d-5p:MIMAT0005833	8.722005	7.946980954	8.630867	9.322897
RB_p_mrmir0000001848	mmu-miR-669e-3p:MIMAT0017330	9.789852	8.810966269	11.04836	11.48059
RB_p_mrmir0000002115	mmu-miR-669e-5p:MIMAT0005853	5.015534	7.253755907	8.559368	8.259582
RB_p_mrmir000000766	mmu-miR-669f-3p:MIMAT0005839	10.10707	9.641238843	12.03409	11.94318
RB_p_mrmir000000497	mmu-miR-669f-5p:MIMAT0017327	6.632534	7.382952622	11.06408	10.7297

RB_p_mrmir0000001941	mmu-miR-669g:MIMAT0005832	3.53877	3.027577947	4.798926	4.447059
RB_p_mrmir0000001583	mmu-miR-669h-3p:MIMAT0005842	8.807145	7.753442308	9.976789	11.39879
RB_p_mrmir000000567	mmu-miR-669h-5p:MIMAT0005841	6.725912	7.58675623	8.11641	6.65948
RB_p_mrmir0000001939	mmu-miR-669i:MIMAT0005840	8.774158	8.032693522	6.918496	5.415566
RB_p_mrmir0000001940	mmu-miR-669j:MIMAT0005838	7.207825	7.193806149	7.083524	5.104005
RB_p_mrmir0000001584	mmu-miR-669k-3p:MIMAT0005831	5.283988	4.491375507	5.684182	5.24419
RB_p_mrmir0000002124	mmu-miR-669k-5p:MIMAT0017323	10.02968	10.20871081	10.27353	7.722525
RB_p_mrmir000000534	mmu-miR-669l-3p:MIMAT0017345	7.814892	7.014395202	7.058371	9.490917
RB_p_mrmir000000496	mmu-miR-669l-5p:MIMAT0009418	8.249835	6.823781343	9.64432	9.823354
RB_p_mrmir000000535	mmu-miR-669m-3p:MIMAT0009419	7.128671	3.719628743	6.592301	9.331299
RB_p_mrmir0000002144	mmu-miR-669m-5p:MIMAT0017346;mmu-miR-466m-5p:MIMAT0014882	9.154533	8.30452146	10.44775	10.8006
RB_p_mrmir000000611	mmu-miR-669n:MIMAT0009427	10.35162	9.099126323	11.87023	11.51671
RB_p_mrmir0000001557	mmu-miR-669o-5p:MIMAT0009421	7.347954	6.223298525	7.668906	7.65498
RB_p_mrmir000000759	mmu-miR-669p-3p:MIMAT0014890	10.07002	9.480717479	10.9199	11.56182
RB_p_mrmir0000002288	mmu-miR-670-3p:MIMAT0017242	4.965124	1.351148509	5.776665	4.193964
RB_p_mrmir000000548	mmu-miR-670-5p:MIMAT0003736	8.70206	4.980916184	9.300147	8.929786
RB_p_mrmir0000001719	mmu-miR-671-3p:MIMAT0004821	4.948616	5.409618794	4.224334	4.587047
RB_p_mrmir000000797	mmu-miR-6715-3p:MIMAT0029877	5.981127	3.736322287	5.951361	5.39579
RB_p_mrmir000000178	mmu-miR-6715-5p:MIMAT0029876	4.613403	7.256703863	7.715878	7.269681
RB_p_mrmir000000373	mmu-miR-671-5p:MIMAT0003731	12.49244	12.38383629	12.96663	12.12714
RB_p_mrmir000000164	mmu-miR-672-3p:MIMAT0017241	6.980825	7.581319614	6.983209	6.859878
RB_p_mrmir0000001907	mmu-miR-672-5p:MIMAT0003735	10.30341	7.862885032	11.02948	10.667
RB_p_mrmir0000001717	mmu-miR-673-3p:MIMAT0004824	8.113652	6.428038058	6.5259	4.977168
RB_p_mrmir000000993	mmu-miR-673-5p:MIMAT0003739	5.931546	1.898358792	5.753442	4.223856
RB_p_mrmir000000660	mmu-miR-674-3p:MIMAT0003741	6.750551	5.862893247	5.466923	7.436828
RB_p_mrmir0000001183	mmu-miR-674-5p:MIMAT0003740	9.105687	6.702987829	9.262087	7.981461
RB_p_mrmir0000001074	mmu-miR-675-3p:MIMAT0003726	6.964748	5.26175011	6.03602	3.880534
RB_p_mrmir0000002066	mmu-miR-675-5p:MIMAT0003725	7.939506	6.361980487	7.040468	6.661936
RB_p_mrmir000000867	mmu-miR-676-3p:MIMAT0003782	7.317664	7.505749043	6.856868	1.530266
RB_p_mrmir000000247	mmu-miR-676-5p:MIMAT0003781	5.372477	3.967808412	6.633396	4.15389
RB_p_mrmir000000782	mmu-miR-6769b-3p:MIMAT0028041	6.890886	7.687739331	8.237064	5.193437
RB_p_mrmir000000890	mmu-miR-6769b-5p:MIMAT0028040	13.50755	14.04921268	13.50996	13.90887
RB_p_mrmir0000001120	mmu-miR-677-3p:MIMAT0017246	11.45028	12.35597892	11.38483	10.51436
RB_p_mrmir0000002199	mmu-miR-677-5p:MIMAT0003451	4.84829	4.495582596	7.24391	1.764509
RB_p_mrmir0000001356	mmu-miR-678:MIMAT0003452	12.68328	11.22267205	11.97496	11.72098
RB_p_mrmir000000333	mmu-miR-679-3p:MIMAT0017248	6.221337	8.221029516	9.11314	3.814683
RB_p_mrmir0000001257	mmu-miR-679-5p:MIMAT0003455	6.461098	2.759208192	6.370954	4.804952
RB_p_mrmir0000001292	mmu-miR-680:MIMAT0003457	9.263594	7.419297973	8.644258	8.228725
RB_p_mrmir000000703	mmu-miR-681:MIMAT0003458	6.553161	6.693221075	7.751051	4.290936
RB_p_mrmir0000001030	mmu-miR-682:MIMAT0003459	7.376704	3.434666109	5.970039	5.151879
RB_p_mrmir000000887	mmu-miR-683:MIMAT0003461	8.226391	8.495887418	5.793964	4.53323

RB_p_mrmir000000499	mmu-miR-684:MIMAT0003462	6.404528	4.090227469	7.103153	3.678664
RB_p_mrmir000000601	mmu-miR-686:MIMAT0003464	9.555156	7.65050508	7.580303	7.679304
RB_p_mrmir000000987	mmu-miR-687:MIMAT0003466	4.724573	2.100129744	5.740128	5.751141
RB_p_mrmir0000001741	mmu-miR-688:MIMAT0003467	7.301914	7.56184465	6.135029	6.560804
RB_p_mrmir0000002293	mmu-miR-6896-3p:MIMAT0027693	6.172803	3.093140884	6.901421	4.512764
RB_p_mrmir0000002314	mmu-miR-6896-5p:MIMAT0027692	8.514357	8.994055508	7.779925	8.382454
RB_p_mrmir000000945	mmu-miR-6897-3p:MIMAT0027695	7.192593	7.258556626	7.832705	8.392586
RB_p_mrmir0000002052	mmu-miR-6897-5p:MIMAT0027694	6.466782	5.629408041	5.853595	4.179926
RB_p_mrmir000000112	mmu-miR-6898-3p:MIMAT0027697	0.719177	4.170555089	4.366348	2.046355
RB_p_mrmir0000002085	mmu-miR-6898-5p:MIMAT0027696	15.07459	14.63914822	14.66919	14.82794
RB_p_mrmir0000002275	mmu-miR-6899-3p:MIMAT0027699	8.108603	2.554772242	6.294558	5.006142
RB_p_mrmir000000335	mmu-miR-6899-5p:MIMAT0027698	10.03353	10.8950325	10.17963	10.36931
RB_p_mrmir00000019	mmu-miR-690:MIMAT0003469	9.685806	10.93290032	10.66034	11.78897
RB_p_mrmir0000002065	mmu-miR-6900-3p:MIMAT0027701	6.647257	4.673220914	5.628588	4.928657
RB_p_mrmir0000001943	mmu-miR-6900-5p:MIMAT0027700	8.563995	9.309888216	8.751679	8.894435
RB_p_mrmir0000001135	mmu-miR-6901-3p:MIMAT0027703	7.872488	7.435369076	4.513713	3.515513
RB_p_mrmir0000001936	mmu-miR-6901-5p:MIMAT0027702	9.909431	8.606558857	10.25936	9.396953
RB_p_mrmir000000828	mmu-miR-6902-3p:MIMAT0027705	4.742984	3.724028169	7.948365	7.480387
RB_p_mrmir0000002212	mmu-miR-6902-5p:MIMAT0027704	5.618995	5.750788971	7.306628	2.580239
RB_p_mrmir0000002146	mmu-miR-6903-3p:MIMAT0027707	4.78872	4.068832564	8.789662	8.112912
RB_p_mrmir0000002256	mmu-miR-6903-5p:MIMAT0027706	3.626026	8.276143911	5.384367	2.924453
RB_p_mrmir0000001796	mmu-miR-6904-3p:MIMAT0027709	8.723069	4.963058152	5.684991	4.941264
RB_p_mrmir0000001730	mmu-miR-6904-5p:MIMAT0027708	6.985546	4.192328153	7.360692	8.060387
RB_p_mrmir00000034	mmu-miR-6905-3p:MIMAT0027711	4.947731	9.299578208	4.563573	3.843649
RB_p_mrmir000000276	mmu-miR-6905-5p:MIMAT0027710	8.353052	7.701761679	7.220104	7.008804
RB_p_mrmir0000001129	mmu-miR-6906-3p:MIMAT0027713	3.869156	5.283350191	7.595973	5.696748
RB_p_mrmir000000573	mmu-miR-6906-5p:MIMAT0027712	7.805435	7.369311827	9.557691	6.757091
RB_p_mrmir00000061	mmu-miR-6907-3p:MIMAT0027715	6.898114	5.088539275	7.676084	7.082753
RB_p_mrmir0000002224	mmu-miR-6907-5p:MIMAT0027714	11.6481	10.15832364	10.98419	10.78347
RB_p_mrmir000000170	mmu-miR-6908-3p:MIMAT0027717	6.249697	5.637618187	5.202808	5.506064
RB_p_mrmir0000002241	mmu-miR-6908-5p:MIMAT0027716	9.159569	8.715342186	9.085435	8.408425
RB_p_mrmir0000001956	mmu-miR-6909-3p:MIMAT0027719	7.503636	6.507715255	7.0658	6.500716
RB_p_mrmir000000730	mmu-miR-6909-5p:MIMAT0027718	12.17696	10.52262083	10.38186	10.1064
RB_p_mrmir000000597	mmu-miR-691:MIMAT0003470	12.05197	11.32344027	11.95289	11.94331
RB_p_mrmir0000001684	mmu-miR-6910-3p:MIMAT0027721	5.830212	5.805746351	6.878028	5.368246
RB_p_mrmir0000002053	mmu-miR-6910-5p:MIMAT0027720	10.65816	8.075813897	9.241364	8.857636
RB_p_mrmir0000001628	mmu-miR-6911-3p:MIMAT0027723	5.884311	6.927315466	7.37328	5.683646
RB_p_mrmir0000002044	mmu-miR-6911-5p:MIMAT0027722	9.739162	8.267190857	8.514553	5.54089
RB_p_mrmir000000970	mmu-miR-6912-3p:MIMAT0027725	5.783786	7.766753798	4.763372	2.824071
RB_p_mrmir0000001457	mmu-miR-6912-5p:MIMAT0027724	10.41055	10.31045983	10.63309	8.591556
RB_p_mrmir0000001777	mmu-miR-6913-3p:MIMAT0027727	7.219738	3.729989831	2.401795	5.33085

RB_p_mrmir0000002038	mmu-miR-6913-5p:MIMAT0027726	7.142485	6.982218817	4.984933	6.01756
RB_p_mrmir0000001819	mmu-miR-6914-3p:MIMAT0027729	3.69255	6.445247842	6.60468	4.784364
RB_p_mrmir0000001729	mmu-miR-6914-5p:MIMAT0027728	9.565888	6.559388963	8.692269	8.474195
RB_p_mrmir000000638	mmu-miR-6915-3p:MIMAT0027731	6.165749	3.721687016	8.066767	6.890296
RB_p_mrmir000000440	mmu-miR-6915-5p:MIMAT0027730	9.069847	8.217549105	9.82906	9.715406
RB_p_mrmir0000001099	mmu-miR-6916-3p:MIMAT0027733	7.218067	7.686922852	3.424227	4.326319
RB_p_mrmir000000710	mmu-miR-6916-5p:MIMAT0027732	9.617146	6.987899314	9.587189	7.913567
RB_p_mrmir0000001348	mmu-miR-6917-3p:MIMAT0027735	8.043307	8.179551986	4.920206	7.528165
RB_p_mrmir0000002128	mmu-miR-6917-5p:MIMAT0027734	10.02903	8.702909158	8.994025	7.171123
RB_p_mrmir0000001882	mmu-miR-6918-3p:MIMAT0027737	7.008405	6.298674897	4.830638	8.322748
RB_p_mrmir0000001973	mmu-miR-6918-5p:MIMAT0027736	7.376056	6.052613097	7.85578	5.174894
RB_p_mrmir0000001228	mmu-miR-6919-3p:MIMAT0027739	7.367536	2.704528358	5.75483	4.800528
RB_p_mrmir0000001537	mmu-miR-6919-5p:MIMAT0027738	8.190065	9.588168741	6.603435	7.218956
RB_p_mrmir000000557	mmu-miR-692:MIMAT0003471	4.648627	4.09454013	6.480244	5.952825
RB_p_mrmir000000684	mmu-miR-6920-3p:MIMAT0027741	7.356708	5.680831394	8.03071	8.238248
RB_p_mrmir000000163	mmu-miR-6920-5p:MIMAT0027740	6.257089	4.743085035	7.108107	6.730625
RB_p_mrmir0000001862	mmu-miR-6921-3p:MIMAT0027743	6.438985	5.800908583	7.280551	6.719133
RB_p_mrmir0000001894	mmu-miR-6921-5p:MIMAT0027742	12.25563	12.16011709	13.06284	12.8651
RB_p_mrmir0000002154	mmu-miR-6922-3p:MIMAT0027745	6.535426	2.879872314	7.571766	5.18323
RB_p_mrmir0000002135	mmu-miR-6922-5p:MIMAT0027744	12.56769	10.89221986	13.06859	12.49257
RB_p_mrmir000000169	mmu-miR-6923-3p:MIMAT0027747	8.220122	3.816394447	6.964306	5.809092
RB_p_mrmir0000001371	mmu-miR-6923-5p:MIMAT0027746	12.81313	11.73067495	12.24038	12.7313
RB_p_mrmir0000001732	mmu-miR-6924-3p:MIMAT0027749	5.919605	3.014323125	5.124207	5.796032
RB_p_mrmir000000310	mmu-miR-6924-5p:MIMAT0027748	10.43065	7.863026478	8.574707	8.486457
RB_p_mrmir0000001701	mmu-miR-6925-3p:MIMAT0027751	3.486997	2.525597481	7.730985	6.569411
RB_p_mrmir0000001885	mmu-miR-6925-5p:MIMAT0027750	10.37387	6.864460147	10.34979	8.955719
RB_p_mrmir0000001633	mmu-miR-6926-3p:MIMAT0027753	5.633737	4.352579083	7.313808	5.086447
RB_p_mrmir0000001675	mmu-miR-6926-5p:MIMAT0027752	11.59086	9.42053292	11.61716	11.31512
RB_p_mrmir000000884	mmu-miR-6927-3p:MIMAT0027755	4.625584	3.793329396	6.799142	0.911315
RB_p_mrmir0000001373	mmu-miR-6927-5p:MIMAT0027754	7.754075	6.693634581	6.822812	5.90058
RB_p_mrmir00000087	mmu-miR-6928-3p:MIMAT0027757	7.031893	5.289862621	8.264931	4.876269
RB_p_mrmir000000432	mmu-miR-6928-5p:MIMAT0027756	5.167425	5.133646632	7.605472	4.458761
RB_p_mrmir000000735	mmu-miR-6929-3p:MIMAT0027759	7.006744	5.100192789	5.561042	6.347984
RB_p_mrmir000000419	mmu-miR-6929-5p:MIMAT0027758	8.085037	3.645359283	7.622356	8.399491
RB_p_mrmir0000001050	mmu-miR-6930-3p:MIMAT0027761	5.635537	6.055986504	5.146859	4.594619
RB_p_mrmir000000104	mmu-miR-6930-5p:MIMAT0027760	7.538344	9.405372986	7.320336	4.942606
RB_p_mrmir000000829	mmu-miR-6931-3p:MIMAT0027763	6.656157	4.878441359	6.289144	3.080132
RB_p_mrmir0000002056	mmu-miR-6931-5p:MIMAT0027762	14.66648	14.2622032	14.43842	13.55002
RB_p_mrmir0000001823	mmu-miR-6932-3p:MIMAT0027765	4.257459	4.107931833	5.719926	2.038669
RB_p_mrmir0000002273	mmu-miR-6932-5p:MIMAT0027764	9.115601	7.654525452	6.288401	4.444251
RB_p_mrmir000000450	mmu-miR-6933-3p:MIMAT0027767	7.353577	6.64986322	7.104973	8.62298

RB_p_mrmir0000002020	mmu-miR-6933-5p:MIMAT0027766	6.645685	6.28171834	7.96102	0.917132
RB_p_mrmir0000001189	mmu-miR-693-3p:MIMAT0004189	7.56382	6.651435481	7.299802	3.575517
RB_p_mrmir000000209	mmu-miR-6934-3p:MIMAT0027769	8.128332	8.317194838	7.77601	3.977266
RB_p_mrmir0000002268	mmu-miR-6934-5p:MIMAT0027768	14.70845	13.91185369	14.29476	14.17874
RB_p_mrmir0000002156	mmu-miR-6935-3p:MIMAT0027771	7.437745	9.412754969	6.47359	0.318877
RB_p_mrmir0000002134	mmu-miR-6935-5p:MIMAT0027770	7.255375	6.147503835	7.929988	6.95101
RB_p_mrmir000000701	mmu-miR-693-5p:MIMAT0003472	7.632166	7.355231983	6.278476	6.445535
RB_p_mrmir000000929	mmu-miR-6936-3p:MIMAT0027773	7.614969	6.027372989	7.863522	4.143342
RB_p_mrmir0000001365	mmu-miR-6936-5p:MIMAT0027772	5.990301	4.476869893	7.028004	-0.33103
RB_p_mrmir0000001630	mmu-miR-6937-3p:MIMAT0027775	7.394487	6.173833737	6.956165	6.693306
RB_p_mrmir0000001528	mmu-miR-6937-5p:MIMAT0027774	12.16627	12.38907776	11.95693	12.43142
RB_p_mrmir0000001682	mmu-miR-6938-3p:MIMAT0027777	6.754838	7.690718968	5.102283	5.868353
RB_p_mrmir0000002249	mmu-miR-6938-5p:MIMAT0027776	10.18695	8.423272184	10.97072	10.57525
RB_p_mrmir000000875	mmu-miR-6939-3p:MIMAT0027779	6.745695	5.375755299	5.990223	1.525642
RB_p_mrmir000000717	mmu-miR-6939-5p:MIMAT0027778	10.61051	8.933819335	10.88268	10.24415
RB_p_mrmir0000001020	mmu-miR-694:MIMAT0003474	4.66321	6.700021151	6.373115	4.74966
RB_p_mrmir0000002172	mmu-miR-6940-3p:MIMAT0027781	5.530545	5.929716832	2.565607	3.90131
RB_p_mrmir000000425	mmu-miR-6940-5p:MIMAT0027780	7.601873	9.286405215	6.79203	5.584439
RB_p_mrmir000000966	mmu-miR-6941-3p:MIMAT0027783	3.910009	7.466106785	8.462202	1.949701
RB_p_mrmir0000001061	mmu-miR-6941-5p:MIMAT0027782	9.30586	8.216869913	8.120702	8.699003
RB_p_mrmir0000002183	mmu-miR-6942-3p:MIMAT0027785	6.219507	5.382418581	6.942581	1.944477
RB_p_mrmir0000001536	mmu-miR-6942-5p:MIMAT0027784	9.629814	8.318261244	8.801964	8.720019
RB_p_mrmir0000001043	mmu-miR-6943-3p:MIMAT0027787	6.329537	7.607711183	6.380237	6.642232
RB_p_mrmir0000002055	mmu-miR-6943-5p:MIMAT0027786	8.950231	5.811246752	7.955306	4.98138
RB_p_mrmir0000001425	mmu-miR-6944-3p:MIMAT0027789	5.142306	5.372249536	8.048682	3.850925
RB_p_mrmir0000001363	mmu-miR-6944-5p:MIMAT0027788	14.36989	13.87628004	14.07818	13.92691
RB_p_mrmir0000001791	mmu-miR-6945-3p:MIMAT0027791	5.309425	5.800619444	6.595146	5.03986
RB_p_mrmir0000001728	mmu-miR-6945-5p:MIMAT0027790	7.616088	7.111572398	6.541774	5.951291
RB_p_mrmir0000002296	mmu-miR-6946-3p:MIMAT0027793	5.896675	3.025703514	6.723922	1.644107
RB_p_mrmir0000004	mmu-miR-6946-5p:MIMAT0027792	10.38393	9.1525649	11.80473	10.54636
RB_p_mrmir0000001188	mmu-miR-6947-3p:MIMAT0027795	8.062143	8.000757058	5.40016	1.320021
RB_p_mrmir000000771	mmu-miR-6947-5p:MIMAT0027794	10.04444	7.354376968	8.638328	9.204766
RB_p_mrmir0000002104	mmu-miR-6948-3p:MIMAT0027797	3.785469	6.583365903	3.410208	5.131919
RB_p_mrmir000000493	mmu-miR-6948-5p:MIMAT0027796	7.825184	6.596287543	6.088042	6.94368
RB_p_mrmir0000002203	mmu-miR-6949-3p:MIMAT0027799	5.779273	4.21854202	5.208834	3.013399
RB_p_mrmir00000031	mmu-miR-6949-5p:MIMAT0027798	9.380526	8.960692769	9.658916	8.763766
RB_p_mrmir000000332	mmu-miR-695:MIMAT0003481	5.350644	5.691667545	6.951765	7.59589
RB_p_mrmir0000001019	mmu-miR-6950-3p:MIMAT0027801	7.234916	7.468938057	4.006652	8.943636
RB_p_mrmir0000001726	mmu-miR-6950-5p:MIMAT0027800	10.62496	8.77447205	10.51457	9.114615
RB_p_mrmir0000001108	mmu-miR-6951-3p:MIMAT0027803	4.785432	2.657553731	6.569445	3.143771
RB_p_mrmir0000002274	mmu-miR-6951-5p:MIMAT0027802	5.853136	-0.28692731	5.313612	7.246408

RB_p_mrmir0000001781	mmu-miR-6952-3p:MIMAT0027805	6.354548	7.204069705	6.74264	7.352067
RB_p_mrmir000000581	mmu-miR-6952-5p:MIMAT0027804	5.716643	8.852023629	9.411804	8.545366
RB_p_mrmir0000001023	mmu-miR-6953-3p:MIMAT0027807	7.878986	7.086026917	8.327817	5.290968
RB_p_mrmir000000103	mmu-miR-6953-5p:MIMAT0027806	11.33652	8.99503215	10.19583	10.46562
RB_p_mrmir0000001933	mmu-miR-6954-3p:MIMAT0027809	7.117265	7.801328912	6.657266	7.444381
RB_p_mrmir0000002049	mmu-miR-6954-5p:MIMAT0027808	10.89857	10.16789919	10.72687	10.98733
RB_p_mrmir000000168	mmu-miR-6955-3p:MIMAT0027811	7.752687	3.959269068	7.825831	8.542164
RB_p_mrmir000000422	mmu-miR-6955-5p:MIMAT0027810	9.90025	7.630917984	8.458434	9.806854
RB_p_mrmir0000001858	mmu-miR-6956-3p:MIMAT0027813	2.711528	7.179868403	7.824381	2.501857
RB_p_mrmir0000001909	mmu-miR-6956-5p:MIMAT0027812	10.29088	8.176400358	8.62152	8.803561
RB_p_mrmir0000002008	mmu-miR-6957-3p:MIMAT0027815	5.556647	5.774038735	7.326054	7.735019
RB_p_mrmir000000688	mmu-miR-6957-5p:MIMAT0027814	9.242674	8.219664142	9.446294	9.573692
RB_p_mrmir0000001659	mmu-miR-6958-3p:MIMAT0027817	5.400518	6.580333404	8.032925	7.920337
RB_p_mrmir0000001390	mmu-miR-6958-5p:MIMAT0027816	9.810419	9.242883386	11.25396	11.13894
RB_p_mrmir0000001029	mmu-miR-6959-3p:MIMAT0027819	8.796313	5.945616885	6.838814	7.726039
RB_p_mrmir0000002032	mmu-miR-6959-5p:MIMAT0027818	8.970208	8.757166004	9.3186	6.395246
RB_p_mrmir0000001224	mmu-miR-696:MIMAT0003483	8.404543	7.146175309	7.838508	7.861645
RB_p_mrmir000000340	mmu-miR-6960-3p:MIMAT0027821	8.19907	7.602551855	7.708813	5.606074
RB_p_mrmir000000714	mmu-miR-6960-5p:MIMAT0027820	8.611844	4.242332974	8.286156	9.014371
RB_p_mrmir0000001725	mmu-miR-6961-3p:MIMAT0027823	6.280753	-0.09673753	6.968814	3.319464
RB_p_mrmir0000001867	mmu-miR-6961-5p:MIMAT0027822	9.233365	8.054380536	10.15429	8.799623
RB_p_mrmir0000001579	mmu-miR-6962-3p:MIMAT0027825	6.605904	7.254326996	7.54508	6.519782
RB_p_mrmir00000092	mmu-miR-6962-5p:MIMAT0027824	8.354156	8.530431945	8.070503	7.508007
RB_p_mrmir0000001953	mmu-miR-6963-3p:MIMAT0027827	8.193302	7.564097176	7.219279	7.903206
RB_p_mrmir0000001327	mmu-miR-6963-5p:MIMAT0027826	10.46132	8.144585083	9.685115	9.294956
RB_p_mrmir0000002297	mmu-miR-6964-3p:MIMAT0027829	4.467959	0.455546046	4.59214	1.535307
RB_p_mrmir0000002266	mmu-miR-6964-5p:MIMAT0027828	6.028395	7.508783547	8.051741	8.376779
RB_p_mrmir0000001822	mmu-miR-6965-3p:MIMAT0027831	6.027124	6.656379653	7.453649	6.086364
RB_p_mrmir000000315	mmu-miR-6965-5p:MIMAT0027830	12.89539	12.66086729	12.88102	13.18656
RB_p_mrmir0000001976	mmu-miR-6966-3p:MIMAT0027835	6.399477	2.142361162	8.043216	7.250015
RB_p_mrmir0000001012	mmu-miR-6966-5p:MIMAT0027834	9.122827	7.581122141	5.32063	8.459825
RB_p_mrmir0000001683	mmu-miR-6967-3p:MIMAT0027837	7.32336	5.849969815	7.061625	1.659772
RB_p_mrmir000000726	mmu-miR-6967-5p:MIMAT0027836	8.277373	8.140749052	7.289979	8.529538
RB_p_mrmir000000158	mmu-miR-6968-3p:MIMAT0027839	7.330679	8.221005077	7.387996	8.024529
RB_p_mrmir000000435	mmu-miR-6968-5p:MIMAT0027838	11.71998	9.817885907	9.418272	9.7031
RB_p_mrmir0000002159	mmu-miR-6969-3p:MIMAT0027841	4.945635	3.422855381	3.670612	3.397786
RB_p_mrmir000000695	mmu-miR-6969-5p:MIMAT0027840	9.956213	9.031396079	9.955423	8.716553
RB_p_mrmir00000047	mmu-miR-697:MIMAT0003487	5.262127	6.63738187	6.96204	6.522245
RB_p_mrmir0000001641	mmu-miR-6970-3p:MIMAT0027843	8.023175	6.612071884	7.548893	5.856366
RB_p_mrmir0000001337	mmu-miR-6970-5p:MIMAT0027842	13.5839	13.80937061	12.59161	12.19292
RB_p_mrmir000000176	mmu-miR-6971-3p:MIMAT0027845	4.067838	5.466801126	7.001191	2.483862

RB_p_mrmir0000002051	mmu-miR-6971-5p:MIMAT0027844	12.11875	12.28870226	11.97324	11.33632
RB_p_mrmir0000002173	mmu-miR-6972-3p:MIMAT0027847	5.653854	6.645818991	5.030929	1.434436
RB_p_mrmir0000001242	mmu-miR-6972-5p:MIMAT0027846	10.76461	10.30032087	11.81002	11.52714
RB_p_mrmir000000681	mmu-miR-6973a-3p:MIMAT0027849	7.886714	5.337478318	5.023926	6.685319
RB_p_mrmir0000001478	mmu-miR-6973a-5p:MIMAT0027848	12.2386	10.58012484	12.3027	10.99064
RB_p_mrmir0000001966	mmu-miR-6973b-3p:MIMAT0027909	5.090489	4.314174528	7.48571	3.661842
RB_p_mrmir0000001344	mmu-miR-6973b-5p:MIMAT0027908	10.25943	8.641158266	11.85512	6.854386
RB_p_mrmir0000001767	mmu-miR-6974-3p:MIMAT0027851	3.171776	3.140308005	7.612824	1.671642
RB_p_mrmir0000001368	mmu-miR-6974-5p:MIMAT0027850	12.07341	10.01507874	11.37921	10.92063
RB_p_mrmir0000001780	mmu-miR-6975-3p:MIMAT0027853	2.461004	5.987324761	5.577886	3.221423
RB_p_mrmir0000001245	mmu-miR-6975-5p:MIMAT0027852	11.3297	11.37324043	12.20704	11.45896
RB_p_mrmir000000555	mmu-miR-6976-3p:MIMAT0027855	5.194794	8.119929396	6.701505	6.709753
RB_p_mrmir000000725	mmu-miR-6976-5p:MIMAT0027854	7.727084	4.342241431	8.803179	8.583872
RB_p_mrmir00000097	mmu-miR-6977-3p:MIMAT0027857	7.369966	5.355926865	5.489183	6.705408
RB_p_mrmir0000001369	mmu-miR-6977-5p:MIMAT0027856	9.617552	7.38459886	8.588345	8.547609
RB_p_mrmir000000223	mmu-miR-6978-3p:MIMAT0027859	7.360074	6.0402526	7.001174	5.097624
RB_p_mrmir000000105	mmu-miR-6978-5p:MIMAT0027858	12.42889	11.49953051	11.91627	12.01241
RB_p_mrmir0000002279	mmu-miR-6979-3p:MIMAT0027861	8.041283	4.027948071	8.363793	5.204937
RB_p_mrmir0000001295	mmu-miR-6979-5p:MIMAT0027860	8.921512	8.206198052	9.177819	8.656853
RB_p_mrmir0000001870	mmu-miR-6980-3p:MIMAT0027863	6.344693	4.889217035	7.410683	6.949071
RB_p_mrmir0000001393	mmu-miR-6980-5p:MIMAT0027862	11.62673	9.833415745	10.56437	10.40029
RB_p_mrmir000000629	mmu-miR-6981-3p:MIMAT0027865	7.525103	8.220515692	8.432661	2.887352
RB_p_mrmir0000001367	mmu-miR-6981-5p:MIMAT0027864	13.14196	12.23238764	13.51583	13.41201
RB_p_mrmir0000002016	mmu-miR-6982-3p:MIMAT0027867	7.314199	3.082218616	2.896872	2.049266
RB_p_mrmir0000001046	mmu-miR-6982-5p:MIMAT0027866	12.14048	9.913847492	11.67441	10.93503
RB_p_mrmir0000001849	mmu-miR-6983-3p:MIMAT0027869	6.866427	4.887062471	6.566288	6.622183
RB_p_mrmir0000002245	mmu-miR-6983-5p:MIMAT0027868	7.282286	7.674659571	4.060029	2.846879
RB_p_mrmir000000793	mmu-miR-698-3p:MIMAT0003488	4.782238	3.279555276	5.895431	7.340826
RB_p_mrmir0000001498	mmu-miR-6984-3p:MIMAT0027871	6.386513	3.187277756	4.475075	5.328857
RB_p_mrmir000000250	mmu-miR-6984-5p:MIMAT0027870	13.92891	14.95459337	15.47017	15.53181
RB_p_mrmir000000991	mmu-miR-6985-3p:MIMAT0027873	7.398787	6.666227107	7.217266	7.998496
RB_p_mrmir0000001493	mmu-miR-6985-5p:MIMAT0027872	8.661128	8.031657328	8.162766	8.474951
RB_p_mrmir0000002139	mmu-miR-698-5p:MIMAT0022930	10.68414	8.455996239	9.309094	9.882413
RB_p_mrmir0000001408	mmu-miR-6986-3p:MIMAT0027875	6.095523	7.733907731	4.720882	7.770576
RB_p_mrmir000000430	mmu-miR-6986-5p:MIMAT0027874	7.840307	7.584476104	7.643376	7.24041
RB_p_mrmir000000672	mmu-miR-6987-3p:MIMAT0027877	6.70993	7.735939844	8.210613	6.189021
RB_p_mrmir0000002036	mmu-miR-6987-5p:MIMAT0027876	13.42943	11.02249743	13.07276	12.60661
RB_p_mrmir0000001859	mmu-miR-6988-3p:MIMAT0027879	6.832058	6.381972566	7.832593	4.532659
RB_p_mrmir0000001307	mmu-miR-6988-5p:MIMAT0027878	9.230449	7.592577239	8.678372	8.481326
RB_p_mrmir0000002100	mmu-miR-6989-3p:MIMAT0027881	7.214257	6.957218221	7.833932	1.676567
RB_p_mrmir0000002260	mmu-miR-6989-5p:MIMAT0027880	11.28662	10.42729525	12.42566	11.72784

RB_p_mrmir000000345	mmu-miR-6990-3p:MIMAT0027883	4.950016	2.12979742	6.901521	4.276607
RB_p_mrmir000000836	mmu-miR-6990-5p:MIMAT0027882	9.347695	7.035160158	7.417312	8.150838
RB_p_mrmir0000001187	mmu-miR-6991-3p:MIMAT0027885	5.52226	6.500248271	5.084713	5.561748
RB_p_mrmir0000001996	mmu-miR-6991-5p:MIMAT0027884	7.489534	10.22078066	10.7162	9.603292
RB_p_mrmir0000001762	mmu-miR-6992-3p:MIMAT0027887	5.064268	7.42962161	4.245129	2.299682
RB_p_mrmir0000001955	mmu-miR-6992-5p:MIMAT0027886	6.572153	6.483802781	5.668103	7.655624
RB_p_mrmir0000001095	mmu-miR-6993-3p:MIMAT0027889	6.794236	8.596691951	8.012411	6.862552
RB_p_mrmir000000482	mmu-miR-6993-5p:MIMAT0027888	10.89766	9.747689376	10.108	9.976858
RB_p_mrmir00000059	mmu-miR-6994-3p:MIMAT0027891	6.882966	4.443992844	4.377881	3.784123
RB_p_mrmir000000152	mmu-miR-6994-5p:MIMAT0027890	9.768761	8.337987558	10.26044	9.322378
RB_p_mrmir0000002142	mmu-miR-6995-3p:MIMAT0027893	6.129796	3.68139404	5.692704	4.435031
RB_p_mrmir0000001058	mmu-miR-6995-5p:MIMAT0027892	13.43802	12.47702836	11.58605	11.79043
RB_p_mrmir000000951	mmu-miR-6996-3p:MIMAT0027895	7.081245	2.533742872	5.517703	3.420368
RB_p_mrmir0000001928	mmu-miR-6996-5p:MIMAT0027894	9.827464	7.172082838	9.370992	9.69457
RB_p_mrmir0000001623	mmu-miR-6997-3p:MIMAT0027897	7.380162	6.524342771	6.327811	4.265225
RB_p_mrmir0000001417	mmu-miR-6997-5p:MIMAT0027896	13.58694	12.9779967	12.2362	12.35395
RB_p_mrmir000000306	mmu-miR-6998-3p:MIMAT0027899	5.21568	7.160337885	7.391819	7.5964
RB_p_mrmir0000001060	mmu-miR-6998-5p:MIMAT0027898	9.95057	9.824802795	9.889123	9.528895
RB_p_mrmir0000001091	mmu-miR-6999-3p:MIMAT0027901	6.719679	8.435944349	8.105911	5.098375
RB_p_mrmir00000091	mmu-miR-6999-5p:MIMAT0027900	10.98846	9.494900348	10.66094	12.08075
RB_p_mrmir000000671	mmu-miR-7000-3p:MIMAT0027903	7.758498	7.231951801	6.958075	6.238903
RB_p_mrmir000000429	mmu-miR-7000-5p:MIMAT0027902	8.19898	8.780819823	7.424765	6.599915
RB_p_mrmir000000921	mmu-miR-7001-3p:MIMAT0027905	6.403268	5.869053039	5.871418	2.749877
RB_p_mrmir000000392	mmu-miR-7001-5p:MIMAT0027904	9.291402	8.500638971	10.15539	9.594904
RB_p_mrmir0000002276	mmu-miR-7002-3p:MIMAT0027907	5.955875	6.694347452	6.042688	5.254961
RB_p_mrmir0000002255	mmu-miR-7002-5p:MIMAT0027906	6.778622	6.315770901	8.837828	7.463545
RB_p_mrmir000000845	mmu-miR-7003-3p:MIMAT0027911	8.301431	3.547746355	6.905833	6.401385
RB_p_mrmir0000002138	mmu-miR-7003-5p:MIMAT0027910	8.778554	9.442757896	9.264057	8.194373
RB_p_mrmir000000674	mmu-miR-700-3p:MIMAT0003490	6.883838	6.466526658	7.824772	8.30785
RB_p_mrmir000000832	mmu-miR-7004-3p:MIMAT0027913	7.13174	6.414923432	5.19577	4.982239
RB_p_mrmir0000002207	mmu-miR-7004-5p:MIMAT0027912	7.005714	7.730690087	7.74751	4.929203
RB_p_mrmir0000001077	mmu-miR-7005-3p:MIMAT0027915	7.304989	5.160036008	8.00989	6.950892
RB_p_mrmir000000889	mmu-miR-7005-5p:MIMAT0027914	13.54454	13.48339742	13.38829	13.47743
RB_p_mrmir0000001431	mmu-miR-700-5p:MIMAT0017256	6.724848	4.564254584	7.376338	5.720282
RB_p_mrmir0000002294	mmu-miR-7006-3p:MIMAT0027917	5.344202	1.637346974	6.428828	3.988727
RB_p_mrmir0000002054	mmu-miR-7006-5p:MIMAT0027916	7.207368	8.557919832	8.390999	5.624364
RB_p_mrmir000000841	mmu-miR-7007-3p:MIMAT0027919	6.260623	4.479274116	3.004018	7.247737
RB_p_mrmir0000001649	mmu-miR-7007-5p:MIMAT0027918	14.01421	12.93124776	14.39584	13.59252
RB_p_mrmir0000002126	mmu-miR-7008-3p:MIMAT0027921	9.048403	6.312296104	8.073604	6.025877
RB_p_mrmir0000002133	mmu-miR-7008-5p:MIMAT0027920	10.96679	9.726450959	10.4181	10.36506
RB_p_mrmir0000001832	mmu-miR-7009-3p:MIMAT0027923	5.286659	9.698607626	7.584575	6.842551

RB_p_mrmir0000002265	mmu-miR-7009-5p:MIMAT0027922	10.91386	10.46467395	10.20677	9.698672
RB_p_mrmir000000452	mmu-miR-7010-3p:MIMAT0027925	4.932877	5.922463512	3.37781	2.823021
RB_p_mrmir000000923	mmu-miR-7010-5p:MIMAT0027924	6.502214	4.210719061	6.915301	7.831635
RB_p_mrmir0000001801	mmu-miR-7011-3p:MIMAT0027927	7.804859	8.910146942	4.312341	8.691556
RB_p_mrmir000000380	mmu-miR-7011-5p:MIMAT0027926	14.44283	14.44377921	15.17655	14.95305
RB_p_mrmir0000001860	mmu-miR-7012-3p:MIMAT0027929	4.954039	6.837109409	7.53967	6.464908
RB_p_mrmir00000093	mmu-miR-7012-5p:MIMAT0027928	13.0182	11.18049712	12.17225	12.28984
RB_p_mrmir000000805	mmu-miR-7013-3p:MIMAT0027931	8.343694	7.291713911	6.505251	7.765269
RB_p_mrmir0000001580	mmu-miR-7013-5p:MIMAT0027930	7.448334	3.067184516	5.780969	1.30393
RB_p_mrmir0000001578	mmu-miR-701-3p:MIMAT0017257	2.190199	5.581365502	6.217123	2.877878
RB_p_mrmir000000879	mmu-miR-7014-3p:MIMAT0027933	7.500614	4.581143465	6.928562	5.671353
RB_p_mrmir0000002267	mmu-miR-7014-5p:MIMAT0027932	11.82233	10.29396707	13.04288	12.35164
RB_p_mrmir0000001763	mmu-miR-7015-3p:MIMAT0027935	6.826656	4.554077706	6.254304	4.380109
RB_p_mrmir0000001821	mmu-miR-7015-5p:MIMAT0027934	9.966629	6.745402199	7.827182	8.36048
RB_p_mrmir0000002176	mmu-miR-701-5p:MIMAT0003491	6.227762	5.624939583	5.937928	4.553357
RB_p_mrmir000000210	mmu-miR-7016-3p:MIMAT0027937	7.596722	9.493420089	6.520033	7.841494
RB_p_mrmir000000727	mmu-miR-7016-5p:MIMAT0027936	13.80986	12.55353598	12.74413	12.26394
RB_p_mrmir000000202	mmu-miR-7017-3p:MIMAT0027939	7.54557	6.633889918	6.824654	3.599621
RB_p_mrmir000000318	mmu-miR-7017-5p:MIMAT0027938	8.743167	8.464732301	7.820276	8.371733
RB_p_mrmir0000001636	mmu-miR-7018-3p:MIMAT0027941	6.992313	8.235612427	4.058471	3.705786
RB_p_mrmir0000001364	mmu-miR-7018-5p:MIMAT0027940	12.85936	11.3293351	13.09744	12.7764
RB_p_mrmir0000001640	mmu-miR-7019-3p:MIMAT0027943	7.117504	7.323546043	7.855645	5.467685
RB_p_mrmir000000558	mmu-miR-7019-5p:MIMAT0027942	11.75512	11.26724638	10.44619	10.20341
RB_p_mrmir00000053	mmu-miR-7020-3p:MIMAT0027945	5.720563	2.496129568	6.014474	5.014808
RB_p_mrmir0000002040	mmu-miR-7020-5p:MIMAT0027944	12.33859	12.84648205	13.3816	12.0693
RB_p_mrmir0000001783	mmu-miR-7021-3p:MIMAT0027947	5.946792	6.087787978	6.987315	6.537032
RB_p_mrmir000000524	mmu-miR-7021-5p:MIMAT0027946	8.391042	7.040702889	7.342266	8.274343
RB_p_mrmir000000157	mmu-miR-7022-3p:MIMAT0027949	8.348319	8.741017299	8.3831	8.189465
RB_p_mrmir000000888	mmu-miR-7022-5p:MIMAT0027948	10.17614	5.942209098	9.704842	9.781668
RB_p_mrmir0000001638	mmu-miR-7023-3p:MIMAT0027951	5.882694	8.316336299	6.603116	6.701895
RB_p_mrmir000000575	mmu-miR-7023-5p:MIMAT0027950	13.04283	11.87677149	12.13092	12.17491
RB_p_mrmir0000001945	mmu-miR-702-3p:MIMAT0003492	6.573118	9.061049906	4.707975	5.127221
RB_p_mrmir000000813	mmu-miR-7024-3p:MIMAT0027953	7.177792	6.284345319	7.191423	6.186065
RB_p_mrmir0000002263	mmu-miR-7024-5p:MIMAT0027952	7.849017	6.974706799	6.603736	5.390563
RB_p_mrmir000000693	mmu-miR-7025-3p:MIMAT0027955	7.553301	8.242848461	6.854389	8.18125
RB_p_mrmir000000959	mmu-miR-7025-5p:MIMAT0027954	8.127117	8.625719498	6.616205	4.637179
RB_p_mrmir0000001376	mmu-miR-702-5p:MIMAT0022931	8.730265	6.690088947	8.199564	3.772613
RB_p_mrmir0000002127	mmu-miR-7026-3p:MIMAT0027957	7.076558	8.472286681	7.557125	4.165805
RB_p_mrmir0000002219	mmu-miR-7026-5p:MIMAT0027956	4.558823	3.519983354	5.09641	1.034057
RB_p_mrmir0000001748	mmu-miR-7027-3p:MIMAT0027959	7.607461	5.364110264	6.055849	7.598291
RB_p_mrmir0000001981	mmu-miR-7027-5p:MIMAT0027958	10.23107	10.20973501	12.093	11.59124

RB_p_mrmir000000899	mmu-miR-7028-3p:MIMAT0027961	6.926829	6.540829503	8.351129	5.699203
RB_p_mrmir0000002043	mmu-miR-7028-5p:MIMAT0027960	10.61322	8.500882833	10.45538	9.597304
RB_p_mrmir0000002131	mmu-miR-7029-3p:MIMAT0027963	5.433291	5.583943543	6.891016	7.161027
RB_p_mrmir0000001803	mmu-miR-7029-5p:MIMAT0027962	7.419287	7.604766839	6.41467	4.977314
RB_p_mrmir0000002	mmu-miR-703:MIMAT0003493	10.42009	8.628367293	11.32744	10.49966
RB_p_mrmir0000001096	mmu-miR-7030-3p:MIMAT0027965	4.751536	0.74305234	6.766436	3.589307
RB_p_mrmir0000002048	mmu-miR-7030-5p:MIMAT0027964	9.009497	8.310043947	8.288355	8.75534
RB_p_mrmir00000056	mmu-miR-7031-3p:MIMAT0027967	6.598259	6.93236108	5.537217	5.34681
RB_p_mrmir000000883	mmu-miR-7031-5p:MIMAT0027966	12.68934	11.69388512	13.29533	8.277702
RB_p_mrmir000000549	mmu-miR-7032-3p:MIMAT0027969	5.888984	5.506468703	7.080541	2.114773
RB_p_mrmir000000837	mmu-miR-7032-5p:MIMAT0027968	10.6779	9.205905348	10.85404	9.076415
RB_p_mrmir0000001952	mmu-miR-7033-3p:MIMAT0027971	6.912379	3.830767244	6.231548	3.695657
RB_p_mrmir0000001768	mmu-miR-7033-5p:MIMAT0027970	13.82054	13.63902587	13.15014	13.09209
RB_p_mrmir0000002254	mmu-miR-7034-3p:MIMAT0027973	7.964553	6.14994455	7.13492	6.034349
RB_p_mrmir0000001716	mmu-miR-7034-5p:MIMAT0027972	9.519528	7.704505483	9.432219	8.938833
RB_p_mrmir0000001790	mmu-miR-7035-3p:MIMAT0027975	5.954212	3.904958993	7.877525	6.418926
RB_p_mrmir0000001282	mmu-miR-7035-5p:MIMAT0027974	10.36675	9.964730324	9.949945	9.947295
RB_p_mrmir000000865	mmu-miR-7036a-3p:MIMAT0027977	7.377966	2.371617481	7.520188	4.281429
RB_p_mrmir000000352	mmu-miR-7036a-5p:MIMAT0027976	13.3412	10.30884296	12.60351	11.68145
RB_p_mrmir0000002213	mmu-miR-7036b-3p:MIMAT0029809	10.22866	7.390902353	8.076149	7.969097
RB_p_mrmir000000219	mmu-miR-7036b-5p:MIMAT0029808	8.051605	6.954670083	8.139295	8.024928
RB_p_mrmir0000002108	mmu-miR-7037-3p:MIMAT0027979	6.554687	6.110391771	7.205845	4.223065
RB_p_mrmir000000107	mmu-miR-7037-5p:MIMAT0027978	7.42657	8.024789996	7.469215	5.964074
RB_p_mrmir000000685	mmu-miR-7038-3p:MIMAT0027981	7.853318	4.627553465	6.32722	7.082228
RB_p_mrmir0000002087	mmu-miR-7038-5p:MIMAT0027980	9.211468	8.371862552	10.65921	8.594076
RB_p_mrmir000000894	mmu-miR-7039-3p:MIMAT0027983	4.146294	4.528429636	8.559197	5.052102
RB_p_mrmir000000316	mmu-miR-7039-5p:MIMAT0027982	9.255057	6.631618123	9.368393	9.483978
RB_p_mrmir000000296	mmu-miR-704:MIMAT0003494	5.436459	1.497373854	6.474547	2.45367
RB_p_mrmir000000916	mmu-miR-7040-3p:MIMAT0027985	7.283104	6.999306166	5.060784	6.073118
RB_p_mrmir000000764	mmu-miR-7040-5p:MIMAT0027984	14.98586	14.99360571	15.39657	15.04142
RB_p_mrmir0000002073	mmu-miR-7041-3p:MIMAT0027987	5.226107	5.309131863	4.237248	6.58706
RB_p_mrmir000000314	mmu-miR-7041-5p:MIMAT0027986	7.88004	8.459885657	6.983032	4.763885
RB_p_mrmir0000002102	mmu-miR-7042-3p:MIMAT0027989	4.234137	3.304489621	6.675349	5.907277
RB_p_mrmir0000001501	mmu-miR-7042-5p:MIMAT0027988	11.05703	11.30388754	11.56087	12.5293
RB_p_mrmir000000279	mmu-miR-7043-3p:MIMAT0027991	6.299154	9.180717999	8.757689	7.151925
RB_p_mrmir0000002116	mmu-miR-7043-5p:MIMAT0027990	8.867154	7.347247342	8.557737	9.480153
RB_p_mrmir000000566	mmu-miR-7044-3p:MIMAT0027993	8.11851	6.559393146	6.263395	5.667173
RB_p_mrmir0000001398	mmu-miR-7044-5p:MIMAT0027992	12.20318	12.34696431	11.78955	12.82162
RB_p_mrmir0000001771	mmu-miR-7045-3p:MIMAT0027995	8.540916	8.004730444	8.392243	7.938071
RB_p_mrmir000000353	mmu-miR-7045-5p:MIMAT0027994	15.5311	15.32823256	14.67537	15.57722
RB_p_mrmir0000001841	mmu-miR-7046-3p:MIMAT0027997	5.00302	7.796474114	9.477541	3.314002

RB_p_mrmir0000002092	mmu-miR-7046-5p:MIMAT0027996	13.00516	11.0661574	13.10498	12.61756
RB_p_mrmir0000001635	mmu-miR-7047-3p:MIMAT0027999	6.76566	3.48730787	5.163892	6.057972
RB_p_mrmir0000001889	mmu-miR-7047-5p:MIMAT0027998	12.92	9.660336562	12.21161	12.3781
RB_p_mrmir0000001825	mmu-miR-7048-3p:MIMAT0028001	5.569602	5.900466663	7.831854	4.084025
RB_p_mrmir000000941	mmu-miR-7048-5p:MIMAT0028000	12.97992	13.32240363	13.65573	13.90338
RB_p_mrmir0000001934	mmu-miR-7049-3p:MIMAT0028003	8.733504	6.302772749	7.203098	4.43258
RB_p_mrmir0000001788	mmu-miR-7049-5p:MIMAT0028002	10.33411	9.220354462	10.89377	9.871671
RB_p_mrmir0000001326	mmu-miR-705:MIMAT0003495	12.77554	12.48148297	11.68467	11.90286
RB_p_mrmir0000001765	mmu-miR-7050-3p:MIMAT0028005	6.958158	7.535314969	5.939025	6.285731
RB_p_mrmir000000177	mmu-miR-7050-5p:MIMAT0028004	13.6779	13.22116996	13.91402	13.85569
RB_p_mrmir0000001881	mmu-miR-7051-3p:MIMAT0028007	6.027512	2.128428582	7.79295	7.10089
RB_p_mrmir0000001632	mmu-miR-7051-5p:MIMAT0028006	9.077592	7.00686254	8.750747	8.147878
RB_p_mrmir0000001234	mmu-miR-7052-3p:MIMAT0028009	6.501234	6.728955538	6.889093	5.765343
RB_p_mrmir0000002132	mmu-miR-7052-5p:MIMAT0028008	10.90697	9.890902462	10.87905	10.66641
RB_p_mrmir0000001009	mmu-miR-7053-3p:MIMAT0028011	4.686607	1.975350848	7.847352	5.862392
RB_p_mrmir0000002045	mmu-miR-7053-5p:MIMAT0028010	9.592114	8.991679848	7.712271	8.368758
RB_p_mrmir0000001689	mmu-miR-7054-3p:MIMAT0028013	4.545355	6.128554721	7.36331	2.796883
RB_p_mrmir0000001534	mmu-miR-7054-5p:MIMAT0028012	9.817796	6.836812057	8.884528	8.782469
RB_p_mrmir0000002243	mmu-miR-7055-3p:MIMAT0028015	7.563851	6.598588034	6.059898	5.991459
RB_p_mrmir0000001676	mmu-miR-7055-5p:MIMAT0028014	4.643158	6.41949158	6.470269	2.771214
RB_p_mrmir000000874	mmu-miR-7056-3p:MIMAT0028017	7.585314	7.778227882	6.764642	6.962568
RB_p_mrmir0000002130	mmu-miR-7056-5p:MIMAT0028016	12.6786	10.36596463	13.13925	12.47748
RB_p_mrmir0000001451	mmu-miR-7057-3p:MIMAT0028019	6.887023	4.687219471	6.32227	8.186991
RB_p_mrmir000000708	mmu-miR-7057-5p:MIMAT0028018	7.850663	6.183738629	7.820975	4.875438
RB_p_mrmir0000001013	mmu-miR-7058-3p:MIMAT0028021	4.307559	2.387602683	3.777847	5.514343
RB_p_mrmir0000001503	mmu-miR-7058-5p:MIMAT0028020	12.3865	12.20076193	12.4173	12.48736
RB_p_mrmir0000002163	mmu-miR-7059-3p:MIMAT0028023	7.019596	5.554677629	4.287844	7.677372
RB_p_mrmir000000206	mmu-miR-7059-5p:MIMAT0028022	8.452429	6.61760178	6.987736	6.880359
RB_p_mrmir000000304	mmu-miR-706:MIMAT0003496	7.814356	6.005378227	8.789673	9.387756
RB_p_mrmir0000001757	mmu-miR-7060-3p:MIMAT0028025	4.432024	6.960365967	3.909529	2.198195
RB_p_mrmir0000001374	mmu-miR-7060-5p:MIMAT0028024	10.49463	8.495361574	10.14124	9.717773
RB_p_mrmir0000001575	mmu-miR-7061-3p:MIMAT0028027	3.604733	2.313749489	5.987897	3.438783
RB_p_mrmir0000001585	mmu-miR-7061-5p:MIMAT0028026	5.345375	5.455232968	3.832071	8.19703
RB_p_mrmir000000231	mmu-miR-7062-3p:MIMAT0028029	7.113594	7.018769591	5.928027	7.231347
RB_p_mrmir0000002004	mmu-miR-7062-5p:MIMAT0028028	6.685512	7.326133183	7.485728	7.886392
RB_p_mrmir0000001965	mmu-miR-7063-3p:MIMAT0028031	6.174403	5.124721429	6.647939	4.541914
RB_p_mrmir000000564	mmu-miR-7063-5p:MIMAT0028030	10.20093	7.889431182	9.21197	9.247583
RB_p_mrmir000000729	mmu-miR-7064-3p:MIMAT0028033	7.23754	8.388937738	7.089088	2.680918
RB_p_mrmir0000001650	mmu-miR-7064-5p:MIMAT0028032	4.158565	7.451514218	3.076808	4.881
RB_p_mrmir0000002270	mmu-miR-7065-3p:MIMAT0028035	2.630207	3.598611185	5.222093	3.599953
RB_p_mrmir000000489	mmu-miR-7065-5p:MIMAT0028034	6.400664	9.984203055	6.320027	6.55059

RB_p_mrmir0000001756	mmu-miR-7066-3p:MIMAT0028037	6.962545	7.538129978	4.310012	6.793768
RB_p_mrmir0000002059	mmu-miR-7066-5p:MIMAT0028036	10.64059	8.570500778	12.10214	10.91087
RB_p_mrmir0000001926	mmu-miR-7067-3p:MIMAT0028039	6.661066	7.481595277	5.550011	5.800056
RB_p_mrmir0000001937	mmu-miR-7067-5p:MIMAT0028038	4.768861	6.868463103	7.540262	3.128375
RB_p_mrmir0000001637	mmu-miR-7068-3p:MIMAT0028043	5.325393	3.568414342	7.662102	6.025962
RB_p_mrmir0000001370	mmu-miR-7068-5p:MIMAT0028042	11.17141	10.50378732	10.75302	10.07108
RB_p_mrmir000000886	mmu-miR-7069-3p:MIMAT0028045	8.47807	7.107209722	5.559758	6.837839
RB_p_mrmir0000002264	mmu-miR-7069-5p:MIMAT0028044	10.33513	10.63415757	10.23777	9.650754
RB_p_mrmir000000737	mmu-miR-707:MIMAT0003497	7.594036	7.201430019	8.232945	4.060625
RB_p_mrmir000000723	mmu-miR-7070-3p:MIMAT0028047	6.923047	5.222674528	5.800619	3.918445
RB_p_mrmir0000001372	mmu-miR-7070-5p:MIMAT0028046	13.63378	14.0104864	13.50894	13.81951
RB_p_mrmir0000001025	mmu-miR-7071-3p:MIMAT0028049	6.964257	4.813189868	4.777272	2.156599
RB_p_mrmir000000850	mmu-miR-7071-5p:MIMAT0028048	9.305177	8.193605728	9.152573	8.636501
RB_p_mrmir000000973	mmu-miR-7072-3p:MIMAT0028051	8.001955	8.784524573	6.056795	5.633361
RB_p_mrmir0000001157	mmu-miR-7072-5p:MIMAT0028050	11.698	13.02136252	13.59659	13.22978
RB_p_mrmir000000523	mmu-miR-7073-3p:MIMAT0028053	7.578278	7.834882538	9.009019	7.730016
RB_p_mrmir0000002050	mmu-miR-7073-5p:MIMAT0028052	6.389409	7.812098471	6.38103	2.622294
RB_p_mrmir0000001538	mmu-miR-7074-3p:MIMAT0028055	6.515907	4.604549084	5.246229	5.382808
RB_p_mrmir0000002035	mmu-miR-7074-5p:MIMAT0028054	8.240251	4.249360489	7.538019	5.763692
RB_p_mrmir000000631	mmu-miR-7075-3p:MIMAT0028057	3.48411	3.933939686	7.925583	2.08882
RB_p_mrmir000000937	mmu-miR-7075-5p:MIMAT0028056	9.687474	9.208709267	9.604014	8.556056
RB_p_mrmir0000001861	mmu-miR-7076-3p:MIMAT0028059	8.327077	5.931652312	5.830172	6.391092
RB_p_mrmir000000431	mmu-miR-7076-5p:MIMAT0028058	9.035469	7.393143486	8.71198	7.669551
RB_p_mrmir000000898	mmu-miR-7077-3p:MIMAT0028061	8.525253	4.997911532	7.760759	6.240215
RB_p_mrmir0000001272	mmu-miR-7077-5p:MIMAT0028060	7.597954	5.211661134	5.667293	4.61813
RB_p_mrmir0000001499	mmu-miR-7078-3p:MIMAT0028063	8.051767	3.65373632	4.716057	2.822815
RB_p_mrmir0000002140	mmu-miR-7078-5p:MIMAT0028062	9.929418	8.142859549	9.965638	8.648455
RB_p_mrmir0000001869	mmu-miR-7079-3p:MIMAT0028065	4.44161	6.751977044	3.692525	3.325388
RB_p_mrmir000000423	mmu-miR-7079-5p:MIMAT0028064	9.536273	8.494146812	8.59533	5.293643
RB_p_mrmir000000720	mmu-miR-7080-3p:MIMAT0028067	7.986624	5.266671982	5.285426	5.75468
RB_p_mrmir0000001342	mmu-miR-7080-5p:MIMAT0028066	10.29547	7.497953314	10.25477	9.811434
RB_p_mrmir0000002202	mmu-miR-7081-3p:MIMAT0028069	4.764989	3.527026812	7.547423	4.575263
RB_p_mrmir000000309	mmu-miR-7081-5p:MIMAT0028068	11.46212	9.622014411	10.33835	9.432451
RB_p_mrmir0000001201	mmu-miR-7082-3p:MIMAT0028071	8.513442	5.654728759	6.441507	6.803292
RB_p_mrmir0000001476	mmu-miR-7082-5p:MIMAT0028070	15.24334	15.50860244	15.36448	15.49942
RB_p_mrmir0000001800	mmu-miR-7083-3p:MIMAT0028073	6.60384	6.879618637	6.176715	5.736115
RB_p_mrmir0000001746	mmu-miR-7083-5p:MIMAT0028072	6.72749	7.73513272	10.67429	11.2508
RB_p_mrmir000000637	mmu-miR-708-3p:MIMAT0003498	4.467569	5.916631337	4.931646	6.784631
RB_p_mrmir0000001209	mmu-miR-7084-3p:MIMAT0028075	7.959773	5.807673009	5.613004	7.515127
RB_p_mrmir0000001502	mmu-miR-7084-5p:MIMAT0028074	11.01668	9.051595705	12.02688	11.90177
RB_p_mrmir0000001526	mmu-miR-7085-3p:MIMAT0028077	8.029043	6.252583068	6.497853	6.08081

RB_p_mrmir000000750	mmu-miR-7085-5p:MIMAT0028076	13.22361	11.97630252	12.07864	12.1194
RB_p_mrmir00000095	mmu-miR-708-5p:MIMAT0004828	7.169813	5.668583365	6.431537	3.300212
RB_p_mrmir0000001722	mmu-miR-7086-3p:MIMAT0028079	5.298828	2.423083827	7.898559	4.456057
RB_p_mrmir00000078	mmu-miR-7086-5p:MIMAT0028078	8.431885	7.896189458	9.395651	9.215212
RB_p_mrmir0000001974	mmu-miR-7087-3p:MIMAT0028081	2.310918	4.179501567	8.163535	4.983345
RB_p_mrmir000000393	mmu-miR-7087-5p:MIMAT0028080	9.182639	7.226506566	7.44466	2.368617
RB_p_mrmir0000002233	mmu-miR-7088-3p:MIMAT0028083	6.533027	3.937280266	5.582756	5.064443
RB_p_mrmir0000001178	mmu-miR-7088-5p:MIMAT0028082	12.96963	11.26499741	12.85437	12.15651
RB_p_mrmir0000001677	mmu-miR-7089-3p:MIMAT0028085	6.750747	4.987204586	6.274986	6.808789
RB_p_mrmir000000868	mmu-miR-7089-5p:MIMAT0028084	6.962316	6.273383701	8.396309	6.747413
RB_p_mrmir0000001262	mmu-miR-709:MIMAT0003499	14.10311	13.72484102	14.62521	14.50757
RB_p_mrmir0000002302	mmu-miR-7090-3p:MIMAT0028087	4.750543	3.013478214	5.703912	1.764329
RB_p_mrmir0000001764	mmu-miR-7090-5p:MIMAT0028086	6.66326	4.599459014	5.909016	5.641777
RB_p_mrmir000000481	mmu-miR-7091-3p:MIMAT0028089	7.225161	9.108161158	6.654972	1.500915
RB_p_mrmir0000001343	mmu-miR-7091-5p:MIMAT0028088	5.918483	8.305181822	7.608457	4.209274
RB_p_mrmir0000002157	mmu-miR-7092-3p:MIMAT0028091	5.832718	3.005634249	7.782087	7.010715
RB_p_mrmir00000018	mmu-miR-7092-5p:MIMAT0028090	8.163693	6.818728814	7.098764	2.972888
RB_p_mrmir0000002287	mmu-miR-7093-3p:MIMAT0028093	7.859795	6.841816055	7.13402	2.118457
RB_p_mrmir000000713	mmu-miR-7093-5p:MIMAT0028092	9.546148	8.243719943	9.971672	9.314878
RB_p_mrmir0000001786	mmu-miR-7094-1-5p:MIMAT0028094	7.719905	7.234921295	7.485587	7.491474
RB_p_mrmir0000001491	mmu-miR-7094-3p:MIMAT0028095	8.499887	5.088208906	3.837798	3.91311
RB_p_mrmir0000001804	mmu-miR-7094b-2-5p:MIMAT0028096	8.338496	8.910384424	8.461331	7.862554
RB_p_mrmir000000801	mmu-miR-710:MIMAT0003500	9.795482	4.504141258	10.18992	9.629183
RB_p_mrmir0000001285	mmu-miR-711:MIMAT0003501	10.65123	8.011432384	9.771421	9.070114
RB_p_mrmir000000287	mmu-miR-7115-3p:MIMAT0028128	7.562112	7.495428237	7.423924	6.177107
RB_p_mrmir0000001747	mmu-miR-7115-5p:MIMAT0028127	7.866127	7.21196326	7.41043	6.051242
RB_p_mrmir0000002325	mmu-miR-7116-3p:MIMAT0028130	6.622034	5.00609305	4.717502	7.273837
RB_p_mrmir0000001844	mmu-miR-7116-5p:MIMAT0028129	6.417599	14.02229715	15.70438	15.75265
RB_p_mrmir0000002105	mmu-miR-7117-3p:MIMAT0028132	6.568704	9.230261207	7.373163	4.176236
RB_p_mrmir0000001815	mmu-miR-7117-5p:MIMAT0028131	7.480002	3.112247265	5.77648	6.080804
RB_p_mrmir0000001424	mmu-miR-7118-3p:MIMAT0028134	6.98426	5.907166008	6.838366	3.724553
RB_p_mrmir0000002047	mmu-miR-7118-5p:MIMAT0028133	14.43825	15.08304749	15.10083	15.42112
RB_p_mrmir0000001	mmu-miR-7119-3p:MIMAT0028136	4.678904	3.404942288	7.436194	8.598691
RB_p_mrmir0000001062	mmu-miR-7119-5p:MIMAT0028135	7.575634	4.975345146	6.524302	6.753112
RB_p_mrmir0000001957	mmu-miR-712-3p:MIMAT0003743	7.032522	8.226756084	7.499803	6.924622
RB_p_mrmir0000001010	mmu-miR-712-5p:MIMAT0003502	6.853171	2.037060303	6.743077	4.433793
RB_p_mrmir0000001930	mmu-miR-713:MIMAT0003504	9.157297	8.790654414	8.470062	10.17922
RB_p_mrmir000000906	mmu-miR-714:MIMAT0003505	9.243773	9.19788209	9.472695	9.203904
RB_p_mrmir000000999	mmu-miR-717:MIMAT0003510	5.962017	4.405619334	7.485392	4.174781
RB_p_mrmir0000001092	mmu-miR-718:MIMAT0003514	7.780733	5.374954322	4.424282	5.413155
RB_p_mrmir000000556	mmu-miR-719:MIMAT0003465	4.27874	6.444059547	7.565234	5.728501

RB_p_mrmir000000747	mmu-miR-721:MIMAT0003515	8.773383	4.256518362	8.509549	8.464717
RB_p_mrmir0000002180	mmu-miR-7210-3p:MIMAT0028389	6.824063	3.979920309	4.252216	4.501069
RB_p_mrmir0000001420	mmu-miR-7210-5p:MIMAT0028388	7.416213	8.496826465	4.115593	8.62652
RB_p_mrmir0000002005	mmu-miR-7211-3p:MIMAT0028391	12.71534	11.70000617	12.47742	12.64473
RB_p_mrmir0000001830	mmu-miR-7211-5p:MIMAT0028390	7.887777	6.650793031	7.089924	8.161106
RB_p_mrmir0000001414	mmu-miR-7212-3p:MIMAT0028393	8.189091	8.458008414	6.598017	9.085802
RB_p_mrmir0000001816	mmu-miR-7212-5p:MIMAT0028392	9.224419	7.39166991	9.051527	8.363542
RB_p_mrmir0000001474	mmu-miR-7213-3p:MIMAT0028395	7.086316	6.762367871	6.169864	7.188742
RB_p_mrmir000000277	mmu-miR-7213-5p:MIMAT0028394	4.609382	4.541054757	6.761253	5.200194
RB_p_mrmir0000001001	mmu-miR-7214-3p:MIMAT0028397	7.5373	8.561705354	7.296516	6.793808
RB_p_mrmir0000002164	mmu-miR-7214-5p:MIMAT0028396	4.922115	2.565478481	7.58376	6.582648
RB_p_mrmir0000001967	mmu-miR-7215-3p:MIMAT0028399	11.22362	9.643179168	10.78236	9.224541
RB_p_mrmir0000001235	mmu-miR-7215-5p:MIMAT0028398	5.295916	2.579382622	6.395576	2.314522
RB_p_mrmir0000001993	mmu-miR-7216-3p:MIMAT0028401	7.485964	6.008664518	8.244343	5.929418
RB_p_mrmir0000002001	mmu-miR-7216-5p:MIMAT0028400	10.68817	10.10568412	10.70627	10.73011
RB_p_mrmir0000001871	mmu-miR-7217-3p:MIMAT0028403	10.58359	10.00637311	11.11822	10.69324
RB_p_mrmir00000073	mmu-miR-7217-5p:MIMAT0028402	11.27168	9.987033772	12.1301	11.74793
RB_p_mrmir000000965	mmu-miR-7218-3p:MIMAT0028405	7.814477	4.548329103	6.257176	5.654059
RB_p_mrmir0000001938	mmu-miR-7218-5p:MIMAT0028404	10.75928	8.811703149	10.98636	10.31308
RB_p_mrmir0000001576	mmu-miR-7219-3p:MIMAT0028407	7.445637	7.162004254	5.11828	6.792904
RB_p_mrmir0000002150	mmu-miR-7219-5p:MIMAT0028406	7.083786	3.301165036	6.99408	5.60887
RB_p_mrmir000000816	mmu-miR-7220-3p:MIMAT0028409	6.977952	7.043585454	4.795991	5.211408
RB_p_mrmir0000001321	mmu-miR-7220-5p:MIMAT0028408	1.658735	7.166201171	3.745505	0.563412
RB_p_mrmir0000001868	mmu-miR-7221-3p:MIMAT0028411	13.61716	13.0516077	12.89729	13.40231
RB_p_mrmir000000956	mmu-miR-7221-5p:MIMAT0028410	9.793736	8.82912575	7.247221	7.649177
RB_p_mrmir0000001693	mmu-miR-7222-3p:MIMAT0028413	13.19278	13.23482298	13.43626	11.58506
RB_p_mrmir0000001270	mmu-miR-7222-5p:MIMAT0028412	7.656628	8.981366853	5.479905	7.189574
RB_p_mrmir0000001354	mmu-miR-7223-3p:MIMAT0028415	7.937426	2.573429627	4.658871	3.846124
RB_p_mrmir0000001531	mmu-miR-7223-5p:MIMAT0028414	8.195805	6.701706257	8.520589	7.73407
RB_p_mrmir0000001690	mmu-miR-7224-3p:MIMAT0028417	9.475127	10.84028119	8.829116	10.16289
RB_p_mrmir0000001310	mmu-miR-7224-5p:MIMAT0028416	6.736506	6.360843982	6.681714	6.823155
RB_p_mrmir0000001093	mmu-miR-7225-3p:MIMAT0028419	8.354385	4.058214676	6.601021	4.6246
RB_p_mrmir000000228	mmu-miR-7225-5p:MIMAT0028418	5.610819	7.284004585	8.104714	7.227571
RB_p_mrmir0000001852	mmu-miR-7226-3p:MIMAT0028421	8.404538	3.845088734	7.527462	5.51286
RB_p_mrmir0000001196	mmu-miR-7226-5p:MIMAT0028420	11.10441	8.837765	11.31347	10.65669
RB_p_mrmir0000001785	mmu-miR-7227-3p:MIMAT0028423	7.714261	7.456616807	7.301702	8.337727
RB_p_mrmir0000001797	mmu-miR-7227-5p:MIMAT0028422	3.216339	3.357311241	5.453346	2.281158
RB_p_mrmir000000491	mmu-miR-7228-3p:MIMAT0028425	3.763693	6.326833898	7.270269	1.937416
RB_p_mrmir0000002019	mmu-miR-7228-5p:MIMAT0028424	6.115054	7.51497866	5.980705	4.194554
RB_p_mrmir0000001455	mmu-miR-7229-3p:MIMAT0028427	6.961847	5.174442878	6.234748	7.582293
RB_p_mrmir0000001552	mmu-miR-7229-5p:MIMAT0028426	4.226013	2.218338643	5.99642	5.184423

RB_p_mrmir0000002235	mmu-miR-7230-3p:MIMAT0028429	6.433606	7.011590168	5.223154	3.368807
RB_p_mrmir000000302	mmu-miR-7230-5p:MIMAT0028428	7.613273	6.194518244	7.51294	5.958404
RB_p_mrmir0000001097	mmu-miR-7231-3p:MIMAT0028431	7.651679	10.20128828	8.42495	9.290901
RB_p_mrmir0000002259	mmu-miR-7231-5p:MIMAT0028430	7.580608	7.146686031	7.302367	4.561899
RB_p_mrmir0000002074	mmu-miR-7232-3p:MIMAT0028433	6.851061	7.398201497	8.153478	2.674538
RB_p_mrmir0000001466	mmu-miR-7232-5p:MIMAT0028432	6.524002	5.136593062	7.098437	7.528959
RB_p_mrmir0000001617	mmu-miR-7233-3p:MIMAT0028435	4.316781	8.17732373	6.566615	2.361174
RB_p_mrmir000000492	mmu-miR-7233-5p:MIMAT0028434	8.469705	6.995666555	7.820899	6.462889
RB_p_mrmir00000015	mmu-miR-7234-3p:MIMAT0028437	10.46812	7.846907903	11.40169	10.47195
RB_p_mrmir0000002281	mmu-miR-7234-5p:MIMAT0028436	7.26944	7.519285532	7.591418	3.475431
RB_p_mrmir0000001789	mmu-miR-7235-3p:MIMAT0028439	6.330045	6.339618591	7.170244	6.448104
RB_p_mrmir0000001264	mmu-miR-7235-5p:MIMAT0028438	14.17451	14.53574963	13.99268	14.50048
RB_p_mrmir0000002247	mmu-miR-7236-3p:MIMAT0028441	8.016874	6.118284582	6.218569	3.014102
RB_p_mrmir000000869	mmu-miR-7236-5p:MIMAT0028440	5.235935	7.490548761	7.571803	4.377106
RB_p_mrmir000000773	mmu-miR-7237-3p:MIMAT0028443	3.520893	6.802202249	6.422635	7.903607
RB_p_mrmir000000578	mmu-miR-7237-5p:MIMAT0028442	9.966247	8.100145105	8.58912	5.779728
RB_p_mrmir0000001075	mmu-miR-7238-3p:MIMAT0028445	1.031158	3.837068276	4.126614	3.03311
RB_p_mrmir000000569	mmu-miR-7238-5p:MIMAT0028444	13.87985	14.449032	13.85342	14.15287
RB_p_mrmir0000002028	mmu-miR-7239-3p:MIMAT0028447	8.656791	7.607114913	5.050199	8.4194
RB_p_mrmir0000001197	mmu-miR-7239-5p:MIMAT0028446	7.79142	8.384455321	5.757002	4.413877
RB_p_mrmir000000917	mmu-miR-7240-3p:MIMAT0028449	7.948261	1.480245932	6.609639	6.956637
RB_p_mrmir0000002248	mmu-miR-7240-5p:MIMAT0028448	8.39135	4.82298442	7.032961	6.6448
RB_p_mrmir0000001554	mmu-miR-7241-3p:MIMAT0028451	10.9046	9.701059362	11.02988	8.753596
RB_p_mrmir000000286	mmu-miR-7241-5p:MIMAT0028450	6.017795	5.571739746	8.869816	3.872167
RB_p_mrmir0000002012	mmu-miR-7242-3p:MIMAT0028453	5.712128	5.097322659	5.304661	3.683158
RB_p_mrmir000000605	mmu-miR-7242-5p:MIMAT0028452	8.026759	7.70403132	7.300343	7.53532
RB_p_mrmir0000001648	mmu-miR-7243-3p:MIMAT0029911	8.062766	7.552760328	7.920616	4.287916
RB_p_mrmir0000001975	mmu-miR-7243-5p:MIMAT0029910	6.074298	9.860410512	6.566698	2.800011
RB_p_mrmir0000001875	mmu-miR-741-3p:MIMAT0004236	5.513069	7.150789851	5.722114	4.989232
RB_p_mrmir0000001479	mmu-miR-741-5p:MIMAT0017262	4.337652	4.291437302	5.536521	1.800702
RB_p_mrmir0000001113	mmu-miR-742-3p:MIMAT0004237	4.748971	3.778941641	3.038952	3.592769
RB_p_mrmir0000001483	mmu-miR-742-5p:MIMAT0004838	3.760329	4.61343707	4.733157	3.927011
RB_p_mrmir0000001109	mmu-miR-743a-3p:MIMAT0004238	7.299778	5.450119295	5.557002	4.800917
RB_p_mrmir0000001610	mmu-miR-743a-5p:MIMAT0017263	5.194095	7.413803769	6.169722	6.235436
RB_p_mrmir0000001111	mmu-miR-743b-3p:MIMAT0004840	6.334542	3.849835288	3.007084	2.244597
RB_p_mrmir0000002152	mmu-miR-743b-5p:MIMAT0004839	6.006986	6.414278725	4.841532	4.657698
RB_p_mrmir0000001083	mmu-miR-744-3p:MIMAT0004820	6.755887	5.316821154	8.232592	7.525644
RB_p_mrmir0000001960	mmu-miR-744-5p:MIMAT0004187	9.299374	8.535697834	7.640738	7.701394
RB_p_mrmir000000787	mmu-miR-7578:MIMAT0029578	6.465141	4.095308215	6.382676	6.187519
RB_p_mrmir0000002315	mmu-miR-758-3p:MIMAT0003889	3.160544	2.356329034	2.768798	1.070357
RB_p_mrmir0000002075	mmu-miR-758-5p:MIMAT0017235	9.534907	6.546307327	9.314315	10.4122

RB_p_mrmir0000001185	mmu-miR-759:MIMAT0003897	4.421437	6.167841076	8.086538	2.783065
RB_p_mrmir000000933	mmu-miR-760-3p:MIMAT0003898	9.075745	7.076065464	8.603513	6.42135
RB_p_mrmir000000848	mmu-miR-760-5p:MIMAT0017245	8.088958	7.284981279	7.012783	8.27585
RB_p_mrmir0000001186	mmu-miR-761:MIMAT0003893	7.380025	7.696366391	7.761618	6.727981
RB_p_mrmir0000001298	mmu-miR-762:MIMAT0003892	12.57914	13.90510486	11.80672	12.59465
RB_p_mrmir000000814	mmu-miR-763:MIMAT0003896	10.17949	8.638563784	9.987554	9.596604
RB_p_mrmir000000381	mmu-miR-764-3p:MIMAT0003895	7.394157	9.225494073	7.439032	4.885139
RB_p_mrmir0000001324	mmu-miR-764-5p:MIMAT0003894	7.915043	6.405299125	4.914154	4.294544
RB_p_mrmir0000001645	mmu-miR-7646-3p:MIMAT0029795	6.645542	7.407528908	3.466579	5.535494
RB_p_mrmir000000156	mmu-miR-7646-5p:MIMAT0029794	5.180771	3.564229836	6.301009	3.18907
RB_p_mrmir000000111	mmu-miR-7647-3p:MIMAT0029797	10.17345	7.538702581	8.547883	8.087661
RB_p_mrmir00000063	mmu-miR-7647-5p:MIMAT0029796	6.742529	8.40175569	8.208269	5.904902
RB_p_mrmir000000426	mmu-miR-7648-3p:MIMAT0029799	12.72876	11.9538704	11.40136	11.93655
RB_p_mrmir000000862	mmu-miR-7648-5p:MIMAT0029798	8.735872	3.322119818	6.542646	8.329599
RB_p_mrmir0000001778	mmu-miR-7649-3p:MIMAT0029801	4.080789	2.531737725	6.339304	4.434837
RB_p_mrmir0000001856	mmu-miR-7649-5p:MIMAT0029800	7.999881	6.366188123	8.683191	7.990313
RB_p_mrmir0000001410	mmu-miR-7650-3p:MIMAT0029803	8.887188	4.424701976	9.236017	8.7423
RB_p_mrmir000000130	mmu-miR-7650-5p:MIMAT0029802	8.19642	6.439855723	8.143554	5.616904
RB_p_mrmir0000001679	mmu-miR-7651-3p:MIMAT0029805	7.778888	5.827939399	5.802194	6.623892
RB_p_mrmir000000117	mmu-miR-7651-5p:MIMAT0029804	6.72326	4.367721804	7.121862	3.910916
RB_p_mrmir0000001811	mmu-miR-7652-3p:MIMAT0029811	7.098743	6.49343525	8.358303	5.449611
RB_p_mrmir0000001432	mmu-miR-7652-5p:MIMAT0029810	7.576928	7.025824828	6.21638	7.195425
RB_p_mrmir000000846	mmu-miR-7653-3p:MIMAT0029813	7.83119	5.997854452	8.66858	7.201611
RB_p_mrmir0000001433	mmu-miR-7653-5p:MIMAT0029812	11.19836	10.27146977	10.81409	11.20869
RB_p_mrmir000000908	mmu-miR-7654-3p:MIMAT0029815	8.514523	5.712354012	8.120844	7.415848
RB_p_mrmir0000001266	mmu-miR-7654-5p:MIMAT0029814	8.180758	5.018312761	5.11407	3.61538
RB_p_mrmir0000002284	mmu-miR-7655-3p:MIMAT0029817	5.799274	6.775257317	6.96823	8.473909
RB_p_mrmir000000925	mmu-miR-7655-5p:MIMAT0029816	7.059852	4.973479131	4.526271	3.692003
RB_p_mrmir000000179	mmu-miR-7656-3p:MIMAT0029819	6.674396	4.794368648	6.362588	3.289462
RB_p_mrmir0000002136	mmu-miR-7656-5p:MIMAT0029818	4.544151	2.715899345	6.245656	6.087093
RB_p_mrmir0000002216	mmu-miR-7657-3p:MIMAT0029821	6.648166	8.111006367	7.325825	3.468963
RB_p_mrmir000000122	mmu-miR-7657-5p:MIMAT0029820	6.230687	6.547011199	4.714439	3.515786
RB_p_mrmir000000196	mmu-miR-7658-3p:MIMAT0029823	6.991324	6.842141489	7.822903	7.232242
RB_p_mrmir0000002137	mmu-miR-7658-5p:MIMAT0029822	13.69226	12.83187083	10.94623	11.27352
RB_p_mrmir000000384	mmu-miR-7659-3p:MIMAT0029825	6.338406	6.130994594	5.358814	7.250132
RB_p_mrmir000000376	mmu-miR-7659-5p:MIMAT0029824	8.304626	7.190047929	4.918771	4.456474
RB_p_mrmir000000559	mmu-miR-7660-3p:MIMAT0029827	6.821214	4.421673674	8.24147	7.337134
RB_p_mrmir0000002305	mmu-miR-7660-5p:MIMAT0029826	6.019305	4.069476759	5.289767	2.328463
RB_p_mrmir0000002234	mmu-miR-7661-3p:MIMAT0029829	6.593034	5.209422419	7.432185	8.586874
RB_p_mrmir00000075	mmu-miR-7661-5p:MIMAT0029828	5.79224	3.438826326	7.57766	7.290339
RB_p_mrmir0000002003	mmu-miR-7662-3p:MIMAT0029831	7.184496	7.703422825	7.054997	3.497351

RB_p_mrmir000000424	mmu-miR-7662-5p:MIMAT0029830	7.596502	4.438316788	8.690521	6.340632
RB_p_mrmir000000490	mmu-miR-7663-3p:MIMAT0029833	6.13323	3.275166099	3.905265	0.626099
RB_p_mrmir0000001240	mmu-miR-7663-5p:MIMAT0029832	7.425433	4.199471497	6.130415	4.044182
RB_p_mrmir0000001784	mmu-miR-7664-3p:MIMAT0029835	5.819378	2.658694594	6.581783	2.572034
RB_p_mrmir000000756	mmu-miR-7664-5p:MIMAT0029834	4.733692	4.092176374	6.893471	1.101409
RB_p_mrmir000000343	mmu-miR-7665-3p:MIMAT0029837	7.253081	7.370890092	6.134419	6.812322
RB_p_mrmir00000099	mmu-miR-7665-5p:MIMAT0029836	12.91664	11.69992997	13.18067	12.50598
RB_p_mrmir0000001168	mmu-miR-7666-3p:MIMAT0029839	11.71616	11.1476839	11.42573	11.056
RB_p_mrmir0000001341	mmu-miR-7666-5p:MIMAT0029838	7.723377	5.563206622	8.529709	6.528422
RB_p_mrmir000000414	mmu-miR-7667-3p:MIMAT0029841	7.089433	5.938856616	8.115839	6.437113
RB_p_mrmir0000001145	mmu-miR-7667-5p:MIMAT0029840	6.712149	8.150804133	8.22191	4.123975
RB_p_mrmir000000946	mmu-miR-7668-3p:MIMAT0029843	10.88473	8.875331397	12.02328	10.96469
RB_p_mrmir000000682	mmu-miR-7668-5p:MIMAT0029842	7.251668	7.303086259	6.797344	7.076817
RB_p_mrmir0000001147	mmu-miR-7669-3p:MIMAT0029845	7.314265	7.410888018	8.794825	8.111007
RB_p_mrmir000000460	mmu-miR-7669-5p:MIMAT0029844	4.865348	6.08042908	5.492527	6.521274
RB_p_mrmir0000001929	mmu-miR-767:MIMAT0012773	2.482693	6.97794409	7.242405	4.272528
RB_p_mrmir0000002204	mmu-miR-7670-3p:MIMAT0029847	5.580971	1.663385609	2.957041	3.17059
RB_p_mrmir000000593	mmu-miR-7670-5p:MIMAT0029846	8.182086	7.811004355	7.854661	7.649011
RB_p_mrmir00000083	mmu-miR-7671-3p:MIMAT0029849	12.19389	12.5258769	12.86201	12.81737
RB_p_mrmir000000407	mmu-miR-7671-5p:MIMAT0029848	3.771305	3.208838121	6.262458	6.303069
RB_p_mrmir0000001745	mmu-miR-7672-3p:MIMAT0029851	6.173289	3.474755208	6.848421	3.466898
RB_p_mrmir000000949	mmu-miR-7672-5p:MIMAT0029850	12.93961	13.52192701	10.93053	11.51529
RB_p_mrmir0000002215	mmu-miR-7673-3p:MIMAT0029853	7.806263	7.392740675	7.153518	5.646815
RB_p_mrmir0000002299	mmu-miR-7673-5p:MIMAT0029852	9.930196	5.157524915	10.49925	9.577822
RB_p_mrmir0000001698	mmu-miR-7674-3p:MIMAT0029857	7.983023	7.694831204	7.99189	6.368983
RB_p_mrmir0000001906	mmu-miR-7674-5p:MIMAT0029856	10.88952	7.933330492	10.28985	9.938255
RB_p_mrmir0000002161	mmu-miR-7675-3p:MIMAT0029859	5.159298	7.381514157	5.162115	1.059626
RB_p_mrmir0000001084	mmu-miR-7675-5p:MIMAT0029858	9.498405	7.752869761	10.21736	9.593307
RB_p_mrmir0000001718	mmu-miR-7676-3p:MIMAT0029861	7.498425	5.707724525	7.368596	6.50735
RB_p_mrmir000000420	mmu-miR-7676-5p:MIMAT0029860	5.683459	7.876577602	5.669564	7.091462
RB_p_mrmir000000864	mmu-miR-7677-3p:MIMAT0029869	4.712325	3.528473265	4.238538	3.882521
RB_p_mrmir000000485	mmu-miR-7677-5p:MIMAT0029868	5.487852	7.779134601	9.181941	7.851947
RB_p_mrmir0000002292	mmu-miR-7678-3p:MIMAT0029871	7.66848	6.951275615	7.707441	3.027914
RB_p_mrmir0000002169	mmu-miR-7678-5p:MIMAT0029870	8.971191	7.075870243	8.356333	8.2733
RB_p_mrmir000000712	mmu-miR-7679-3p:MIMAT0029873	8.464002	5.689352992	7.40279	7.70233
RB_p_mrmir000000409	mmu-miR-7679-5p:MIMAT0029872	6.031143	6.677342987	6.1427	5.692652
RB_p_mrmir000000270	mmu-miR-7680-3p:MIMAT0029875	5.032428	6.529979901	7.710676	3.473
RB_p_mrmir000000595	mmu-miR-7680-5p:MIMAT0029874	7.824961	7.652714927	6.56389	6.532989
RB_p_mrmir000000292	mmu-miR-7681-3p:MIMAT0029883	8.752339	7.707444061	8.858282	8.855771
RB_p_mrmir000000550	mmu-miR-7681-5p:MIMAT0029882	8.052674	5.221048632	4.406286	6.860704
RB_p_mrmir000000892	mmu-miR-7682-3p:MIMAT0029885	8.243047	7.714608155	7.462094	9.41484

RB_p_mrmir000000389	mmu-miR-7682-5p:MIMAT0029884	6.844313	6.772347538	9.439659	6.461963
RB_p_mrmir0000001982	mmu-miR-7683-3p:MIMAT0029887	11.14772	9.604786991	10.9908	11.06585
RB_p_mrmir0000002208	mmu-miR-7683-5p:MIMAT0029886	5.693542	6.821309838	4.616179	5.352306
RB_p_mrmir0000001969	mmu-miR-7684-3p:MIMAT0029891	8.526688	9.054483973	8.228452	6.461907
RB_p_mrmir0000001812	mmu-miR-7684-5p:MIMAT0029890	9.583702	8.862476549	9.996306	9.965463
RB_p_mrmir000000303	mmu-miR-7685-3p:MIMAT0029897	7.832251	2.732655294	7.508731	4.052353
RB_p_mrmir000000214	mmu-miR-7685-5p:MIMAT0029896	3.600956	1.448807581	8.163007	6.454312
RB_p_mrmir0000001042	mmu-miR-7686-3p:MIMAT0029899	7.432176	4.865588659	7.631312	7.92631
RB_p_mrmir000000897	mmu-miR-7686-5p:MIMAT0029898	11.26973	11.38602945	12.6178	12.45508
RB_p_mrmir000000809	mmu-miR-7687-3p:MIMAT0029903	7.735599	9.643701539	6.828293	5.698276
RB_p_mrmir000000404	mmu-miR-7687-5p:MIMAT0029902	10.61133	9.943437984	10.64482	10.51687
RB_p_mrmir0000001810	mmu-miR-7688-3p:MIMAT0029907	2.344905	5.225850218	4.13383	2.847752
RB_p_mrmir0000001527	mmu-miR-7688-5p:MIMAT0029906	7.168033	7.817460718	6.691963	5.625817
RB_p_mrmir0000002174	mmu-miR-7689-3p:MIMAT0029909	4.662809	8.869721304	7.407883	1.064502
RB_p_mrmir000000857	mmu-miR-7689-5p:MIMAT0029908	2.39611	2.70948155	5.62688	2.647442
RB_p_mrmir000000961	mmu-miR-770-3p:MIMAT0003891	10.50646	11.1578535	11.2199	10.34055
RB_p_mrmir000000334	mmu-miR-770-5p:MIMAT0004822	5.347133	3.634940207	4.521875	6.684447
RB_p_mrmir000000625	mmu-miR-7a-1-3p:MIMAT0004670	7.229884	6.636061681	7.603804	7.009681
RB_p_mrmir000000627	mmu-miR-7a-2-3p:MIMAT0017070	7.947143	5.927043187	6.349678	6.761922
RB_p_mrmir0000001984	mmu-miR-7a-5p:MIMAT0000677	5.054756	4.552536772	6.467264	5.277508
RB_p_mrmir000000626	mmu-miR-7b-3p:MIMAT0017071	6.413984	7.227545809	8.704332	7.474975
RB_p_mrmir0000001985	mmu-miR-7b-5p:MIMAT0000678	6.081279	7.089254022	4.616144	4.588413
RB_p_mrmir000000221	mmu-miR-802-3p:MIMAT0017240	8.045294	6.714776557	6.885373	2.883487
RB_p_mrmir0000001665	mmu-miR-802-5p:MIMAT0004188	3.952742	5.335278634	5.492702	3.123206
RB_p_mrmir0000002119	mmu-miR-804:MIMAT0004210	5.862071	4.148764036	6.299106	7.463771
RB_p_mrmir0000001121	mmu-miR-8090:MIMAT0031391	8.269428	5.212648372	7.895175	7.974839
RB_p_mrmir000000385	mmu-miR-8091:MIMAT0031392	9.018493	8.247024569	8.517936	8.717572
RB_p_mrmir0000001198	mmu-miR-8092:MIMAT0031393	6.121787	5.157732392	4.486715	7.019704
RB_p_mrmir0000002261	mmu-miR-8093:MIMAT0031394	8.199011	8.26530973	9.0413	7.437961
RB_p_mrmir00000067	mmu-miR-8094:MIMAT0031395	10.17692	7.72966961	10.74318	10.16927
RB_p_mrmir00000017	mmu-miR-8095:MIMAT0031396	8.219771	3.083160067	6.144342	5.762725
RB_p_mrmir0000001291	mmu-miR-8096:MIMAT0031398	9.985643	6.44766556	8.749444	8.956706
RB_p_mrmir00000038	mmu-miR-8097:MIMAT0031399	7.405523	8.225282821	7.71472	6.126807
RB_p_mrmir0000001226	mmu-miR-8098:MIMAT0031400	4.950712	7.122667421	6.21215	2.883098
RB_p_mrmir000000800	mmu-miR-8099:MIMAT0031401	5.913909	8.361966891	8.979895	5.77357
RB_p_mrmir000000379	mmu-miR-8100:MIMAT0031403	14.65081	13.89067584	14.45721	14.43386
RB_p_mrmir0000001276	mmu-miR-8101:MIMAT0031405	12.18833	13.2367528	14.92178	14.83309
RB_p_mrmir0000001642	mmu-miR-8102:MIMAT0031406	12.98842	12.1287623	13.22993	12.86955
RB_p_mrmir0000001775	mmu-miR-8103:MIMAT0031407	6.692839	4.63364988	6.774324	4.326032
RB_p_mrmir000000715	mmu-miR-8104:MIMAT0031408	12.25071	11.34534218	12.72624	12.00017
RB_p_mrmir000000400	mmu-miR-8105:MIMAT0031409	9.772413	10.43188215	8.627371	9.109537

RB_p_mrmir0000001864	mmu-miR-8106:MIMAT0031411	6.748585	5.931787922	5.109244	-0.40438
RB_p_mrmir0000001980	mmu-miR-8107:MIMAT0031412	11.59038	10.14147777	10.18562	10.00828
RB_p_mrmir0000001814	mmu-miR-8108:MIMAT0031413	9.080734	7.764750622	8.474516	8.790081
RB_p_mrmir0000001218	mmu-miR-8109:MIMAT0031415	8.837995	5.878718865	6.976279	6.75697
RB_p_mrmir00000084	mmu-miR-8110:MIMAT0031416	14.62654	14.17763586	13.86601	13.92507
RB_p_mrmir000000205	mmu-miR-8111:MIMAT0031417	7.045618	3.668493681	6.680578	4.8649
RB_p_mrmir0000001774	mmu-miR-8112:MIMAT0031418	7.296579	6.647660521	4.656207	6.93437
RB_p_mrmir000000709	mmu-miR-8113:MIMAT0031419	11.02339	9.461719156	10.83381	10.6886
RB_p_mrmir0000001634	mmu-miR-8114:MIMAT0031420	7.260604	6.277805694	5.15397	7.379962
RB_p_mrmir000000859	mmu-miR-8115:MIMAT0031421	7.213545	6.85359014	3.747704	2.709209
RB_p_mrmir00000096	mmu-miR-8116:MIMAT0031422	7.972426	7.067790777	6.210584	5.314859
RB_p_mrmir0000001230	mmu-miR-8117:MIMAT0031423	14.29909	14.43093308	14.83286	14.81539
RB_p_mrmir0000001132	mmu-miR-8118:MIMAT0031424	6.148103	3.399211767	5.923883	3.069957
RB_p_mrmir0000001153	mmu-miR-8119:MIMAT0031425	11.02986	9.620479294	11.45937	9.943493
RB_p_mrmir0000002125	mmu-miR-8120:MIMAT0031426	6.858253	4.789966162	6.900456	5.823677
RB_p_mrmir0000001866	mmu-miR-871-3p:MIMAT0017265	4.011754	6.126349247	6.370408	5.075095
RB_p_mrmir0000001608	mmu-miR-871-5p:MIMAT0004841	4.120933	4.905617729	5.3572	4.938959
RB_p_mrmir0000001843	mmu-miR-872-3p:MIMAT0004935	6.312849	4.495082105	5.439235	3.405358
RB_p_mrmir000000108	mmu-miR-872-5p:MIMAT0004934	4.378676	5.324907796	6.653815	2.704955
RB_p_mrmir0000001140	mmu-miR-873a-3p:MIMAT0017279	8.671184	9.049399509	5.23159	1.446796
RB_p_mrmir0000001191	mmu-miR-873a-5p:MIMAT0004936	4.511366	3.542137455	5.860963	4.11419
RB_p_mrmir000000162	mmu-miR-873b:MIMAT0025177	6.237074	3.881110106	7.074096	8.335143
RB_p_mrmir0000001036	mmu-miR-874-3p:MIMAT0004853	9.381523	4.051505382	7.850948	8.580359
RB_p_mrmir000000927	mmu-miR-874-5p:MIMAT0017268	8.738349	7.809442299	6.839684	8.097147
RB_p_mrmir000000880	mmu-miR-875-3p:MIMAT0004938	4.050374	3.325677589	7.80876	5.488186
RB_p_mrmir0000001572	mmu-miR-875-5p:MIMAT0004937	1.731169	5.727570685	4.307912	7.190699
RB_p_mrmir0000001555	mmu-miR-876-3p:MIMAT0004855	4.705657	4.993148269	6.117892	-0.27818
RB_p_mrmir0000002010	mmu-miR-876-5p:MIMAT0004854	4.273787	1.557782778	4.628586	3.48841
RB_p_mrmir0000002103	mmu-miR-877-3p:MIMAT0004862	7.271964	4.810084554	5.680046	5.199958
RB_p_mrmir0000001340	mmu-miR-877-5p:MIMAT0004861	12.4848	12.03237501	13.44184	12.54345
RB_p_mrmir0000001194	mmu-miR-878-3p:MIMAT0004933	8.053825	6.326376315	7.751728	5.648217
RB_p_mrmir0000001577	mmu-miR-878-5p:MIMAT0004932	2.879741	2.718096928	5.652647	5.311986
RB_p_mrmir0000001251	mmu-miR-879-3p:MIMAT0004843	3.191647	5.236964463	8.555393	-0.12486
RB_p_mrmir000000313	mmu-miR-879-5p:MIMAT0004842	5.016195	7.294490721	4.275453	-0.34439
RB_p_mrmir0000001489	mmu-miR-880-3p:MIMAT0004844	6.029582	6.574058721	6.517627	2.640366
RB_p_mrmir0000001484	mmu-miR-880-5p:MIMAT0017266	5.168027	8.686389079	4.762316	2.461274
RB_p_mrmir00000071	mmu-miR-881-3p:MIMAT0004846	5.440529	4.693683556	4.648757	4.313568
RB_p_mrmir000000690	mmu-miR-881-5p:MIMAT0004845	6.219154	4.847511144	6.937075	7.219258
RB_p_mrmir000000375	mmu-miR-882:MIMAT0004847	7.280306	8.662679553	6.577001	7.193363
RB_p_mrmir0000001427	mmu-miR-883a-3p:MIMAT0004849	6.90817	2.032698744	6.700482	4.968778
RB_p_mrmir0000001970	mmu-miR-883a-5p:MIMAT0004848	8.632713	8.59593636	8.460952	8.775901

RB_p_mrmir0000001428	mmu-miR-883b-3p:MIMAT0004851	5.887701	4.986835763	7.767757	1.151137
RB_p_mrmir0000001492	mmu-miR-883b-5p:MIMAT0004850	7.68163	7.015348299	7.607143	8.618248
RB_p_mrmir000000456	mmu-miR-92a-1-5p:MIMAT0017066	7.489275	4.799483294	7.183252	0.437119
RB_p_mrmir000000448	mmu-miR-92a-2-5p:MIMAT0004635	10.39823	8.309726263	8.264717	8.663593
RB_p_mrmir0000001614	mmu-miR-92a-3p:MIMAT0000539	7.02236	6.538402694	4.8085	5.070745
RB_p_mrmir0000001613	mmu-miR-92b-3p:MIMAT0004899	6.87996	8.459541552	7.467919	5.479164
RB_p_mrmir000000413	mmu-miR-92b-5p:MIMAT0017278	11.704	10.53168947	10.17873	10.77204
RB_p_mrmir000000266	mmu-miR-93-3p:MIMAT0004636	5.978783	6.707175004	6.454419	3.836354
RB_p_mrmir000000840	mmu-miR-935:MIMAT0035718	6.59301	7.227213315	4.789069	6.38546
RB_p_mrmir000000619	mmu-miR-93-5p:MIMAT0000540	6.625554	7.572797834	8.245701	5.051872
RB_p_mrmir000000505	mmu-miR-9-3p:MIMAT0000143	6.881989	5.342994223	4.08892	5.74358
RB_p_mrmir0000001831	mmu-miR-9-5p:MIMAT0000142	4.329575	4.347095799	3.801934	4.44918
RB_p_mrmir000000649	mmu-miR-96-3p:MIMAT0017021	5.004977	1.25009862	3.677127	2.744565
RB_p_mrmir0000002308	mmu-miR-96-5p:MIMAT0000541	6.613704	5.385627296	7.852798	6.604492
RB_p_mrmir000000265	mmu-miR-9768-3p:MIMAT0036460	6.575048	6.016560941	7.388716	6.988293
RB_p_mrmir0000002011	mmu-miR-9768-5p:MIMAT0036459	8.852934	7.924749489	9.074554	7.704728
RB_p_mrmir0000001544	mmu-miR-9769-3p:MIMAT0036462	7.76665	6.362750395	4.315787	6.200376
RB_p_mrmir000000388	mmu-miR-9769-5p:MIMAT0036461	7.775361	9.927035581	7.8382	8.151587
RB_p_mrmir000000977	mmu-miR-98-3p:MIMAT0017023	6.692785	4.090948148	4.78195	2.778914
RB_p_mrmir0000001897	mmu-miR-98-5p:MIMAT0000545	3.47131	4.954056583	5.871726	7.750142
RB_p_mrmir000000641	mmu-miR-99a-3p:MIMAT0016981	6.531181	7.156949969	6.411166	4.057481
RB_p_mrmir00000055	mmu-miR-99a-5p:MIMAT0000131	6.265857	6.690543788	4.07638	4.950372
RB_p_mrmir000000640	mmu-miR-99b-3p:MIMAT0004525	7.987806	7.696701557	6.919419	5.88724
RB_p_mrmir000000673	mmu-miR-99b-5p:MIMAT0000132	7.69899	5.465976402	7.103187	8.137581
